# Treatment Outcomes Among Patients With a Positive *Candida* Culture Close to Randomization Receiving Rezafungin or Caspofungin in the ReSTORE Study

**DOI:** 10.1093/cid/ciae363

**Published:** 2024-07-10

**Authors:** Alex Soriano, Patrick M Honore, Oliver A Cornely, Methee Chayakulkeeree, Matteo Bassetti, Huang Haihui, Hervé Dupont, Young Keun Kim, Marin Kollef, Bart Jan Kullberg, Nick Manamley, Peter Pappas, John Pullman, Taylor Sandison, Cecilia Dignani, Jose A Vazquez, George R Thompson

**Affiliations:** Hospital Clínic de Barcelona, IDIBAPS, University of Barcelona, Barcelona, Spain; CIBERINF, CIBER of Infectious Diseases, Madrid, Spain; CHU UCL Godinne Namur, UCL Louvain Medical School, Campus Godinne, Namur, Belgium; Faculty of Medicine Institute of Translational Research, Cologne Excellence Cluster on Cellular Stress Responses in Aging-Associated Diseases (CECAD), University of Cologne, Cologne, Germany; Department I of Internal Medicine, Excellence Center for Medical Mycology (ECMM) and German Center for Infection Research (DZIF), Partner Site Bonn-Cologne, University Hospital Cologne, Cologne, Germany; Division of Infectious Diseases and Tropical Medicine, Department of Medicine, Faculty of Medicine Siriraj Hospital, Mahidol University, Bangkok, Thailand; Infectious Diseases Unit, IRCCS San Martino Polyclinic Hospital, Genoa, Italy; Department of Health Sciences (DISSAL), University of Genoa, Genoa, Italy; Institute of Antibiotics, Huashan Hospital, Fudan University, Shanghai, China; Amiens-Picardie University Hospital, Amiens, France; Department of Internal Medicine, Yonsei University Wonju College of Medicine, Wonju, South Korea; Washington University, St. Louis, Missouri, USA; Radboud University Medical Center, Nijmegen, The Netherlands; Mundipharma Research Limited, Cambridge, United Kingdom; University of Alabama at Birmingham, Birmingham, Alabama, USA; Mercury Street Medical, Butte, Montana, USA; Cidara Therapeutics, Inc., San Diego, California, USA; PSI-CRO, Durham, North Carolina, USA; Augusta University, Augusta, Georgia, USA; University of California Davis Medical Center, Sacramento, California, USA

**Keywords:** candidemia, rezafungin, invasive candidiasis, echinocandin, caspofungin

## Abstract

**Background:**

Rezafungin, a novel, once-weekly echinocandin for the treatment of candidemia and/or invasive candidiasis (IC) was noninferior to caspofungin for day 30 all-cause mortality (ACM) and day 14 global cure in the phase 3 ReSTORE trial (NCT03667690). We conducted preplanned subgroup analyses for patients with a positive culture close to randomization in ReSTORE.

**Methods:**

ReSTORE was a multicenter, double-blind, double-dummy, randomized trial in patients aged ≥18 years with candidemia and/or IC treated with once-weekly intravenous rezafungin (400 mg/200 mg) or once-daily intravenous caspofungin (70 mg/50 mg). This analysis comprised patients with a positive blood culture drawn between 12 hours before and 72 hours after randomization or a positive culture from another normally sterile site sampled between 48 hours before and 72 hours after randomization. Efficacy endpoints included day 30 ACM, day 14 global cure rate, and day 5 and 14 mycological response. Adverse events were evaluated.

**Results:**

This analysis included 38 patients randomized to rezafungin and 46 to caspofungin. In the rezafungin and caspofungin groups, respectively, day 30 ACM was 26.3% and 21.7% (between-group difference [95% confidence interval], 4.6% [−13.7%, 23.5%]), day 14 global response was 55.3% and 50.0% (between-group difference, 5.3% [−16.1%, 26.0%]), and day 5 mycological eradication was 71.1% and 50.0% (between-group difference, 21.1% [−0.2%, 40.2%]). Safety was comparable between treatments.

**Conclusions:**

These findings support the efficacy and safety of rezafungin compared with caspofungin for the treatment of candidemia and/or IC in patients with a positive culture close to randomization, with potential early treatment benefits for rezafungin.

## Teaser

This preplanned subgroup analysis of the ReSTORE trial supports rezafungin efficacy and safety versus caspofungin for the treatment of candidemia and/or invasive candidiasis in patients with a positive culture close to randomization, who may represent patients with more serious infections.

## Graphical Abstract

**Graphical Abstract:**
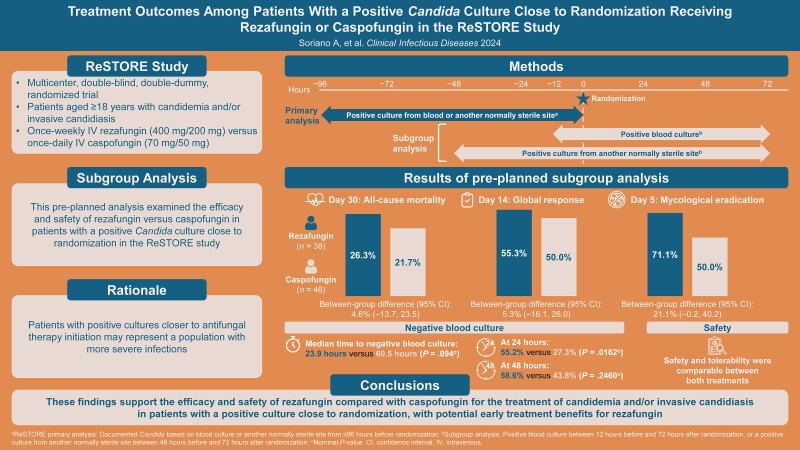

Candidemia and invasive candidiasis (IC) are common healthcare facility–associated fungal infections that have a substantial impact on patient morbidity and mortality and a high economic burden [1–5]. Echinocandins are recommended as first-line antifungal treatment for candidemia and IC in Europe and the United States [6–8] based on their efficacy and relatively fewer adverse effects and drug–drug interactions compared with polyenes and triazoles. However, the emergence of antifungal resistance among *Candida* species mandates the need for novel antifungal drugs [2].

Rezafungin is a novel, US Food and Drug Administration (FDA)– and European Commission–approved echinocandin [9, 10] that is structurally similar to current echinocandins but has differentiated stability and pharmacokinetics [8, 11–13]. Its low clearance and prolonged half-life versus other echinocandins enables once-weekly intravenous administration resulting in front-loaded exposure that maximizes the drug effect early in therapy [13]. These pharmacokinetic advantages may suppress the development of secondary antifungal resistance [13–15]. The safety and efficacy of rezafungin in treating candidemia and/or IC were demonstrated in the phase 2, double-blind, randomized STRIVE trial comparing rezafungin and caspofungin [16]. Primary data from the similarly designed phase 3 ReSTORE trial demonstrated noninferiority of rezafungin for day 30 all-cause mortality (ACM) and day 14 global cure versus caspofungin for the treatment of candidemia and/or IC [17]. Both trials suggested early benefits of rezafungin versus caspofungin, including more rapid clearance of candidemia, and reported similar safety profiles for rezafungin and caspofungin [16, 17].

As with prior randomized trials evaluating echinocandins [18–20], patients in the ReSTORE trial could be randomized for up to 4 days after a positive *Candida* culture [17]. Patients were also allowed a maximum of 48 hours of empiric antifungal treatment before enrollment [17–20]. Culture results obtained closer to the initiation of antifungal therapy could theoretically have been negative, either spontaneously or due to empiric therapy. It is important to understand the potential clinical impact of initiation of study therapy and the timing of blood/tissue cultures because patients with positive cultures closer to antifungal therapy initiation may represent a population with more severe infections.

This preplanned analysis examined the efficacy and safety of rezafungin versus caspofungin in a subgroup of patients in the ReSTORE trial with a positive culture close to randomization.

## METHODS

### Study Design and Participants

**Figure 1.**
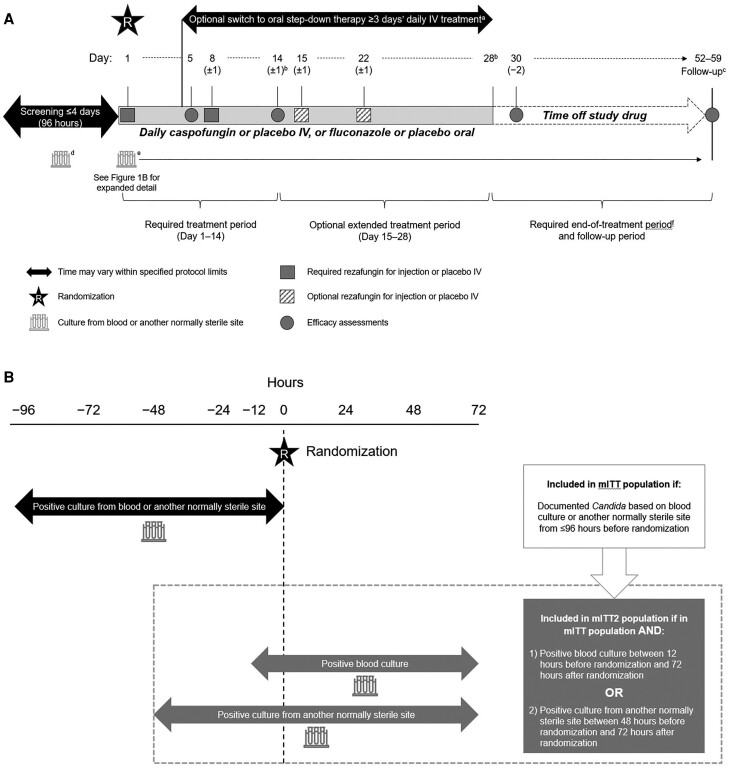
*A*, ReSTORE study design. *B*, mITT and mITT2 population definitions. ^a^After ≥3 days of IV study drug (or the minimum duration of IV therapy advised by the site's national/regional/local guidelines, whichever was greater), patients were permitted to switch to oral step-down therapy as long as all criteria specified in the protocol were met. Patients who were switched to oral step-down therapy could switch back to IV study drug therapy in the event of the development of a condition that prevented the subject from taking oral medication (eg, pancreatitis, urgent surgery), but were not permitted to switch back to IV study drug therapy for relapse of candidemia/IC or for intolerance or toxicity due to the study drug. ^b^The last required dose of the study drug was on day 14 and the last possible dose of the study drug was on day 28. ^c^Follow-up occurred between days 52 and 59. Patients who stopped the study drug early (ie, clinical failures) and required a change in antifungal therapy to treat candidemia and/or IC were permitted to have an earlier follow-up visit occurring ≥30 days from the last weekly dose of IV rezafungin or IV placebo. ^d^If the positive blood culture used to qualify the patient for the study (ie, screening culture) was drawn >12 hours prior to randomization, an additional set of blood cultures was to be obtained ≤12 hours before randomization to determine whether patients were still candidemic at the time of enrollment. ^e^Blood cultures were to be repeated daily (preferred) or every other day until the first negative blood culture result for *Candida* spp. with no subsequent positive culture (in cases when ≥1 samples were drawn and cultured after the first negative culture was available). ^f^Patients completed an end-of-treatment visit ≤2 days after the last dose of study drug. All safety assessments were completed at the end-of-treatment visit. Efficacy assessments were also to be completed at the end-of-treatment visit. Abbreviations: IC, invasive candidiasis; IV, intravenous; mITT, modified intention-to-treat; mITT2, modified intention-to-treat 2.

Full methodological details of the ReSTORE trial (NCT03667690) and primary data have been reported [17]. ReSTORE was a multicenter, prospective, randomized, double-blind, double-dummy, noninferiority phase 3 study comparing rezafungin with caspofungin for the treatment of adults aged 18 years and older with candidemia and/or IC. Mycological diagnosis of candidemia and/or IC was from a blood or normally sterile site sample collected 96 hours or less before randomization (Figure 1*A*). Patients had 1 or more systemic signs attributable to candidemia/IC (eg, fever, hypothermia, tachycardia, tachypnea, local signs of inflammation) appearing from 12 hours before the qualifying positive culture through the time of randomization.

This preplanned analysis examined data for a subgroup of patients in the modified intention-to-treat (mITT) population (mITT2). These patients had either (1) a positive blood culture drawn between 12 hours before and 72 hours after randomization or (2) a positive culture from another normally sterile site sampled between 48 hours before and 72 hours after randomization. The different time cutoffs used for the 2 sites reflect differences in the relative difficulty of obtaining samples. All patients must have received 1 or more dose of study drug (Figure 1*B*).

Patients were randomly assigned (1:1) to receive once-weekly intravenous rezafungin (400 mg on day 1, 200 mg on day 8, and optional 200-mg doses on day 15 and day 22) or once-daily intravenous caspofungin (70 mg on day 1, 50 mg on days 2–28 [with dose adjustment according to hepatic impairment, drug–drug interactions, or patient weight in accordance with the approved labeling and at the investigator's discretion]) for 14–28 days. Patients in the rezafungin group received intravenous placebo on the other study days to maintain blinding. Patients in both groups who met relevant criteria could step down to oral therapy after 3 or more days of intravenous therapy (rezafungin group: placebo; caspofungin group: fluconazole).

### Study Assessments

Efficacy endpoints included ACM at day 30 (primary efficacy outcome of ReSTORE as mandated by the FDA) and global response at days 5 and 14 visits (primary efficacy outcome of ReSTORE as mandated by the European Medicines Agency). The ACM endpoint comprised patients who died on or before day 30 or whose survival status was unknown. Patients who were alive at day 28/day 29 but had unknown survival status at day 30 were considered alive for the purposes of this assessment. Global response was based on clinical cure as assessed by the investigator, radiological cure (for patients with IC), and mycological eradication, all of which were confirmed by an independent, blinded Data Review Committee (DRC). Outcomes according to the DRC were used for this analysis.

Additional efficacy endpoints included mycological eradication at days 5 and 14, time to first negative blood culture (TTNBC), and the percentage of negative blood cultures (NBCs) at 24 and 48 hours after the first dose of the study drug. For patients with a positive blood culture at baseline, mycological response was defined as eradication if the last blood culture drawn on or prior to the day of assessment was negative with no subsequent positive culture from a sample drawn after the first dose of the study drug. For patients with a positive culture at baseline from a normally sterile site other than blood, mycological eradication could be either documented (negative culture from the same normally sterile site on or prior to the day of assessment [ie, day 5 or day 14]) or presumed (assessment of clinical and radiological cure [for patients with evidence of disease according to imaging at baseline]) if a culture specimen from the infected site was not available. Mycological failure was defined as documented or presumed fungal persistence, change of antifungal therapy to treat candidemia and/or IC, or death from any cause before or on the day of assessment. Indeterminate mycological response was defined as the unavailability of study data for efficacy evaluation for any reason (eg, culture specimen or result not available or patient lost to follow-up). The TTNBC (for patients enrolled with a positive blood culture) was calculated as the time from the first dose of the study drug to the first NBC without subsequent positive culture. Blood cultures were repeated daily or every other day until the first NBC result for *Candida* spp. with no subsequent positive culture.

Safety endpoints included treatment-emergent adverse events (TEAEs), drug-related TEAEs, serious adverse events (SAEs), and drug-related SAEs. A TEAE was defined as an adverse event that occurred during or after study drug administration and up to the follow-up visit. Safety was also assessed through evaluation of clinical laboratory data. Adverse events and abnormal laboratory values were graded for severity using the National Cancer Institute Common Terminology Criteria for Adverse Events (NCI-CTCAE) version 5.0 and coded using the Medical Dictionary for Regulatory Activities (MedDRA; version 23.0 or higher).

### Data Analyses

All endpoints were analyzed in the mITT2 population.

Efficacy endpoints were evaluated using a 2-sided 95% confidence interval (CI) calculated using the unadjusted methodology of Miettinen and Nurminen. *P* values presented are nominal and were not adjusted for multiplicity.

Safety endpoints were summarized using descriptive statistics.

### Study Oversight

The ReSTORE trial was conducted in accordance with current regulations, the International Conference on Harmonisation Good Clinical Practice, and Declaration of Helsinki. Independent ethics committees or institutional review boards at participating sites approved the protocol and all amendments. All patients, or their legally authorized representative, provided written informed consent.

## RESULTS

### Demographics and Baseline Characteristics

**Figure 2.**
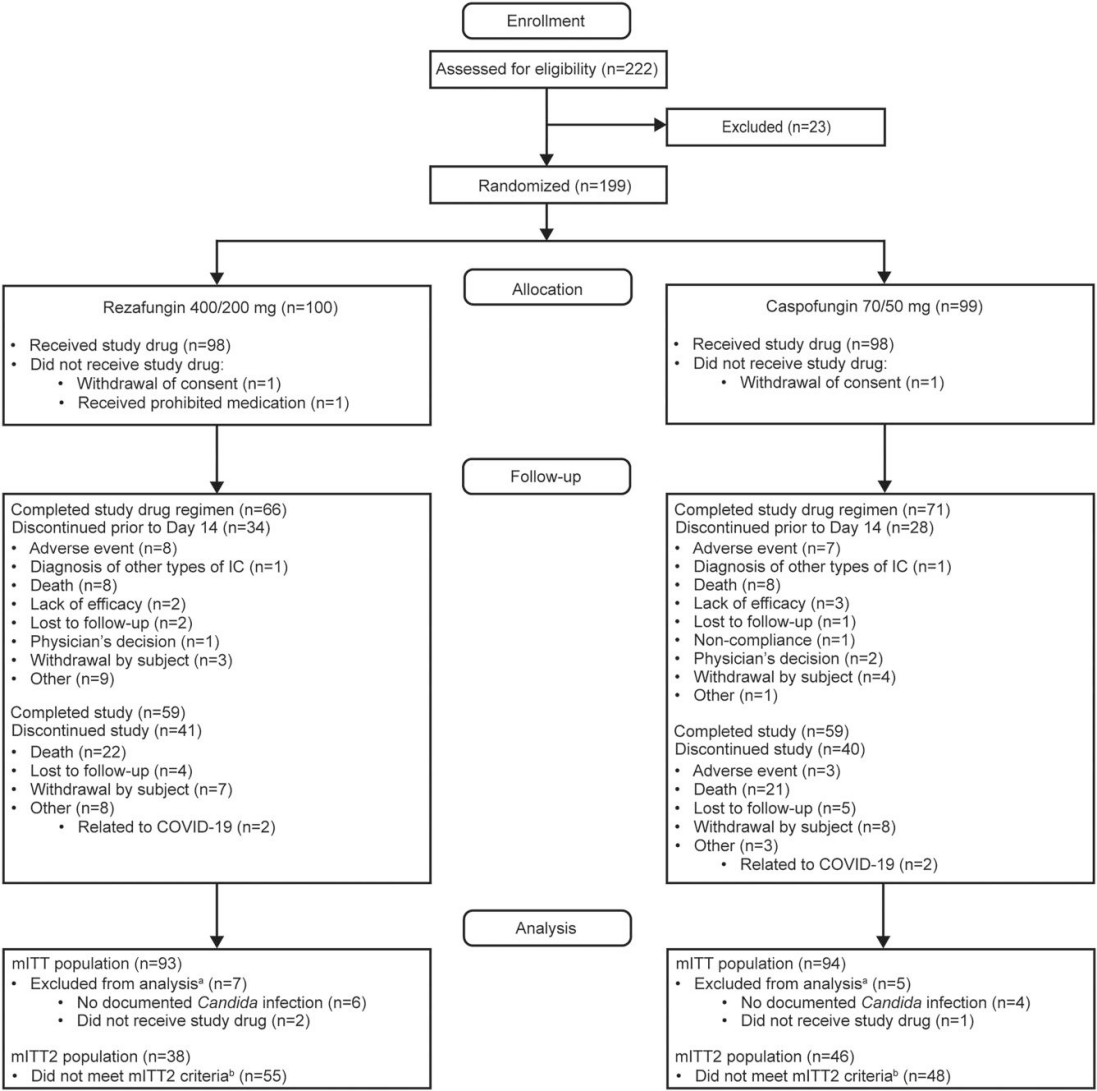
Patient disposition in ReSTORE indicating mITT and mITT2 analysis populations. ^a^Some patients met multiple reasons for exclusion and are included with each exclusion reason met. ^b^Patients with either (1) a positive blood culture drawn between 12 hours before and 72 hours after randomization or (2) a positive culture from another normally sterile site sampled between 48 hours before and 72 hours after randomization and who received ≥1 dose of study drug were included in the mITT2 analysis. Figure modified from Thompson GR III et al. Rezafungin versus caspofungin for treatment of candidaemia and invasive candidiasis (ReSTORE): a multicentre, double-blind, double-dummy, randomised phase 3 trial. Lancet 2023; 401:49–59, with permission from Elsevier. ©2022 Elsevier Ltd. Abbreviations: COVID-19, coronavirus disease 2019; mITT, modified intention-to-treat; mITT2, modified intention-to-treat 2.

Of the 187 patients in the ReSTORE mITT population with mycologically confirmed candidemia and/or IC, 84 were eligible for inclusion in the mITT2 population. This population comprised 38 patients randomly assigned to rezafungin and 46 assigned to caspofungin (Figure 2). Baseline demographics and characteristics were generally well balanced between groups (Table 1). The mean age of patients was approximately 60 years, most patients were male and White, and the majority of patients in both groups were enrolled due to candidemia alone (rezafungin: 76.3%; caspofungin: 71.7%). In patients with IC, the most common site of infection was intra-abdominal, including the peritoneal space. At enrollment, 16 of 38 (42.1%) and 21 of 46 (45.7%) patients in the rezafungin and caspofungin groups, respectively, were in an intensive care unit. Most patients (86.5% [32/37] in the rezafungin group and 76.1% [35/46] in the caspofungin group) had a modified Acute Physiology and Chronic Health Evaluation (APACHE) II score (APACHE II + [15 minus Glasgow Coma Score]) of less than 20; median was 13 in both groups. At baseline, 23.7% (9/38) and 32.6% (15/46) of patients in the rezafungin and caspofungin groups, respectively, were mechanically ventilated. Most patients had a central venous catheter (CVC) at baseline (71.1% [27/38] in the rezafungin and 60.9% [28/46] in the caspofungin groups, respectively); approximately one-fifth of patients had a peripherally inserted central catheter. Among patients with a CVC at baseline, fewer patients in the rezafungin group (7.4% [2/27]) had this removed within 48 hours of the first positive *Candida* culture compared with the caspofungin group (32.1% [9/28]). *Candida* species were similarly distributed across the 2 groups; the most frequently isolated species were *Candida albicans*, *Candida glabrata*, *Candida tropicalis*, and *Candida parapsilosis* complex.

**Table 1.** Baseline Demographics and Characteristics (mITT2 Population)

	Rezafungin (400/200 mg) (N = 38)	Caspofungin (70/50 mg) (N = 46)
Age, mean ± SD (range), y	58.9 ± 14.11 (27, 87)	62.9 ± 14.55 (20, 87)
<65 y, n (%)	24 (63.2)	25 (54.3)
≥65 y, n (%)	14 (36.8)	21 (45.7)
Gender, n (%)		
Male	26 (68.4)	27 (58.7)
Female	12 (31.6)	19 (41.3)
Race, n (%)		
White	26 (70.3)	27 (60.0)
Asian	9 (24.3)	16 (35.6)
African American or Black	2 (5.4)	1 (2.2)
Not reported	1	1
Final diagnosis, n (%)		
Candidemia	29 (76.3)	33 (71.7)
Invasive candidiasis^a^	9 (23.7)	13 (28.3)
Site of infection for invasive candidiasis, n (%)	n = 9	n = 13
Intra-abdominal (including peritoneal space)^b^	5 (62.5)	8 (61.5)
Catheter tip	0	1 (7.7)
Pancreas (swab)	0	1 (7.7)
Gallbladder, bile	1 (12.5)	0
Pancreatic liquid	0	1 (7.7)
Soft tissue	2 (22.2)	2 (15.4)
Body mass index,^c^ n (%)		
<18.5 kg/m^2^	4 (11.1)	7 (17.1)
≥18.5 to <25 kg/m^2^	16 (44.4)	20 (48.8)
≥25 to <30 kg/m^2^	3 (8.3)	9 (22)
≥30 to <40 kg/m^2^	11 (30.6)	4 (9.8)
>40 kg/m^2^	2 (5.6)	1 (2.4)
Modified APACHE II score^d^		
≥20, n (%)	5 (13.5)	11 (23.9)
<20, n (%)	32 (86.5)	35 (76.1)
Median (range)	13.0 (3–40)	13.0 (2–37)
ANC <500/μL, n (%)	4 (10.8)	4 (8.7)
Mechanically ventilated at baseline, n (%)	9 (23.7)	15 (32.6)
Peripherally inserted central catheter, n (%)	7 (18.4)	9 (19.6)
Central venous catheter, n (%)	27 (71.1)	28 (60.9)
Central venous catheter removed within 48 h of first positive *Candida* culture, n (%)		
Yes	2 (7.4)	9 (32.1)
No	25 (92.6)	19 (67.9)
Parenteral nutrition, n (%)	0	1 (2.2)
Hemodialysis, n (%)	6 (15.8)	4 (8.7)
Received prior immunosuppressants, n (%)	6 (15.8)	3 (6.5)
Received prior norepinephrine, n (%)	6 (15.8)	4 (8.7)
*Candida* species,^e^ n (%)		
*Candida albicans*	17 (44.7)	21 (45.7)
*Candida glabrata*	8 (21.1)	13 (28.3)
*Candida tropicalis*	7 (18.4)	10 (21.7)
*Candida parapsilosis* complex	5 (13.2)	7 (15.2)
*Candida krusei*	2 (5.3)	2 (4.3)
*Candida dubliniensis*	2 (5.3)	0

The mITT2 population are patients who had either (1) a positive culture from blood drawn between 12 hours before and 72 hours after randomization or (2) a positive culture from another normally sterile site between 48 hours before and 72 hours after randomization. All patients must have received at least 1 dose of study drug

Abbreviations: ANC, absolute neutrophil count; APACHE, Acute Physiology and Chronic Health Evaluation; mITT2, modified intention-to-treat 2.

^a^Patients who progressed from candidemia to invasive candidiasis based on radiological and/or tissue/fluid culture assessment through day 14.

^b^Patients with intra-abdominal candidiasis who had concomitant intra-abdominal bacterial infection were not excluded from the study.

^c^Two patients in the rezafungin group and 5 patients in the caspofungin group had missing body mass index data.

^d^Modified APACHE II score is a combination of APACHE II and Glasgow Coma Score and is calculated as APACHE II+ (15 minus Glasgow Coma Score). One patient in the rezafungin group had missing APACHE II score data.

^e^Some patients had multiple pathogens at baseline.

### Efficacy of Rezafungin Versus Caspofungin

At day 30, 26.3% (10/38) and 21.7% (10/46) of patients in the rezafungin and caspofungin groups, respectively, were either known to have died or had unknown survival status (3 patients had unknown status [rezafungin: 1, caspofungin: 2]). The treatment difference (95% CI) for day 30 ACM was 4.6% (−13.7%, 23.5%).

**Figure 3.**
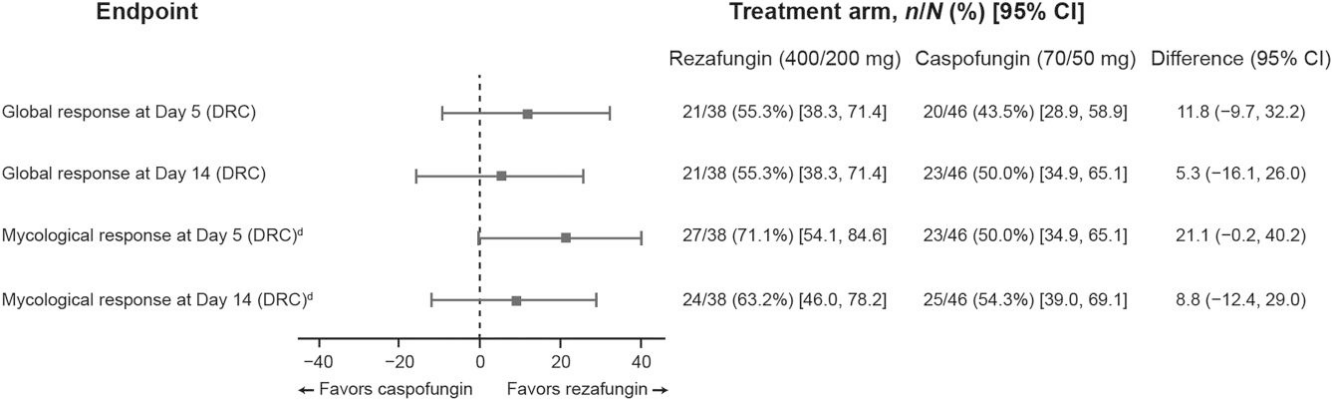
Global response and mycological eradication at days 5 and 14 in patients treated with rezafungin (400 mg/200 mg) or caspofungin (70 mg/50 mg) (mITT2 population). Based on clinical cure (as assessed by the investigator, radiological cure [for patients with invasive candidiasis], and mycological eradication) confirmed by an independent, blinded DRC. For patients with a positive blood culture at baseline, mycological eradication was defined as a negative blood culture on or prior to the day of assessment (ie, day 5 or 14) with no subsequent positive culture. For patients with a positive culture at baseline from a normally sterile site other than blood, it was either documented (a negative culture from the same normally sterile site on or prior to the day of assessment [ie, day 5 or day 14]) or presumed (assessment of clinical and radiological cure [for those with evidence of disease on imaging at baseline] if a specimen from the infected site was not available). Patients with either (1) a positive blood culture drawn between 12 hours before and 72 hours after randomization or (2) a positive culture from another normally sterile site sampled between 48 hours before and 72 hours after randomization and who received ≥1 dose of the study drug. These were not mutually exclusive outcomes; eradication at day 5 did not guarantee eradication at day 14. Only 1 patient was classified as a relapse between day 5 and day 14: a patient in the caspofungin group had documented eradication at day 5 but then had breakthrough candidemia on days 9 and 11. Abbreviations: CI, confidence interval; DRC, Data Review Committee; mITT2, modified intention-to-treat 2.

At day 5, the global cure rate was 55.3% (21/38) and 43.5% (20/46) in the rezafungin and caspofungin groups, respectively (treatment difference [95% CI]: 11.8% [−9.7%, 32.2%]); by day 14, this was 55.3% (21/38) and 50.0% (23/46), with a treatment difference of 5.3% (−16.1%, 26.0%) (Figure 3). At day 5, the proportion of patients with mycological eradication was 71.1% (27/38) and 50.0% (23/46) in the rezafungin and caspofungin groups, respectively, with a treatment difference (95% CI) of 21.1% (−0.2%, 40.2%); by day 14, it was 63.2% (24/38) and 54.3% (25/46), with a treatment difference of 8.8% (−12.4%, 29.0%) (Figure 3). Only 1 patient was classified as a relapse between day 5 and Day 14: a patient in the caspofungin group had documented eradication at day 5 but then had breakthrough candidemia on days 9 and 11.

**Figure 4.**
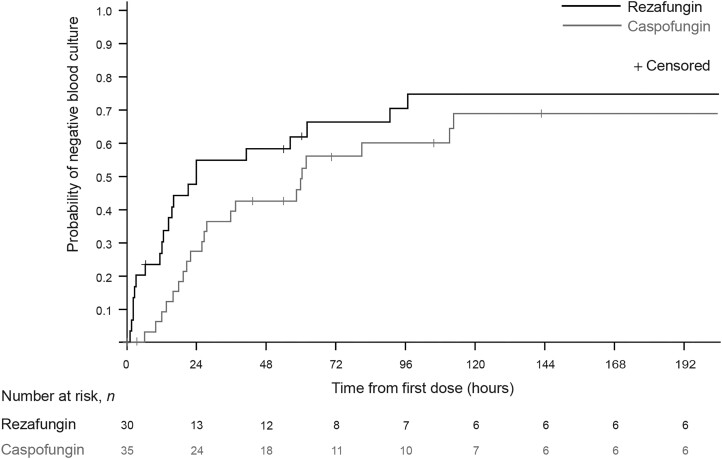
Time to negative blood culture in patients treated with rezafungin (400 mg/200 mg) or caspofungin (70 mg/50 mg) (mITT2 population). Patients with either (1) a positive blood culture drawn between 12 hours before and 72 hours after randomization or (2) a positive culture from another normally sterile site sampled between 48 hours before and 72 hours after randomization and who received ≥1 dose of the study drug. Abbreviation: mITT2, modified intention-to-treat 2.

Median TTNBC was shorter in the rezafungin versus caspofungin group (23.9 vs 60.5 hours, respectively; *P* = .094 [nominal]) (Table 2, Figure 4). At 24 hours following treatment initiation, NBCs were observed in 55.2% (16/29) and 27.3% (9/33) of patients in the rezafungin and caspofungin groups (*P* = .0162 [nominal]), respectively; at 48 hours, NBCs were observed in 58.6% (17/29) and 43.8% (14/32) of patients (*P* = .2460 [nominal]).

**Table 2.** Proportion of Patients With Negative Blood Culture After the First Dose of the Study Drug and Time From the First Dose of the Study Drug to Negative Blood Culture

	Rezafungin (400/200 mg) (N = 30)	Caspofungin (70/50 mg) (N = 35)
Patients with NBC,^a^ n (%)	26/30 (86.7)	24/35 (68.6)
Patients censored,^b^ n (%)	4/30 (13.3)	11/35 (31.4)
TTNBC,^c^ h		
Median (95% CI)	23.9 (12.3, 90.3)	60.5 (27.0, 112.6)
*P* value^c^	.094	
Patients with NBC,^a^ n (%)		
At 24 h	16/29 (55.2)	9/33 (27.3)
Patients censored^b^	1 (3.3)	2 (6.0)
*P* value^d^	.0162	
At 48 h	17/29 (58.6)	14/32 (43.8)
Patients censored^b^	1 (3.4)	3 (5.7)
*P* value^d^	.2460	

Only patients with a positive blood culture close to randomization and who received ≥1 dose of the study drug were included in this analysis.

Abbreviations: CI, confidence interval; NBC, negative blood culture; TTNBC, time to first negative blood culture.

^a^Without subsequent positive culture.

^b^Patients were censored if they received an alternative antifungal (ie, other than the study drug) for the treatment of the candidemia, died, or were lost to follow-up prior to having the negative blood culture.

^c^Nominal, from log-rank test.

^d^Nominal, from chi-squared test.

### Safety

Most patients had 1 or more TEAE (92.1% [35/38] and 84.8% [39/46] in the rezafungin and caspofungin groups, respectively) (Supplementary Table 1). Drug-related TEAEs were reported for 5 (13.2%) and 3 (6.5%) patients in the rezafungin and caspofungin groups, respectively. More than half of patients had at least 1 serious TEAE (55.3% [21/38] in the rezafungin group and 54.3% [25/46] in the caspofungin group). One patient in the rezafungin group had a serious drug-related TEAE (infusion-related reaction), whereas 2 serious drug-related TEAEs were reported in the caspofungin group (elevated transaminase levels and anaphylactic shock). The SAE reported in the rezafungin group occurred during the day 3 placebo infusion and was therefore unlikely to be related to rezafungin. A small number of patients experienced abnormal clinical laboratory evaluations (6 [15.8%] in the rezafungin group and 6 [13.0%] in the caspofungin group).

## DISCUSSION

In this preplanned analysis of the phase 3 ReSTORE study, we compared once-weekly rezafungin with once-daily caspofungin (followed by optional fluconazole) for the treatment of candidemia and/or IC in patients with a positive culture close to randomization. Our findings show comparable day 30 ACM and day 14 global response between rezafungin and caspofungin groups in this population. Because patients with a positive culture from a sample drawn more than 12 hours before randomization (blood) or more than 48 hours before randomization (other sites), who theoretically may have cleared their infection by the time of treatment initiation (either spontaneously or as a result of empiric therapy), were omitted from this analysis, this subpopulation may represent a more challenging patient population than the primary ReSTORE population. The safety and tolerability of rezafungin were comparable to caspofungin in this analysis and were consistent with previously published findings from ReSTORE [17] and for first-generation echinocandins [18, 20].

The efficacy of rezafungin versus caspofungin for the treatment of candidemia and/or IC has been previously reported in the STRIVE and ReSTORE trials [16, 17, 21]. Numerically lower day 30 ACM rates and numerically higher day 14 overall cure rates were observed in patients in STRIVE who were treated with rezafungin versus caspofungin (4% vs 13% and 76% vs 67%, respectively) [16]. Primary data for ReSTORE demonstrated noninferiority of rezafungin versus caspofungin for day 30 ACM (24% vs 21%; treatment difference 2.4% [95% CI: −9.7%, 14.4%]) and day 14 global cure (59% vs 61%; weighted treatment difference −1.1% [95% CI: −14.9%, 12.7%]) [17]. This subgroup analysis adds to these prior results reporting the efficacy of rezafungin in the treatment of candidemia and/or IC.

The differentiated stability and pharmacokinetic profile of rezafungin compared with other echinocandins supports a front-loaded, once-weekly dosing regimen [13, 22]. Front-loaded dosing may maximize the drug effect of rezafungin, as a result of its low clearance and long half-life, and the concentration-dependent activity of echinocandins [13, 22, 23]. Additionally, the high plasma concentrations afforded by the front-loaded administration of rezafungin may explain the higher concentrations of rezafungin versus micafungin seen in a murine intra-abdominal abscess model using humanized therapeutic doses of both echinocandins [24]. High rezafungin concentrations within the abscess were observed earlier and remained higher than micafungin concentrations even after 48 hours. The estimated concentration of rezafungin within the abscess also exceeded the mutant prevention concentration [14], thereby potentially reducing the risk of resistant mutant selection. The once-weekly dosing regimen has potential advantages versus daily dosing with first-generation echinocandins, such as fewer infusions, reduced need for peripherally inserted central catheter placement, fewer catheter-associated infections, lower costs, and increased compliance; lower costs and increased compliance may have particular relevance for patients needing long treatment courses [15, 17, 25, 26].

Early efficacy findings from this analysis, and those from the primary and pooled analyses of STRIVE and ReSTORE [16, 17, 21], are consistent with a potential clinical benefit of the front-loaded rezafungin dosing and resultant plasma exposure. Clearance of candidemia was more rapid in patients treated with rezafungin when compared with caspofungin: TTNBC was numerically shorter and greater proportions of patients had NBCs at both 24 and 48 hours in the rezafungin group versus the caspofungin group. Additionally, a higher proportion of patients had mycological eradication at day 5 in the rezafungin group versus the caspofungin group. This may have important implications because early eradication of candidemia may avoid dissemination to distant sites and/or reduce the likelihood of the emergence of resistance.

Differences between rezafungin and caspofungin in early eradication were more pronounced in the present subgroup analysis of patients with a positive culture close to randomization compared with the primary analysis of ReSTORE [17]; median TTNBC was 23.9 hours versus 60.5 hours (*P* = .094; subgroup analysis) and 23.9 hours versus 27.0 hours (*P* = .18; primary analysis) for rezafungin versus caspofungin groups, respectively. Likewise, NBC at 24 hours was observed in 55.2% versus 27.3% (subgroup analysis) and 53.7% versus 46.2% (primary analysis) of patients receiving rezafungin versus caspofungin, respectively [17].

Echinocandins are the recommended first-line treatment for candidemia and IC [6, 7], based on trials that demonstrated their efficacy and improved clinical outcomes versus other antifungal drugs [18, 19, 27]. However, several studies have suggested that first-generation echinocandins may be underdosed [28], especially in critically ill patients [29]. The improved pharmacokinetic profile of rezafungin [11] may explain why the early treatment benefits versus caspofungin were more pronounced in these patients who had a positive *Candida* culture closer to the time of randomization, and hence potentially had more serious infections, when compared with the overall ReSTORE population [17].

The optimal timing of antifungal treatment remains a challenge in the management of patients with candidemia/IC; early and appropriate initiation of antifungal therapy has been demonstrated to impact final outcomes in patients with candidemia and IC, yet treatment is frequently initiated too late [30–34]. Furthermore, diagnostic challenges pose an obstacle to early and appropriate treatment initiation [6]. The results of this analysis focusing on patients with a positive *Candida* culture closer to randomization therefore provide further useful insight to guide and support prompt initiation of candidemia/IC treatment.

Limitations of the analysis include the small sample size of the subgroup population, which means that these findings should be interpreted with caution and that further investigation is needed. Nonetheless, the results presented here are from a preplanned analysis of a randomized trial.

## Conclusions

This preplanned subgroup analysis supports previously described findings from the ReSTORE trial and demonstrates the efficacy and safety of weekly rezafungin for the treatment of candidemia and/or IC in patients with a positive culture close to randomization, who may represent patients with more serious infections. ACM at day 30, global cure at day 14, and mycological eradication at day 14 remained comparable between the rezafungin and caspofungin groups, with potential early clinical effects, such as the more rapid clearance of *Candida* infection, associated with front-loaded rezafungin exposure.

## Supplementary Data

Supplementary materials are available at *Clinical Infectious Diseases* online. Consisting of data provided by the authors to benefit the reader, the posted materials are not copyedited and are the sole responsibility of the authors, so questions or comments should be addressed to the corresponding author.

## Supplementary Material

ciae363_Supplementary_Data

## Notes

***Author contributions*.** A. S., H. D., and C. D. were involved in data collection and data analysis or interpretation; O. A. C., B. J. K., and J. A. V. were involved in study design and data analysis or interpretation; G. R. T. was involved in study design, data collection, and data analysis or interpretation; P. M. H., M. C., M. B., H. H., Y. K. K., M. K., N. M., P. P., T. S., and J. P. were involved in data analysis or interpretation. All authors were involved in drafting the work/reviewing it critically for important intellectual content, provided final approval of the version to be published, and agree to be accountable for all aspects of the work.

***Acknowledgments*.** The authors thank all patients and investigators involved in the study (as well as the members of the independent Data Review Committee). Medical writing support, including development of a draft outline and subsequent drafts in consultation with the authors, collating author comments, copyediting, fact checking, and referencing, was provided by Caroline Greenwood, BSc (Hons), CMPP, at Aspire Scientific Limited (Bollington, UK). Funding for medical writing support for this article was provided by Mundipharma (Cambridge, UK).

***Disclaimer*.** Employees of Cidara Therapeutics were involved in the study design. Employees of Cidara Therapeutics and Mundipharma were involved in the collection, analysis, and interpretation of data, and in the writing of the report. All authors were provided full access to all study data and had the final responsibility for the decision to submit the publication.

***Data availability*.** The study protocol is provided in the Supplementary Appendix. Access to anonymized data can be requested by contacting Enquiries@napp.co.uk. Each request will be reviewed by the sponsor for scientific merit.

***Financial support*.** The ReSTORE study was co-funded by Cidara Therapeutics and Mundipharma. Rezafungin is being developed by Cidara Therapeutics (San Diego, CA, USA) in partnership with Mundipharma (Cambridge, UK). Melinta Therapeutics (Parsippany, NJ, USA) holds the license to commercialize rezafungin in the United States. The analysis described in this manuscript was funded by Mundipharma.
