# Supplementary material for: Treatment Outcomes Among Patients With a Positive Candida Culture Close to Randomization Receiving Rezafungin or Caspofungin in the ReSTORE Study
Source: Clin Infect Dis. 2024 Jul 10;79(3):672–81. doi: 10.1093/cid/ciae363 (PMC11426279; doi:10.1093/cid/ciae363)
Supplement: ciae363_Supplementary_Data [file ciae363_supplementary_data.pdf]

Supplemental Material

Supplemental Table S1. Safety data (mITT2 population[a])

	Rezafungin (400/200 mg) (*N*=38)	Caspofungin (70/50 mg) (*N*=46)
Patients with ≥1 TEAE, *n* (%)	35 (92.1)	39 (84.8)
Patients with ≥1 drug-related TEAE, *n* (%)	5 (13.2)	3 (6.5)
Patients with ≥1 drug-related TEAE (preferred term), *n* (%)		
Abdominal pain, upper	1 (2.6)	0
Anaphylactic shock	0	1 (2.2)
Co-ordination abnormal	0	1 (2.2)
Erythema	1 (2.6)	0
Hyperphosphatemia	1 (2.6)	0
Hypertransaminasemia	0	1 (2.2)
Hypomagnesemia	1 (2.6)	0
Infusion-related reaction	1 (2.6)	0

Nystagmus	0	1 (2.2)
Tremor	1 (2.6)	0
Patients with ≥1 serious TEAE, *n* (%)	21 (55.3)	25 (54.3)
Patients with ≥1 serious drug-related TEAE (preferred term), *n* (%)	1 (2.6)	2 (4.3)
Infusion-related reaction	1 (2.6)	0
Anaphylactic shock	0	1 (2.2)
Elevated transaminase levels	0	1 (2.2)

[a]Patients who had either: (1) a positive culture from blood drawn between 12 hours before and 72 hours after randomization; or (2) a positive culture from another normally sterile site sampled between 48 hours before and 72 hours after randomization. All patients must have received at least one dose of study drug.

mITT2, modified intention-to-treat 2; TEAE, treatment-emergent adverse event.

PROTOCOL TITLE PAGE

**Title**	A Phase 3, Multicenter, Randomized, Double-blind Study of the Efficacy and Safety of Rezafungin for Injection versus Intravenous Caspofungin Followed by Optional Oral Fluconazole Step-down in the Treatment of Subjects with Candidemia and/or Invasive Candidiasis (The ReSTORE Study)
**Study Drug**	Rezafungin for Injection
**Original Protocol**	14JUNE2018
**Protocol Amendment 1**	03AUG2018
**Protocol Amendment 2**	27AUG2018
**Protocol Amendment 3**	06NOV2018
**Protocol Amendment 4**	25APR2019
**Protocol Amendment 4-FR**	26NOV2019
**Protocol Amendment 4-DE**	10FEB2020
**Protocol Amendment 4.1-DE**	30APR2020
**Protocol Amendment 5**	15OCT2020
**Sponsor**	Cidara Therapeutics, Inc.<br>6310 Nancy Ridge Drive, Suite 101<br>San Diego, California 92121, USA<br>Telephone: +1.858.752.6170<br>Facsimile: +1.858.408.3509
**Sponsor Medical Monitor**	Taylor Sandison, MD MPH<br>Cidara Therapeutics, Inc.<br>6310 Nancy Ridge Drive, Suite 101<br>San Diego, California 92121, USA<br>[redacted]
**Sponsor Clinical Trial Manager**	Lorena Kanu<br>Cidara Therapeutics, Inc.<br>6310 Nancy Ridge Drive, Suite 101<br>San Diego, California 92121, USA<br>[redacted]
**EudraCT Number**	2018-002630-21

Confidentiality Statement:
This document is a confidential communication from Cidara Therapeutics, Inc. Acceptance of this document constitutes an agreement by the recipient(s) that no information contained herein will be published or disclosed without prior written approval from Cidara Therapeutics, Inc., except that this document may be disclosed to appropriate Institutional Review Boards and/or Ethics Committees under the condition that they are also required to maintain confidentiality.

PROTOCOL APPROVAL PAGE

Protocol: CD101.IV.3.05 A5

A Phase 3, Multicenter, Randomized, Double-blind Study of the Efficacy and Safety of Rezafungin for Injection versus Intravenous Caspofungin Followed by Optional Oral Fluconazole Step-down in the Treatment of Subjects with Candidemia and/or Invasive Candidiasis (The ReSTORE Study)

Original Protocol: 14JUN2018

Protocol Amendment 1: 03AUG2018

Protocol Amendment 2: 27AUG2018

Protocol Amendment 3: 06NOV2018

Protocol Amendment 4: 25APR2019

Protocol Amendment 4-FR: 26NOV2019

Protocol Amendment 4-DE: 10FEB2020

Protocol Amendment 4.1-DE: 30APR2020

Protocol Amendment 5: 15OCT2020

SPONSOR SIGNATURE

[redacted]

Signature Date

Taylor Sandison, MD MPH
Chief Medical Officer
Cidara Therapeutics, Inc.

1.0 PROTOCOL SYNOPSIS

**Sponsor:**	Cidara Therapeutics, Inc., San Diego, California, USA
**Product Name:**	Rezafungin for Injection
**Active Ingredients:**	rezafungin acetate

Protocol Title: A Phase 3, Multicenter, Randomized, Double-blind Study of the Efficacy and Safety of Rezafungin for Injection versus Intravenous Caspofungin Followed by Optional Oral Fluconazole Step-down in the Treatment of Subjects with Candidemia and/or Invasive Candidiasis (The ReSTORE Study)

Phase: 3

Planned Study Centers: Approximately 120 clinical sites globally.

Study Duration: Maximum study duration for an individual subject is 63 days, including Screening (≤4 days, 96 hours), the Required Treatment Period (Day 1 to Day 14 [14 days]), the Optional Extended Treatment Period (Day 15 to Day 28), and Follow-up (Days 52–59).

Objectives:

The primary objectives of this study are to:

- Demonstrate that Rezafungin for Injection is non-inferior to caspofungin for all-cause mortality (ACM) at Day 30 (-2 days) in the modified intent-to-treat (mITT) population (United States Food and Drug Administration [FDA] primary objective)
- Demonstrate that Rezafungin for Injection is non-inferior to caspofungin for global cure (clinical cure as assessed by the Investigator, radiological cure [for qualifying invasive candidiasis subjects], and mycological eradication, as confirmed by the Data Review Committee [DRC]) at Day 14 (±1 day) in the mITT population (European Medicines Agency [EMA] primary objective)

The secondary objectives of this study are to:

- Compare global cure (clinical cure as assessed by the Investigator, radiological cure [for qualifying invasive candidiasis subjects], and mycological eradication, as confirmed by the DRC) for subjects receiving Rezafungin for Injection and caspofungin at Day 5, Day 30 (-2 days), End of Treatment (EOT) (≤2 days of last dose), and Follow-up (Days 52–59) in the mITT population
- Compare mycological eradication for subjects receiving Rezafungin for Injection and caspofungin at Day 5, Day 14 (±1 day), Day 30 (-2 days), EOT (≤2 days of last dose), and Follow-up (Days 52–59) in the mITT population
- Compare clinical cure as assessed by the Investigator for subjects receiving Rezafungin for Injection and caspofungin at Day 5, Day 14 (±1 day), Day 30 (-2 days), EOT (≤2 days of last dose), and Follow-up (Days 52–59) in the mITT population
- Compare radiological cure for invasive candidiasis subjects receiving Rezafungin for Injection and caspofungin at Day 5, Day 14 (±1 day), Day 30 (-2 days), EOT (≤2 days of last dose), and Follow-up (Days 52–59) in the mITT population
- Compare the safety and tolerability for subjects receiving Rezafungin for Injection and caspofungin in the safety population
- Evaluate the pharmacokinetics (PK) of Rezafungin for Injection

An exploratory objective of this study is to:

- Compare resolution of systemic signs attributable to candidemia and/or invasive candidiasis for subjects receiving Rezafungin for Injection and caspofungin at Day 5, Day 14 (±1 day), Day 30 (-2 days), and Follow-up (Days 52–59) in the mITT population

Study Design and Methodology:

The study design figure is provided in Figure 1. The Schedule of Assessments and Procedures for the Required Treatment, EOT, and Follow-up Periods is presented in Table 1 and the Schedule of Assessments and Procedures for the Optional Treatment Period is presented in Table 2.

This is a Phase 3, multicenter, prospective, randomized, double-blind, efficacy and safety study of Rezafungin for Injection (intravenous [IV]) versus an active comparator regimen of caspofungin (IV) followed by optional oral fluconazole step-down therapy in subjects with candidemia and/or invasive candidiasis. Subjects will be randomly assigned (1:1 ratio) to receive either Rezafungin for Injection or caspofungin. After ≥3 days IV treatment (or the minimum duration of IV therapy advised by the site's national/regional/local guidelines, whichever is greater), subjects may be switched to oral step-down therapy by the Investigator if they meet the oral step-down therapy criteria.

Challenges in Conducting a Pivotal Phase 3 Study During the COVID-19 Pandemic:

Study participants will be enrolled in the ReSTORE study while hospitalized and receiving strict infection control practices to prevent nosocomial transmission of SARS-CoV-2. Once study participants are discharged from the hospital, they will be followed weekly as is the standard of care and according to the protocol. Study participants will be instructed to follow infection control practices such as physical distancing, frequent hand washing, and wearing face masks. This infection control practice will also be recommended to caregivers and all who reside with the study participant. If a study participant develops signs or symptoms that suggests COVID-19 infection, we encourage them to be evaluated by their physician and have a diagnostic workup and management plan advised by the site's national/regional/local guidelines for the management of COVID-19 infection. If a subject contracts COVID-19 during the study, the diagnosis will be captured as an adverse event and the resulting safety parameters and treatment will be captured in case report forms.

Subjects Randomized to Rezafungin for Injection

Subjects randomized to Rezafungin for Injection will receive a 400 mg loading dose in Week 1, followed by 200 mg once weekly, for a total of 2 to 4 doses. To maintain the blind, subjects will also receive daily placebo to match for caspofungin IV, which will be administered on Days 2–7, Days 9–14 and during the optional dosing period on Days 16–21 and Days 23–28, or will receive daily placebo for oral step-down therapy (first eligibility on Day 4 or later as advised by a site's national/regional/local guidelines), which will be administered every day including rezafungin infusion days (see Figure 1 and Figure 2).

Subjects Randomized to Caspofungin

Subjects randomized to caspofungin will receive a total treatment of ≥14 days beginning with a single caspofungin 70 mg IV loading dose on Day 1 followed by caspofungin 50 mg IV once daily with the option to continue treatment ≤28 days. After ≥3 days (or the minimum duration of IV therapy advised by the site's national/regional/local guidelines, whichever is greater) of caspofungin treatment, subjects who meet the step-down therapy eligibility criteria (see Section 8.2.3.3) may be switched to oral fluconazole at a dose of 6 mg/kg administered once daily (rounded to the nearest 200 mg increment) with a maximum daily dose of 800 mg (e.g., a subject weighing 73 kg would receive fluconazole 400 mg dose [2 capsules of 200 mg each] based on a 6 mg/kg target dose [73 kg × 6 mg/kg = 438 mg]. NOTE: Refer to dosing section for alternative regimens of fluconazole in subjects with renal impairment in Section 8.2.3.5). To maintain the blind, subjects in the caspofungin treatment group who have switched to oral step-down therapy will receive matching IV placebo for Rezafungin for Injection once weekly on the days of scheduled Rezafungin for Injection doses until study drug is stopped (Day 8, Day 15 [if applicable] and Day 22 [if applicable]) (see Figure 1 and Figure 2).

End of Treatment Visit and Follow-up Visits

Subjects will complete an EOT visit within 2 calendar days after the last dose of study drug, and a Follow-up visit within the Day 52–59 period. Subjects who stop study drug prior to Day 22 AND are considered clinical failures (i.e., require a change in antifungal therapy to treat candidemia and/or invasive candidiasis) may have their Follow-up visit earlier, but the visit must still be ≥30 days following the last once weekly infusion of Rezafungin for Injection/placebo (i.e., Day 1, Day 8, Day 15, or Day 22).

Mycological Diagnosis

Mycological diagnosis of candidemia and/or invasive candidiasis sufficient for inclusion in the study will be established by one of the following test results from a blood or normally sterile site sample collected ≤4 days (96 hours) before randomization:

- ≥1 blood culture positive for yeast or *Candida*
- sponsor-approved rapid in vitro diagnostic (IVD) (e.g., T2Candida Panel, Septifast) test positive for *Candida* species (spp.)
- a positive gram stain (or other method of direct microscopy) for yeast or positive culture for *Candida* spp. from a specimen obtained from a normally sterile site

Note: If the positive blood culture used to qualify the subject for the study is drawn >12 hours prior to randomization, an additional set of blood cultures must be obtained ≤12 hours before randomization to determine which subjects are still candidemic at enrollment. Results from the blood cultures obtained ≤12 hours prior to randomization are not required to proceed with randomization.

The dose and duration of any prior antifungal treatment received ≤4 weeks before randomization will be recorded at Screening.

For diagnosis of invasive candidiasis, cultures should be obtained from purulent fluid or necrotic tissues from normally sterile sites. Drainage tubes following invasive procedures may only be used to acquire fluid for culture for up to 24 hours following placement of the tube (after 24 hours tubes could be colonized with non-infecting pathogens). While potentially indicative of disease, a urine culture may not be used as the qualifying culture for entry into the study, though the presence of a positive urine culture is not a reason to exclude a patient from participation. Additionally, a respiratory tract specimen would not be considered sufficient to prove invasive candidiasis as these specimens are rarely sterile and pulmonary infection with *Candida* spp. is extremely rare.

Every reasonable effort must be made for source control of the *Candida* infection to properly evaluate the efficacy of study drug. Central catheters should be removed within 48 hours after diagnosis with candidemia, consistent with Infectious Diseases Society of America (IDSA) and European Society of Clinical Microbiology and Infectious Diseases (ESCMID) guidelines. For subjects with invasive candidiasis, surgical drainage or debridement of potentially infected fluid collections or necrotic tissues should be performed.

Retinal Examination

A retinal examination for evidence of a *Candida* eye infection, including endophthalmitis or chorioretinitis, should be performed on all subjects with candidemia during Screening when possible, although the exam may occur as late as Day 7. Retinal examination is standard of care for subjects with candidemia and should be performed by an ophthalmologist, when available. The retinal examination should be repeated in subjects who were negative at baseline if the subject develops visual signs or symptoms of a *Candida* eye infection during the study. Additionally, any neutropenic subjects who recover their neutrophil counts during the study should have an additional retinal examination. Subjects who were negative at baseline and diagnosed with a *Candida* eye infection after initiating study drug must discontinue study drug but may continue in the study at the Investigator's discretion and all subsequent antifungal drugs, interventions, and outcomes for endophthalmitis or chorioretinitis will be recorded.

Study Day Definition

Study Day 1 is defined as the first day of study drug administration. Subsequent study days are counted as the number of consecutive calendar days thereafter.

Efficacy Assessments

Efficacy assessments for all subjects occur on Day 5, Day 14 (±1 day), Day 30 (-2 days), EOT (≤2 days of last dose), and the Follow-up visit (Days 52–59). Clinical cure, radiological cure (for qualifying invasive candidiasis subjects), mycological eradication, will be assessed on all efficacy assessment days. Survival status will be assessed at Day 30 (-2 days) and the Follow-up visit (Days 52–59). Subjects who complete Day 30 assessments on Day 28 or Day 29 (within the -2 days period) without further information thereafter will be considered alive at Day 30. For mycological response, the location of *Candida* infection determines how eradication will be demonstrated:

a. **Subjects with ≥1 blood culture positive for *Candida***

Blood cultures for efficacy following the first dose of study drug will be performed until the first negative blood culture result for *Candida* spp. with no subsequent positive culture (in cases when one or more samples are drawn and cultured after the first negative culture is available). Blood samples for cultures should be drawn daily when possible although may be drawn every other day until blood cultures are negative. Generally, blood cultures should involve samples from 2 separate draws with ≥1 draw from a peripheral vein without an IV catheter.

b. **Subjects with a positive culture for *Candida* spp. from a normally sterile site**

Note: only applies when infected sites are accessible; fluid samples from drainage tubes may only be collected for up to 24 hours following placement of the tube. Culture specimens for efficacy following the first dose of study drug should be collected from all normally sterile sites of baseline infection at least every other day until the first negative culture result for *Candida* spp. with no subsequent positive culture (in cases when one or more samples are drawn and cultured after the first negative culture is available). If access to the infected site is not possible, then mycological eradication will be determined based on clinical outcome (see Table 8).

Safety Assessments

Safety monitoring data is collected throughout the study. Adverse events (AEs) are collected from the time the informed consent document is signed through the Follow-up visit (Days 52–59). Vital signs will be measured at each study visit. A physical examination will be performed at the Screening, Day 5, Day 14 (±1 day), Day 30 (-2 days), EOT (≤2 days of last dose), and Follow-up (Days 52–59) visits. Subjects will be assessed for signs and symptoms of tremor, ataxia, and peripheral neuropathy using a neurological exam at least at Screening and at EOT. More frequent neurological exams may be performed as directed by subject symptoms. Hematology and chemistry laboratory tests will be performed at the Screening, Day 1, Day 2, Day 4, Day 8 (±1 day), Day 14 (±1 day), Day 30 (-2 days), EOT (≤2 days of last dose), and Follow-up visits (Days 52–59). Urinalysis will be performed at the Screening and EOT (≤2 days of last dose) visits. Coagulation laboratory tests (Prothrombin Time or International Normalized Ratio [PT/INR]) will be performed at the Screening visit. An electrocardiogram (ECG) will be performed at the Screening visit and on Day 1 at any time following administration of study drug (i.e., post-end IV infusion). An independent external Data and Safety Monitoring Board (DSMB) will review safety data when approximately 50% of subjects are randomized and as required for adverse events of special interest (AESIs) as provided in the DSMB Charter.

PK Sampling

Blood samples for PK analysis must be collected from the OPPOSITE arm of the infusion or through an arterial line during the infusion; after the infusion is complete, opposite arm or arterial line draws are preferred, but not mandatory. Blood samples will be collected as follows:

- Day 1: within 10 minutes before the end of infusion, and one sample taken any time between end of infusion and 12 hours after the end of infusion
- Day 2, 3, 4, or 5: One sample taken at any time on one day only
- Day 8: Pre-dose
- Day 14 (for subjects not receiving a Day 15 dose): One sample taken at any time
- Day 15: Pre-dose (if applicable)
- Day 22: Pre-dose (if applicable)

For subjects completing treatment on Day 14 (±1 day), a PK sample should be drawn at the same time as hematology and chemistry blood draws (i.e., random PK draw). Note: Pre-dose samples should be collected ≤30 minutes prior to the start of the infusion. For subjects receiving hemodialysis, an attempt should be made to obtain PK samples directly following hemodialysis if still within the required time window.

Blood samples will be collected from all subjects receiving study drug, although only PK samples from subjects receiving Rezafungin for Injection will be analyzed. Pharmacokinetic sample analysis will be performed by an independent, central bioanalytical laboratory using a validated assay.

Number of Subjects:

Approximately 218 subjects will be randomized to treatment (1:1, Rezafungin for Injection to caspofungin). The target of 218 subjects assumes approximately 85% of randomized subjects will be evaluable for the mITT

population. Thus, 184 subjects will be in the mITT population (92 subjects in each treatment group). If the evaluability rate for the mITT population is higher than 85%, enrollment will be stopped when at least 184 randomized subjects have met the criteria to be included in the mITT population.

Criteria for Inclusion in the Study:

Inclusion Criteria

Subjects must meet ALL the following inclusion criteria to qualify for the study:

1. Willing and able to provide written informed consent. If the subject is unable to consent for himself/herself, a legally acceptable representative (i.e., acceptable to International Council on Harmonisation [ICH] and local law, as applicable) must provide informed consent on his/her behalf.
2. Males or females ≥18 years of age.
3. Established mycological diagnosis of candidemia and/or invasive candidiasis from a sample taken ≤4 days (96 hours) before randomization defined as:
    a. ≥1 blood culture positive for yeast or *Candida*
    OR
    b. Positive test for *Candida* from a Sponsor-approved rapid IVD
    OR
    c. Positive gram stain (or other method of direct microscopy) for yeast or positive culture for *Candida* spp. from a specimen obtained from a normally sterile site.
4. Presence of one or more systemic signs attributable to candidemia or invasive candidiasis (e.g., fever, hypothermia, hypotension, tachycardia, tachypnea, local signs of inflammation) appearing from ≤12 hours prior to the qualifying positive culture through time of randomization.
5. Willing to initiate or continue medical treatment to cure infections, including receipt of antibiotics and surgical procedures, if required. Patients receiving only medications and measures for comfort and not cure should not be enrolled.
6. Female subjects of childbearing potential (all female subjects between 18 years and <2 years post-menopausal unless surgically sterile) must agree to and comply with using one barrier method (e.g., female condom with spermicide) plus one other highly effective method of birth control (e.g., oral contraceptive, implant, injectable, indwelling intrauterine device, vasectomized partner), or sexual abstinence (only possible if it corresponds to the subject's usual lifestyle) while participating in this study and at least 30 days from the last dose of study drug. Male subjects must be vasectomized, abstain from sexual intercourse, or agree to use barrier contraception (condom with spermicide), and also agree not to donate sperm while participating in this study and for 90 days thereafter (and at least 120 days from the last dose of study drug).
7. For candidemia only subjects, drawing of a set of blood cultures within 12 hours prior to randomization in the study. The result of these blood cultures is not required for inclusion in the study.

Exclusion Criteria

Subjects must NOT meet any of the following exclusion criteria to qualify for the study:

1. Any of the following forms of invasive candidiasis at baseline:
    a. Septic arthritis in a prosthetic joint (septic arthritis in a native joint is allowed)
    b. Osteomyelitis
    c. Endocarditis or myocarditis
    d. Meningitis, endophthalmitis, chorioretinitis, or any central nervous system infection
    e. Chronic disseminated candidiasis
    f. Urinary tract candidiasis due to ascending *Candida* infection secondary to obstruction or surgical instrumentation of the urinary tract
2. Received systemic treatment with an antifungal agent at approved doses for treatment of candidemia for >48 hours (e.g., >2 doses of a once daily antifungal agent or >4 doses of a twice daily antifungal agent) and ≤4 days (96 hours) before randomization.
    a. Exception: Receipt of antifungal therapy to which any *Candida* spp. isolated in culture is not susceptible (see Appendix 1)
3. Alanine aminotransferase or aspartate aminotransferase levels >10-fold the upper limit of normal (ULN).

4. Severe hepatic impairment in subjects with a history of chronic cirrhosis (Child-Pugh score >9).
5. Presence of an indwelling vascular catheter or device that cannot be removed or an abscess that cannot be drained and is likely to be the source of candidemia or invasive candidiasis.
6. Known hypersensitivity to Rezafungin for Injection, caspofungin, any echinocandin, or to any of their excipients, including but not limited to hereditary sugar disorders (e.g., fructose intolerance, sucrose-isomaltase insufficiency), anaphylaxis, or echinocandin-induced exfoliative skin disorders (e.g., Stevens-Johnson syndrome or toxic epidermal necrolysis).
7. Meets National Cancer Institute Common Terminology Criteria for Adverse Events (CTCAE) version 5.0, criteria for ataxia, tremor, motor neuropathy, or sensory neuropathy of Grade 2 or higher.
8. History of severe ataxia, tremor, or neuropathy or a diagnosis of multiple sclerosis or a movement disorder (including Parkinson's Disease or Huntington's Disease).
9. Planned or ongoing therapy at Screening with a known severe neurotoxic medication (see Appendix 9) or with a known moderate neurotoxic medication (see Appendix 9) in a patient with ataxia, tremor, motor neuropathy, or sensory neuropathy of CTCAE version 5.0 Grade 1 or higher.
10. Previous participation in this or any previous rezafungin study.
11. Current participation in another interventional treatment trial with an investigational agent.
12. Recent use of an investigational medicinal product within 28 days of the first dose of study drug or presence of an investigational device at the time of screening.
13. Pregnant or lactating females.
14. The Principal Investigator (PI) is of the opinion the subject should not participate in the study.

Test Product, Dose, and Mode of Administration:

Rezafungin for Injection is a lyophilized formulation of the active pharmaceutical ingredient, rezafungin acetate. Rezafungin for Injection is supplied in vials as a sterile lyophilized powder (200 mg) for reconstitution prior to dilution into normal saline infusion bags (250 mL).

Rezafungin for Injection is administered IV with a 400 mg loading dose on Day 1 of Week 1, followed by 200 mg once weekly, for a total of 2 to 4 doses.

Intravenous infusion of Rezafungin for Injection and IV placebo is administered over 60 (±10) minutes, although the infusion time may be increased up to 180 minutes to manage evolving symptoms of infusion reaction consistent with management of echinocandin class infusion reactions, whereby decreasing the rate of infusion often alleviates symptoms. Dosage adjustments of Rezafungin for Injection are not allowed. Treatment will be initiated on an inpatient basis; a switch to outpatient IV treatment is allowed at the Investigator's discretion.

The daily dosing schedule for the required treatment period is presented in Figure 2 and the optional treatment period in Figure 3. The daily dosing schedule for the entire study period (required and optional) is presented in Table 3.

Reference Therapy, Dose, and Mode of Administration:

Active Comparator Regimens

Caspofungin

Subjects randomized to caspofungin will receive a total treatment of ≥14 days beginning with a single 70 mg loading dose on Day 1, followed by 50 mg once daily with the option to continue treatment ≤28 days, unless they qualify for an alternative caspofungin regimen per Section 8.2.2.4 or for oral step-down therapy after ≥3 days (or the minimum duration of IV therapy advised by the site's national/regional/local guidelines, whichever is greater) of caspofungin IV treatment per Section 8.2.3. Doses are to be administered 24 (±2) hours after the start of study drug administration on the previous day.

Intravenous infusion of IV caspofungin is administered over 60 (±10) minutes, although the infusion time may be increased up to 180 minutes to manage evolving symptoms of infusion reaction consistent with management of echinocandin class infusion reactions, whereby decreasing the rate of infusion often alleviates symptoms.

Alternative Caspofungin Regimens and Guidance

Subjects with moderate hepatic impairment (Child-Pugh score of 7–9) will receive a loading dose of caspofungin of 70 mg on Day 1 followed by 35 mg once daily.

Subjects weighing >80 kg or on concomitant rifampin, nevirapine, efavirenz, phenytoin, dexamethasone, or carbamazepine may receive caspofungin 70 mg once daily. Dose adjustment due to drug-drug interactions (DDIs) or weight may be considered according to the approved labeling and at the Investigator's discretion.

Use of concomitant cyclosporine should be limited to subjects for whom potential benefit outweighs potential risk of DDIs and hepatotoxicity with caspofungin.

Oral Step-Down Regimen: Fluconazole

After ≥3 days of caspofungin treatment (or the minimum duration of IV therapy advised by the site's national/regional/local guidelines, whichever is greater), subjects who meet the step-down therapy eligibility criteria may be switched to oral fluconazole (earliest oral dose on Day 4 or later, as advised by a site's national/regional/local guidelines). The total comparator treatment period (caspofungin plus fluconazole) is ≤28 days. Subjects weighing over 130 kg will not be eligible for oral step-down therapy. Subjects with a creatinine clearance >50 mL/min (see Appendix 2) will receive oral fluconazole at a dose of 6 mg/kg administered once daily rounded to the nearest 200 mg increment with a maximum daily dose of 800 mg (e.g., a subject weighing 73 kg would receive a fluconazole 400 mg dose [2 capsules of 200 mg each] based on a 6 mg/kg target dose [73 kg × 6 mg/kg = 438 mg]). Subjects with creatinine clearance ≤50 mL/min will receive oral fluconazole at a dose of 3 mg/kg administered once daily rounded to the nearest 200 mg increment with a maximum daily dose of 400 mg (e.g., a subject weighing 73 kg would receive a fluconazole 200 mg dose [1 capsule of 200 mg] based on a 3 mg/kg target dose [73 kg × 3 mg/kg = 219 mg]). Subjects receiving hemodialysis receive the same dose as subjects with a creatinine clearance >50 mL/min; however, they are only dosed following hemodialysis. Subjects receiving continuous renal replacement therapy (CRRT) should be treated as a subject with normal renal function at 6 mg/kg rounded to the nearest 200 mg each day (Cousin 2003). Subjects receiving peritoneal dialysis should be treated with 1.5 mg/kg rounded to the nearest 200 mg each day (Cousin 2003). Investigators are advised to pay close attention to signs of overdosing and underdosing with underweight (<50 kg) and overweight (>85 kg) subjects, respectively. Additionally, signs of potential fluconazole toxicity, including hepatic toxicity and QT prolongation, should be closely monitored for any subject on oral step-down therapy.

Oral Step-Down Therapy Eligibility Criteria

An oral step-down therapy is allowed in both treatment groups (fluconazole for caspofungin, placebo for oral step-down therapy for Rezafungin for Injection), provided the following criteria are met:

- Able to take oral medication
- ≥3 days of IV study drug (or the minimum duration of IV therapy advised by the site's national/regional/local guidelines, whichever is greater)
- The *Candida* spp. isolated is susceptible to fluconazole
- All signs and symptoms of candidemia and/or invasive candidiasis that were present at baseline have resolved
- The subject's clinical status is considered stable based on Investigator assessment
- Most recent blood culture must be drawn following the first dose of study drug AND ≥48 hours prior to oral study drug initiation AND must be negative for *Candida* spp.
- No evidence of moderate or severe hepatic injury (alanine aminotransferase or aspartate aminotransferase >5× the ULN)
- No history of hypersensitivity to any azole or any other contraindications to the use of fluconazole or its excipients, including concomitant use of the following medications: terfenadine, cisapride, astemizole, erythromycin, pimozide, and quinidine
- No personal or family history of Long QT interval on ECG (QT) syndrome or a prolonged QT interval corrected for heart rate by Fridericia's formula (QTcF) interval (>470 msec in males and >480 msec in females)
- Investigator's opinion, the subject can tolerate oral fluconazole therapy (refer to current fluconazole Prescribing Information)
- Subject's weight is ≤130 kg

While QT prolongation is rare with fluconazole without the contraindicated interacting medications listed above, investigators should monitor subjects with ECGs as clinically indicated.

Subjects who are switched to oral step-down therapy may switch back to IV study drug therapy in the event of the development of an unforeseen condition that prevents the subject from taking oral medications (e.g., pancreatitis, urgent surgery), but may not switch back to IV study drug therapy for worsening or relapse of candidemia/invasive candidiasis or for intolerance or toxicity due to study drug.

The daily dosing schedule with options for the required treatment period is presented in Figure 2 and for the optional treatment period is in Figure 3. The daily dosing schedule for the entire study period (required and optional) is presented in Table 3.

Placebo, Dose, and Mode of Administration:

In this double-blind, double-dummy, active comparator study, two placebo regimens are required; one for subjects randomized to Rezafungin for Injection and one for subjects randomized to caspofungin. In addition, because subjects in the caspofungin arm may be switched to oral step-down therapy, an oral placebo is required for subjects in the Rezafungin for Injection arm. Thus, placebo requirements for each treatment assignment are as follows:

Treatment Assignment	Placebo
Rezafungin for Injection	Placebo for caspofungin IV
	Placebo for oral step-down therapy (fluconazole)
Caspofungin IV	Placebo for Rezafungin for Injection

In more detail, placebo regimens for each treatment assignment are as follows:

Placebo Regimen for Rezafungin for Injection Treatment Assignment

Subjects will receive a caspofungin IV equivalent regimen of daily placebo IV infusion in a blinded manner. Because subjects randomized to caspofungin may be switched to fluconazole oral step-down therapy, a fluconazole-equivalent regimen of daily oral placebo will be administered for subjects randomized to Rezafungin for Injection at that time.

Placebo Regimen for Caspofungin IV Treatment Assignment

Subjects who are switched to fluconazole oral step-down therapy will receive a Rezafungin for Injection equivalent regimen of weekly placebo IV infusion in a blinded manner.

Duration of Treatment:

The treatment period for study drug administration is ≥14 days to ≤28 days with the last required dose of study drug on Day 14 and the last possible dose of study drug on Day 28. Treatment may be stopped on any day between Day 14 and Day 28 at the discretion of the Investigator.

Known and Potential Risks

Known Risks

Infusion reactions characterized by flushing, shortness of breath, and nausea have been observed with rezafungin and are similar to infusion reactions noted with other echinocandins. Symptoms of the infusion reaction may resolve without intervention or with either slowing of the infusion rate or interruption of the infusion.

Mild photosensitivity to ultraviolet (UV) light has been demonstrated in subjects administered rezafungin in a Phase 1 clinical trial. Subjects should use measures to reduce sun and other UV light exposure while on study drug, and for 5 half-lives (approximately 30 days) after the last dose of rezafungin administered.

The Investigator's Brochure should be referenced for additional details or updated information throughout the conduct of the study.

Potential Risks

Echinocandins are typically well tolerated (Eraxis [anidulafungin] Prescribing Information; Mycamine [micafungin sodium] Prescribing Information; Cancidas [caspofungin acetate] Prescribing Information). Potential drug class effects include risk of abnormal liver function tests, hepatitis, and hepatic failure. General and administration site events (infusion related reactions, peripheral edema, rigors, infusion site inflammation,

and pyrexia) have been reported for echinocandins (Eraxis [anidulafungin] Prescribing Information; Mycamine [micafungin sodium] Prescribing Information; Cancidas [caspofungin acetate] Prescribing Information). Possible histamine-mediated symptoms have been reported in patients who received rapid infusions of echinocandins, including rash, urticaria, flushing, pruritus, dyspnea, hypotension, facial swelling, and vasodilation. Anaphylactic-like reactions have been reported with micafungin (Mycamine [micafungin sodium] Prescribing Information). While uncommon, subjects with a history of hypersensitivity to caspofungin or its excipients, including but not limited to anaphylaxis and exfoliative skin disorders, should be excluded from this study.

Based on a fertility study in male rats, there is the potential risk for decreased sperm motility, increased incidences of abnormal sperm morphology, and testicular seminiferous tubular epithelial degeneration. These findings were noted at 2.5-fold the exposure (the estimated steady state plasma level in humans at 200 mg) for Rezafungin subjects in this clinical trial, who will receive 400 mg once weekly loading dose followed by 200 mg once weekly for a total of 2 to 4 doses. The risk to humans is unknown, thus the male contraception requirements in inclusion criterion 4 prohibit unprotected sexual intercourse and sperm donation within the study period and for 90 days thereafter (and a total of 120 days from the last dose of study drug). This risk is included in the informed consent form (ICF).

In a 3-month study in monkeys, there were observations of tremors and histology consistent with Schwann cell hypertrophy/hyperplasia and demyelination in the dorsal root ganglia first appearing at week 6 of dosing. These observations occurred at 11-fold the exposure for the proposed rezafungin dosing regimen in this study. Given the late timing and high exposure relative to the rezafungin dosing regimen, the risk to study subjects is assessed as low. To collect further data on specific signs and symptoms associated with the neurologic events noted in these nonclinical studies, there will be periodic neurologic exams and follow up of neurologic adverse events of special interest (AESIs).

Because there may be unknown and potential risks with administration of Rezafungin for Injection, all subjects will be closely monitored for safety and tolerability by repeated assessment of clinical, vital signs, ECGs, and clinical laboratory safety parameters and reporting of AEs. Additionally, there are AESIs, for which investigators will be trained to monitor closely and thorough investigations will occur (see Section 12.7). Finally, an independent DSMB will review all safety parameters throughout the study.

Criteria for Evaluation:

Efficacy

The primary efficacy outcome for the FDA is ACM at Day 30 (-2 days).

The primary efficacy outcome for the EMA is global cure (based on clinical cure as assessed by the Investigator, radiological cure [for those subjects with invasive candidiasis documented by radiologic/imaging evidence at baseline] and mycological eradication) confirmed by an independent DRC at Day 14 (±1 day).

All-cause mortality at Day 30 (-2 days) is a secondary outcome for the EMA, and global cure at Day 14 (±1 day) is a secondary outcome for the FDA.

Additional secondary efficacy outcome measures include global cure (confirmed by the DRC) at Day 5, Day 30 (-2 days), EOT (≤2 days of last dose), and Follow-up (Days 52–59); mycological eradication, clinical cure, and radiological cure (for those subjects with invasive candidiasis documented by radiologic/imaging evidence at baseline) at Day 5, Day 14 (±1 day), Day 30 (-2 days), EOT (≤2 days of last dose), and Follow-up (Days 52–59). An exploratory efficacy outcome measure is resolution of attributable systemic signs of candidemia and/or invasive candidiasis that were present at baseline, Day 5, Day 14 (±1 day), Day 30 (-2 days), EOT (≤2 days of last dose), and Follow-up (Days 52–59).

Pharmacokinetics and/or Pharmacodynamics

Blood samples will be collected from subjects to evaluate the PK of Rezafungin for Injection. Blood samples will be collected from all subjects, but only PK samples from subjects receiving Rezafungin for Injection will be analyzed. Plasma samples will be analyzed for the concentration of rezafungin at a central bioanalytical laboratory using a validated liquid chromatography-tandem mass spectrometry method. Samples from this study may also be included in a population PK analysis, which will be reported separately.

In addition, rezafungin plasma protein binding and albumin concentrations will be determined from pre-dose plasma samples from subjects enrolled prior to amendment 5, and reported separately.

Safety

Safety will be assessed through the evaluation of AEs, vital signs (temperature, heart rate, blood pressure, and respiratory rate), ECGs, physical examinations (including neurological examinations), and clinical laboratory data (clinical chemistry panels, hematology evaluations, and urinalyses). Subjects will also be assessed at least at the Screening and EOT visits with a thorough neurologic evaluation to assess for signs and symptoms of tremor, ataxia, and peripheral neuropathy. Adverse events and abnormal laboratory values will be graded for severity using the National Cancer Institute CTCAE version 5.0.

Statistical Methods:

Sample Size and Stratification

For the EMA endpoint of global cure (confirmed by the DRC), using a 20% non-inferiority (NI) margin, one-sided alpha of 0.025, 80% power, 1:1 randomization, a global cure of 70% in both the Rezafungin for Injection and caspofungin groups, and the sample size methodology based on a continuity corrected Z-statistic, a total of 184 subjects (92 subjects in each treatment group) are required in the mITT population. Assuming 85% of subjects will be evaluable for the mITT population, a total of approximately 218 subjects will be randomized. For the FDA endpoint of ACM at Day 30 (-2 days) in the mITT population, using a 20% NI margin, one-sided alpha of 0.025, an ACM at Day 30 rate of 20% in both treatment groups, 1:1 randomization, and the sample size methodology based on a continuity corrected Z-statistic, a total of 184 subjects in the mITT population provides an 89.7% power to show NI.

Randomization will be stratified based on diagnosis (candidemia only; invasive candidiasis) and by Acute Physiology and Chronic Health Evaluation (APACHE II) score/absolute neutrophil count (ANC) (APACHE II score ≥20 OR ANC<500 cells/µL; APACHE II score <20 AND ANC≥500 cells/µL) at Screening.

Analysis Populations

The analysis populations are defined for this study as follows:

- The intent-to-treat (ITT) population will include all randomized subjects
- The Safety population will include all subjects who received any amount of study drug
- The mITT population will include all subjects who had documented *Candida* infection based on Central Laboratory evaluation of a culture from blood or another normally sterile site obtained ≤4 days (96 hours) before randomization and received ≥1 dose of study drug
- The clinically evaluable (CE) population will include all subjects in the mITT population who also met inclusion criterion #4, did not meet exclusion criteria #1, #2, or #5, had an assessment of both mycological and clinical response at Day 14 in the window of Day 14 (±1 day) (subjects with invasive candidiasis documented by radiologic/imaging evidence also must have an assessment of radiologic response), and did not have any other factor that could confound the assessment of the global response at Day 14
- Pharmacokinetic Analysis Population will include all Rezafungin for Injection subjects with at least 1 plasma sample obtained for PK analysis

Statistical Methodology

The primary efficacy outcome for the FDA is ACM at Day 30 (-2 days). The number and percentage of subjects in each treatment group who are alive and deceased at Day 30 will be determined in the mITT population. A two-sided 95% confidence interval (CI) for the observed difference in the ACM rate (Rezafungin for Injection group minus caspofungin group) will be calculated using the unadjusted method of Miettinen and Nurminen. If the upper bound of the 95% CI is lower than 20%, NI of Rezafungin for Injection will be concluded.

The primary efficacy outcome for the EMA is global cure (DRC confirmed) at Day 14 (±1 day) in the mITT population. The number and percentage of subjects in each treatment group who have a global response of cure, failure, or indeterminate, will be presented by treatment group at Day 14 (±1 day) in the mITT population. An adjusted (for the randomization stratification factors) two-sided 95% CI for the observed difference in the global cure rate (Rezafungin for Injection group minus caspofungin group) will be calculated using the method of Miettinen and Nurminen. If the lower bound of the 95% CI is greater than -20%, NI of Rezafungin for Injection will be concluded.

The secondary efficacy outcomes are global cure (confirmed by the DRC), mycological response, clinical response, and radiological response (for subjects with invasive candidiasis documented by radiologic/imaging

evidence at baseline). The number and percentage of subjects with a global response of cure, failure, or indeterminate will be presented by treatment group in the mITT population at Day 5, Day 30 (-2 days), EOT (≤2 days of last dose), and Follow-up (Days 52–59). The number and percentage of subjects with a mycological response of eradication, failure, or indeterminate will be presented by treatment group in the mITT population at Day 5, Day 14 (±1 day), Day 30 (-2 days), EOT (≤2 days of last dose), and Follow-up (Days 52–59). The number and percentage of subjects with a clinical response of cure, failure, or indeterminate will be presented by treatment group in the mITT population at Day 5, Day 14 (±1 day), Day 30 (-2 days), EOT (≤2 days of last dose), and Follow-up (Days 52–59). The number and percentage of subjects with invasive candidiasis documented by radiologic/imaging evidence, a radiological cure, failure, or indeterminate will be presented by treatment group in the mITT population at Day 5, Day 14 (±1 day), Day 30 (-2 days), EOT (≤2 days of last dose), and Follow-up (Days 52–59). The 95% CIs for the treatment differences in global cure, mycological eradication, clinical cure as assessed by the Investigator, and radiological cure, will be determined.

Safety will be evaluated by presenting summaries of AEs and serious adverse events (SAEs), clinical laboratory evaluations (hematology evaluation, chemistry panel, urinalysis), ECGs, and vital signs. Safety variables will be tabulated for the Safety population by treatment group. Adverse events will be coded using the Medical Dictionary for Regulatory Activities (MedDRA). A treatment-emergent adverse event (TEAE) is defined as an AE that occurs during or after study drug administration and up through the Follow-up visit (Days 52–59). The incidence of TEAEs will be presented by system organ class (SOC) and preferred term (PT), by relationship to study drug, and by CTCAE grade (version 5.0). In addition, the incidence of serious TEAEs and TEAEs leading to discontinuation of study drug will be presented by SOC and PT. Descriptive statistics for clinical laboratory test results, ECGs, and vital signs, including changes from Baseline, will be presented by time point. Incidences of potentially clinically significant (PCS) clinical laboratory results, ECGs, and vital signs, as defined in the Statistical Analysis Plan (SAP), will also be summarized by time point. The number and percentage of subjects with AEs of special interest of neuropathy and tremor will be provided by study drug group. Samples from this study may be included in a population PK analysis, which will be reported separately. In addition, rezafungin plasma protein binding and albumin concentrations will be determined from pre-dose plasma samples from subjects enrolled prior to amendment 5, and reported separately. The PK parameters that will be assessed in the PK Analysis Population include: maximum plasma concentration (C_{max}), time to C_{max} (T_{max}), and area under the concentration-time curve (AUC), if applicable.

Impact of the COVID-19 Pandemic on Analysis

The COVID-19 pandemic may impact study participants' ability to return to the clinic possibly resulting in premature discontinuation of study drug, premature discontinuation of the study, or missed study visits. Additionally, it is possible the COVID-19 pandemic may impact efficacy and safety outcomes. All missed visits due to COVID-19 will be noted as protocol deviations, and diagnoses of COVID-19 will be recorded as medical history or adverse events. Analyses to assess the possible impact of COVID-19 on the study results are described in the SAP.

Figure 1: Study Design Diagram

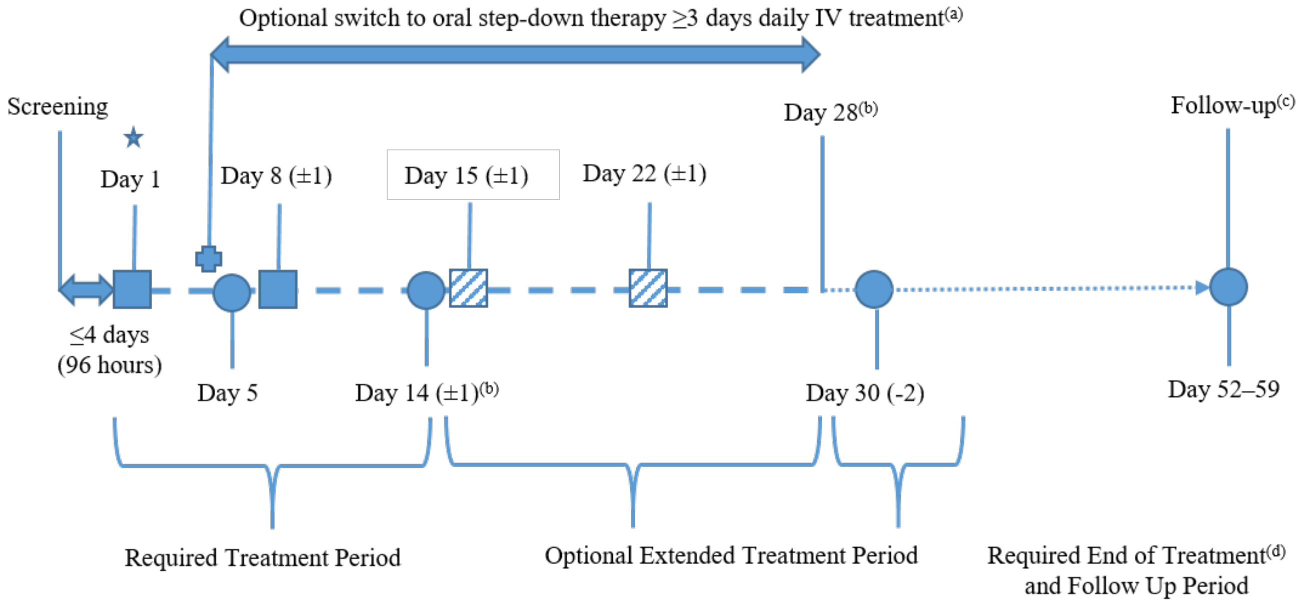

(a) After ≥3 days of IV study drug (or the minimum duration of IV therapy advised by the site's national/regional/local guidelines, whichever is greater), subjects are permitted to switch to oral step-down therapy so long as all criteria is met as specified in the protocol. Subjects who are switched to oral step-down therapy may switch back to IV study drug therapy in the event of the development of a condition that prevents the subject from taking oral medications (e.g., pancreatitis, urgent surgery), but may not switch back to IV study drug therapy for relapse of candidemia/IC or for intolerance or toxicity due to study drug.

(b) Day 14 is the last required dose of study drug and Day 28 is the last possible dose of study drug. An End of Treatment visit is required ≤2 calendar days after last dose of study drug.

(c) Follow-up will occur between Days 52–59. Subjects who stop study drug early (i.e. clinical failure) and require a change in antifungal therapy to treat candidemia and/or invasive candidiasis may have an earlier Follow-up visit occurring ≥30 days from the last weekly dose of IV rezafungin or IV placebo.

(d) Subjects will complete an End of Treatment visit ≤2 calendar days after the last dose of study drug. All safety assessments are to be completed at the End of Treatment visit. Efficacy assessments are also to be completed at the End of Treatment visit.

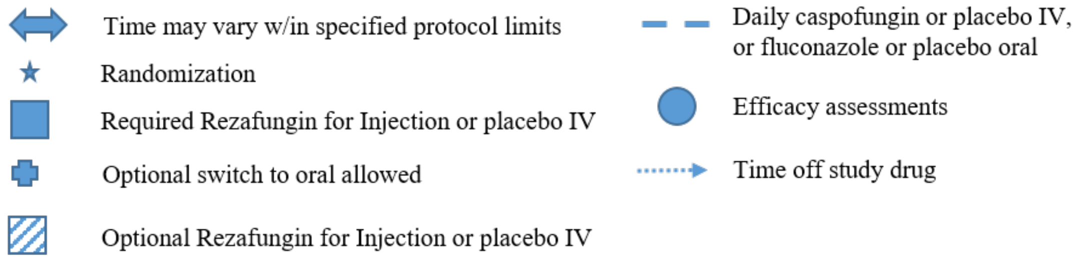

Figure 2: Dosing Schedule for Required Treatment Period

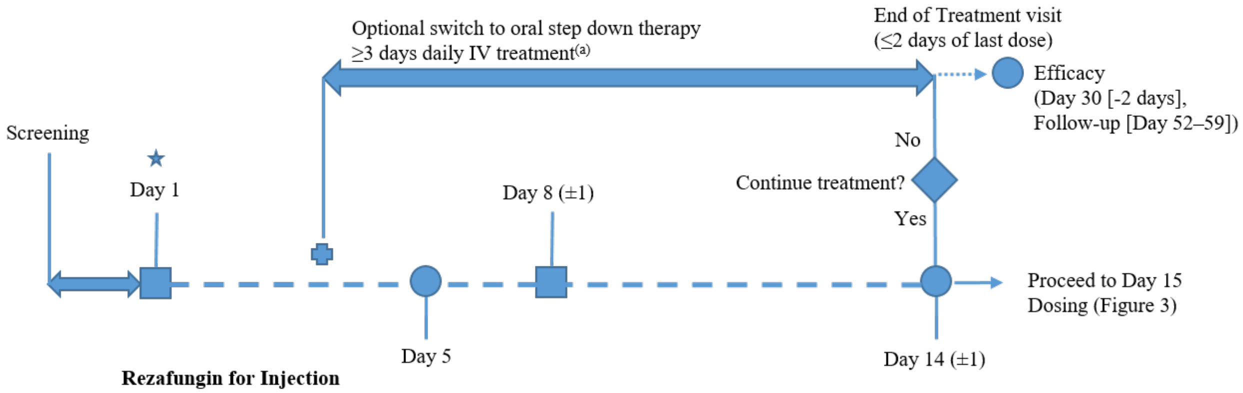

Rezafungin for Injection

Day	1	2	3	4	5	6	7	8	9	10	11	12	13	14
Rezafungin for Injection IV	X							X						
IV PBO		X	X	X	X	X	X		X	X	X	X	X	X

Bottom schedule is followed at time of switch to oral therapy (or) (or) (or) (or) (or) (or) (or) (or) (or) (or) (or)

Rezafungin for Injection IV								X						
Oral PBO				X	X	X	X	X	X	X	X	X	X	X

Caspofungin (with option for oral step-down therapy)

Day	1	2	3	4	5	6	7	8	9	10	11	12	13	14
Caspofungin IV	X	X	X	X	X	X	X	X	X	X	X	X	X	X

Bottom schedule is followed at time of switch to oral therapy (or) (or) (or) (or) (or) (or) (or) (or) (or) (or) (or)

IV PBO								X						
Fluconazole Oral				X	X	X	X	X	X	X	X	X	X	X

(a) After ≥3 days of IV study drug (or the minimum duration of IV therapy advised by the site's national/regional/local guidelines, whichever is greater), subjects are permitted to switch to oral step-down therapy so long as all criteria is met as specified in the protocol.

Figure 3: Dosing Schedule for Optional Treatment Period

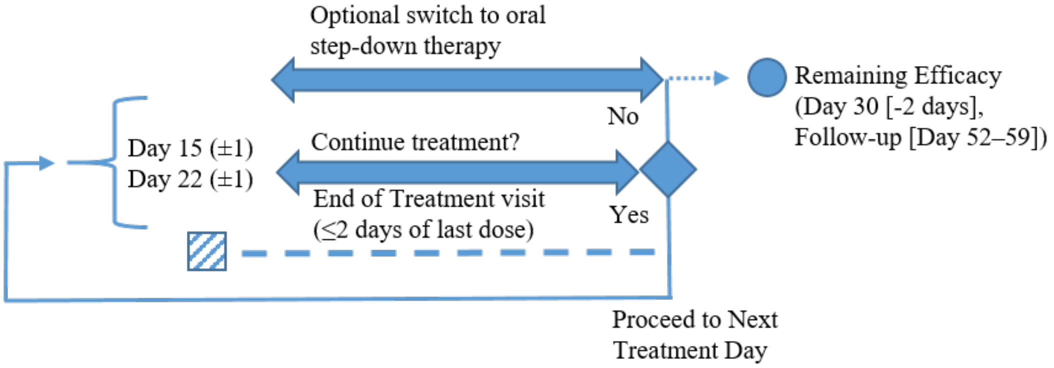

Rezafungin for Injection

	15	16	17	18	19	20	21
	22	23	24	25	26	27	28
Rezafungin for Injection IV	X						
IV PBO		X	X	X	X	X	X
	(or)	(or)	(or)	(or)	(or)	(or)	(or)

Bottom schedule is followed at time of switch to oral therapy

Rezafungin for Injection IV	X						
Oral PBO	X	X	X	X	X	X	X

Caspofungin (with option for oral step-down therapy)

	15	16	17	18	19	20	21
	22	23	24	25	26	27	28
Caspofungin IV	X	X	X	X	X	X	X
	(or)	(or)	(or)	(or)	(or)	(or)	(or)

Bottom schedule is followed at time of switch to oral therapy

IV PBO	X						
Fluconazole Oral	X	X	X	X	X	X	X

Table 1: Schedule of Assessments and Procedures for the Required Treatment, End of Treatment, and Follow-up Periods

Assessment or Procedure	Screening ≤4 days (96 hours) before randomization	Required Treatment Period (Days)								Day 30 (-2)	EOT	Follow-up [c]
		1 [a]	2	3	4	5–7	8 (±1) [b]	9–13	14 (±1) [b]	Efficacy, Safety	≤2 days after last dose (IV or oral)	Days 52–59
Informed consent [d]	X											
Medical history [e]	X											
Physical examination, including weight and height [f]	X	X [g]	X [g]	X [g]	X [g]	X	X [g]	X [g]	X	X	X	X
Neurological examination [h]	X	X [h]	X [h]	X [h]	X [h]	X [h]	X [h]	X [h]	X [h]	X [h]	X	X [h]
Vital signs [i]	X	X	X	X	X	X	X	X	X	X	X	X
Assess Central Venous Catheters [j]	X	X	X	X	X	X	X	X	X	X	X	
Blood for hematology and chemistry tests (see Appendix 3)	X	X	X		X		X		X	X	X	X
Blood for PT/INR (see Appendix 3)	X											
Calculate Child-Pugh score [k] (see Appendix 4)	X											
Calculate modified APACHE II with Glasgow coma score [l]	X											
12-lead ECG [m]	X	X										
Radiologic test results [n]	X	→	→	→	→	→	→	→	→	→	X	X
Urine for urinalyses, microscopy [o] (see Appendix 3)	X										X	
Serum pregnancy test [p]	X											X
Retinal examination for *Candida* eye infection [q]	X [q]	X [q]	X [q]	X [q]	X [q]	X [q]	X [q]	X [q]	X [q]	X [q]	X [q]	X [q]
Blood or normally sterile tissue/fluid for culture [r, s]	X [r]	X [s]	X [s]	X [s]	X [s]	X [s]	X [s]	X [s]	X [s]	X [s]	X [s]	X [s]

**Assessment or Procedure**	**Screening** ≤4 days (96 hours) before randomization	**Required Treatment Period (Days)**								**Day 30 (-2)** Efficacy, Safety	**EOT** ≤2 days after last dose (IV or oral)	**Follow-up** [c] Days 52–59
		**1** [a]	**2**	**3**	**4**	**5–7**	**8 (±1)** [b]	**9–13**	**14 (±1)** [b]			
Blood for Rapid IVD [t]	X [t]											
Study randomization [u]		X										
Administer study drug and/or placebo (See Table 3)		X	X	X	X	X	X	X	X			
Record prior and/or concomitant medications [v]	X	→	→	→	→	→	→	→	→	→	X	X
Record adverse events [w]	X	→	→	→	→	→	→	→	→	→	X	X
Assess presence or absence of systemic signs of candidemia/ invasive candidiasis	X					X [z]			X	X		X
Assess for clinical response [y]						X [z]			X	X	X	X
Assess for radiologic response [aa]						X			X	X	X	X
Assess ACM										X		X
Blood for PK testing [bb]	X [cc]	X [bb]	X [dd]	X [dd]	X [dd]	X [dd]	X [bb]		X [ee]			

Abbreviations: ACM = all-cause mortality; APACHE II = Acute Physiology and Chronic Health Evaluation II; ECG = electrocardiogram; eCRF = electronic case report form; EOT = End of Treatment; ESCMID = European Society of Clinical Microbiology and Infectious Diseases; ICF = Informed Consent Form; ICH = International Council for Harmonisation; ICU = intensive care unit; IDSA = Infectious Diseases Society of America; IV = intravenous(ly); IVD = in vitro diagnostic; PCR = polymerase chain reaction; PI = Principal Investigator; PK = pharmacokinetic; spp. = species; PT/INR= Prothrombin Time/International Normalized Ratio.

[a] Study Day 1 is defined as the first day of study drug administration; subsequent study days are counted as the number of consecutive calendar days thereafter.

[b] Windows apply to subjects who are receiving oral step-down therapy on an outpatient basis or who are inpatient and are scheduled to be discharged by the next calendar day.

[c] Follow-up is to occur on Days 52–59. Subjects who stop study drug prior to Day 22 AND are considered clinical failures (i.e., require a change in antifungal therapy to treat candidemia and/or invasive candidiasis) may have their Follow-up visit earlier but must still be ≥30 days following the last once weekly infusion of Rezafungin for Injection/placebo (i.e., Day 1, Day 8, Day 15, or Day 22).

[d] Written informed consent must be obtained prior to initiating any study related assessments or procedures. Consent from a legally acceptable representative (i.e., acceptable to ICH and local law, as applicable) may be obtained if the subject is unable to consent for themselves.

[e] Medical history for the last 5 years and *Candida* risk factors for the last 3 months (e.g., central line, active malignancy, broad-spectrum antibiotic therapy, diabetes mellitus, immunosuppression, major surgery, total parenteral nutrition, transplant recipient, trauma, dialysis, burns, pancreatitis) and ICU admission and discharge (if applicable).

[f] Height at Screening only; weight at Screening, Day 14, and Follow-up (Days 52–59); all other assessments at specified visits.

[g] A focused physical exam (including a neurological exam) should be performed as clinically indicated by adverse event screening and course of the underlying disease.

[h] As part of the physical exam, a thorough neurologic exam is to be performed at Screening and at the EOT visit. At all other time points, a focused physical exam (including a neurological exam) is to be performed as clinically indicated, driven by the subject's signs and symptoms.

[i] Temperature and the method used (oral, rectal, temporal, tympanic, or core), heart rate, blood pressure, and respiratory rate. Axillary temperatures are allowed, but not preferred.

[j] Presence of central line during Screening or placed during the study should be documented with dates of insertion and removal. Short-term central venous catheters (e.g., peripherally-inserted central catheters, internal jugular, or subclavian central venous catheters) and long-term central venous catheters (e.g., tunneled catheters) are recommended to be removed within 48 hours after diagnosis with candidemia, consistent with IDSA and ESCMID guidelines.

[k] Child-Pugh score is only required for subjects with a history of chronic cirrhosis.

[l] APACHE II score is a stratification factor for randomization and must be calculated prior to enrollment using vital signs and laboratory results from Screening. If multiple vital signs and laboratory values are obtained during the Screening period, it is preferred to use those collected most closely to the time of randomization. See Appendix 5 for APACHE II and Appendix 6 for Glasgow coma score.

[m] 12-lead ECG must be conducted prior to randomization and at any time on Day 1 following administration of study drug (i.e., post-end IV infusion).

[n] Radiologic test type with findings and interpretation should only be recorded if the test provides evidence of invasive candidiasis or evidence of progression, stabilization, improvement, or resolution of invasive candidiasis compared with previous radiographs.

[o] While potentially indicative of disease, a positive urine culture for *Candida* spp. may not be used as the qualifying culture for entry into the study, though the presence of a positive urine culture is not a reason to exclude a patient from participation in the study. Subjects who are anuric do not require a urinalysis.

[p] Serum pregnancy test required only for women of childbearing potential; do not perform for women ≥2 years postmenopausal or surgically sterile.

[q] A retinal examination for evidence of a *Candida* eye infection, including endophthalmitis or chorioretinitis, should be performed in all subjects with candidemia during Screening when possible, although the exam may occur as late as Day 7. Retinal examination is standard of care for subjects with candidemia and should be performed by an ophthalmologist, when available. The retinal examination should be repeated in subjects who were negative at baseline if the subject develops visual signs or symptoms of a *Candida* eye infection during the study. Additionally, any neutropenic subjects who recover their neutrophil counts during the study should have an additional retinal examination. Subjects who were negative at baseline and diagnosed with a *Candida* eye infection after initiating study drug must discontinue study drug but may continue in the study at the Investigator's discretion (see Section 7.4.2).

[r] A culture must be obtained as part of the standard of care for inclusion in the study. Established mycological diagnosis of candidemia or invasive candidiasis from a sample taken ≤4 days (96 hours) before randomization defined as: ≥1 blood culture positive for yeast or *Candida*, a positive test for *Candida* from sponsor-approved rapid IVD, or a positive gram stain (or other method of direct microscopy) for yeast or positive culture for *Candida* spp. from a specimen obtained from a normally sterile site. If the positive blood culture used to qualify the subject for the study is drawn >12 hours prior to randomization, an additional set of blood cultures must be obtained ≤12 hours before randomization. Identification and susceptibility testing at the local laboratory for *Candida* for blood or normally sterile tissue/fluid for culture is required at Screening and for any positive culture requiring a change of antifungal therapy (i.e., local identification and susceptibility testing is not required for *Candida* isolates cultured from specimens on other study days provided there is no required change of antifungal therapy).

[s] Specimens for culture for demonstration of efficacy, or when clinically indicated. Blood cultures should be repeated daily (preferred) or every other day until the first negative blood culture result for *Candida* spp. with no subsequent positive culture (in cases when one or more samples are drawn and cultured after the first negative culture is available). All fungal isolates cultured from blood and normally sterile tissue/fluid from Screening through Follow-up must be sent to the Central Laboratory. Record the spp. for any new bacteria isolated from blood and the spp. and susceptibilities for any new bacteria isolated from any other normally sterile site.

[t] Sponsor-approved rapid IVDs are allowed to fulfill Inclusion Criteria #3 for entry into the study. However, a culture from blood or another normally sterile site obtained during the Screening period must be positive for *Candida* spp. for the subject to be eligible for the mITT population. Currently approved rapid IVDs include the T2Candida Panel and the Septifast PCR test.

[u] Confirm all inclusion and exclusion criteria are met prior to randomization.

[v] Record all prior and/or concomitant medications from Screening through Follow-up. All systemic antifungal therapy administered within 4 weeks and all non-antifungal therapy administered within 1 week prior to randomization will be documented and recorded in the eCRF. Subjects who received systemic treatment with an antifungal agent at approved doses for treatment of candidemia for >48 hours before randomization will be excluded from the study.

[w] Adverse events are collected from the time the ICF is signed at Screening through Follow-up.

[x] The Screening period for assessing systemic signs of candidemia/ invasive candidiasis for inclusion in the study may include the 12 hours prior to the collection of the qualifying positive blood culture, when systemic signs of infection were the reason for specimen collection (blood culture or fluid from a sterile site culture or qualifying rapid IVD [e.g., T2Candida Panel]), through study randomization.

[y] Clinical response is assessed by the PI. Criteria for cure, indeterminate, and failure responses are found in Table 9.

[z] Assessment to be performed on Day 5 (i.e., not required for Day 6 and Day 7).

[aa] Subjects diagnosed with invasive candidiasis with evidence of deep tissue disease by radiology or other imaging should have follow-up imaging to demonstrate progression, stabilization, improvement, or resolution of disease. Improvement or resolution of disease by imaging would indicate radiological cure, while stabilization or progression of disease by imaging would indicate radiological failure. Missing imaging following baseline evidence of invasive candidiasis will result in an indeterminate response. Criteria for cure, indeterminate, and failure responses are found in Table 10.

[bb] Blood samples for PK analysis will be collected from the OPPOSITE arm of the infusion or through an arterial line during the infusion; after the infusion is complete, opposite arm or arterial line draws are preferred but not mandatory. Blood samples will be collected as follows: On Day 1, within 10 minutes before the end of infusion, and one sample taken anytime between end of infusion and 12 hours after the end of infusion; Day 2, 3, 4, or 5, one sample taken at any time on one day only; Day 8, pre-dose; Day 14 (for subjects not receiving a Day 15 dose), one sample taken at any time; Day 15, pre-dose (if applicable); Day 22, pre-dose (if applicable). Note: Pre-dose samples should be collected ≤30 minutes prior to the start of the infusion. For subjects receiving hemodialysis, an attempt should be made to obtain PK samples directly following hemodialysis if still within the required time window.

[cc] **Only for subjects enrolled prior to Amendment 5:** Blood samples were obtained at any time during Screening (one blood sample for protein binding and one blood sample for albumin core analyses). To limit invasive procedures these samples were ideally collected at the same time into two vacutainers, though each sample could be collected separately.

[dd] One blood sample for PK will be collected at any time on one of the following Days 2, 3, 4, or 5 (i.e., if blood sample for PK not collected on Day 2, must be collected on Day 3 [or Day 4 or Day 5]).

[ee] Day 14 draw is only for subjects completing treatment on Day 14 and should be drawn at the same time as Day 14 hematology and chemistry blood draws (i.e., random PK draw).

Table 2: Schedule of Assessments and Procedures for the Optional Treatment Period

Assessment or Procedure	Optional Treatment Period (Days) 15 (±1)	16–21	22 (±1)	23–28	EOT ≤2 days after last dose (IV or oral)	Follow-up Days 52–59
Physical examination	X	X [a]	X	X [a]	See Table 1.	See Table 1.
Neurological examination [b]	X [b]	X [b]	X [b]	X [b]		
Vital signs [c]	X	X	X	X		
Blood or normally sterile tissue/fluid for culture, if clinically indicated [d]	X	X	X	X		
Radiologic test results [e]	→	→	→	→		
Administer study drug and/or placebo (See Table 3)	X	X	X	X		
Record concomitant medications [f]	→	→	→	→		
Record adverse events [g]	→	→	→	→		
Blood for PK testing [h]	X		X			
Assess Central Venous Catheters [i]	→	→	→	→		

Abbreviations: EOT = End of Treatment; ESCMID = European Society of Clinical Microbiology and Infectious Diseases; ICF = Informed Consent Form; IDSA = Infectious Diseases Society of America; PK = pharmacokinetic.

a A focused physical exam (including a neurological exam) should be performed as clinically indicated by adverse event screening and course of the underlying disease.

b As part of the physical exam, a thorough neurologic exam is to be performed at Screening and at the EOT visit. At all other time points, a focused physical exam (including a neurological exam) is to be performed as clinically indicated, driven by the subject's signs and symptoms (see Table 1).

c Temperature and the method used [oral, rectal, temporal, tympanic, or core], heart rate, blood pressure, and respiratory rate. Axillary temperatures are allowed, but not preferred.

d Cultures from blood or other normally sterile locations only if clinically indicated. Identification and susceptibility testing at the local laboratory for *Candida* for blood or normally sterile tissue/fluid for culture for any positive culture requiring a change of antifungal therapy (i.e., local identification and susceptibility testing is not required for *Candida* isolates cultured from specimens on other study days provided there is no required change of antifungal therapy). All fungal isolates must be sent to the Central Laboratory.

e Radiologic test type with findings and interpretation should only be recorded if the test provides evidence of invasive candidiasis or evidence of progression, stabilization, improvement, or resolution of invasive candidiasis compared with previous radiographs.

f Record all concomitant medications from Screening through Follow-up (Days 52–59). Systemic antifungal therapy is especially important to document.

g Adverse events are collected from the time the ICF is signed at Screening through Follow-up (Days 52–59).

h Blood will be collected pre-dose (≤30 minutes prior to the start of the infusion).

i Presence of central line during Screening or placed during the study should be documented with dates of insertion and removal. Short-term central venous catheters (e.g., peripherally-inserted central catheters, internal jugular, or subclavian central venous catheters) and long-term central venous catheters (e.g., tunneled catheters) are recommended to be removed within 48 hours after diagnosis with candidemia, consistent with IDSA and ESCMID guidelines.

Table 3: Dosing Schedule by Day

Study Day<br>Regimen [a]	Randomized Treatment	
	**Rezafungin for Injection**	**Caspofungin [b] with Oral Step-Down Option**
**1**	Rezafungin for Injection 400 mg IV	caspofungin 70 mg IV (loading dose)
**2–3**	placebo for caspofungin IV	caspofungin 50 mg IV
**>3**	**Optional switch to oral step-down therapy allowed [c]**	
**4–7**		
**IV only**	placebo for caspofungin IV	caspofungin 50 mg IV
**oral step-down**	placebo for oral step-down therapy	oral step-down therapy (fluconazole)
**8 (±1)**		
**IV only**	Rezafungin for Injection 200 mg IV	caspofungin 50 mg IV
**oral step-down**	Rezafungin for Injection 200 mg IV<br>**-and-**<br>placebo for oral step-down therapy	placebo for Rezafungin for Injection IV<br>**-and-**<br>oral step-down therapy (fluconazole)
**9–14**		
**IV only**	placebo for caspofungin IV	caspofungin 50 mg IV
**oral step-down**	placebo for oral step-down therapy	oral step-down therapy (fluconazole)
**15–28**	**Optional treatment period begins. An End of Treatment visit is required within 2 calendar days after the last dose of study drug.**	
**15 (±1)**		
**IV only**	Rezafungin for Injection 200 mg IV	caspofungin 50 mg IV
**oral step-down**	Rezafungin for Injection 200 mg IV<br>**-and-**<br>placebo for oral step-down therapy	placebo for Rezafungin for Injection IV<br>**-and-**<br>oral step-down therapy (fluconazole)
**16–21**		
**IV only**	placebo for caspofungin IV	caspofungin 50 mg IV
**oral step-down**	placebo for oral step-down therapy	oral step-down therapy (fluconazole)
**22 (±1)**		
**IV only**	Rezafungin for Injection 200 mg IV	caspofungin 50 mg IV
**oral step-down**	Rezafungin for Injection 200 mg IV<br>**-and-**<br>placebo for oral step-down therapy	placebo for Rezafungin for Injection IV<br>**-and-**<br>oral step-down therapy (fluconazole)
**23–28**		
**IV only**	placebo for caspofungin IV	caspofungin 50 mg IV
**oral step-down**	placebo for oral step-down therapy	oral step-down therapy (fluconazole)

Abbreviation: CRRT = continuous renal replacement therapy; IV = intravenous(ly).

[a] IV only regimen means the subject has not been switched to oral step-down therapy by the Investigator. Oral step-down regimen means the subject has been switched to oral step-down therapy by the Investigator.

[b] Refer to dosing section for alternative regimens of caspofungin in subjects meeting hepatic impairment criteria or weight category criteria, and/or are receiving certain concomitant medications.

[c] Oral step-down therapy is as follows: Subjects weighing over 130 kg will not be eligible for oral step-down therapy. Subjects with a creatinine clearance >50 mL/min will receive oral fluconazole at a dose of 6 mg/kg administered once daily rounded to the nearest 200 mg increment with a maximum daily dose of 800 mg. Subjects with creatinine clearance ≤50 mL/min will receive oral fluconazole at a dose of 3 mg/kg administered once daily rounded to the nearest 200 mg increment with a maximum daily dose of 400 mg. Subjects receiving hemodialysis receive the same dose as subjects with a creatinine clearance >50 mL/min; however, they are only dosed following hemodialysis. Subjects receiving CRRT should be treated as a subject with normal renal function at 6 mg/kg rounded to the nearest 200 mg each day (Cousin 2003). Subjects receiving peritoneal dialysis should be treated with 1.5 mg/kg rounded to the nearest 200 mg each day (Cousin 2003).

Investigators are advised to pay close attention to signs of overdosing and underdosing with underweight (<50 kg) and overweight (>85 kg) subjects, respectively. Additionally, signs of potential fluconazole toxicity, including hepatic toxicity and QT prolongation, should be closely monitored for any subject on oral step-down therapy.

2.0 TABLE OF CONTENTS

PROTOCOL TITLE PAGE 1
PROTOCOL APPROVAL PAGE 2
1.0 PROTOCOL SYNOPSIS 3
2.0 TABLE OF CONTENTS 24
3.0 LIST OF ACRONYMS, ABBREVIATIONS, AND DEFINITIONS OF TERMS 30
4.0 BACKGROUND AND RATIONALE 33
4.1 Candidemia and Invasive Candidiasis 33
4.2 Rezafungin for Injection 33
4.3 Nonclinical Efficacy Models 35
4.4 Nonclinical Pharmacokinetics 35
4.5 Nonclinical Pharmacology and Toxicology 37
4.5.1 Safety Pharmacology 38
4.5.2 Nonclinical Toxicology 38
4.6 Clinical Pharmacology 40
4.7 Overview of Clinical Experience 42
4.7.1 Overview of Clinical Safety 43
4.7.2 Overview of Clinical Efficacy 43
4.8 Summary of Benefits and Risks 44
4.8.1 Known Benefits 44
4.8.2 Potential Benefits 44
4.8.3 Known Risks 44
4.8.4 Potential Risks 44
4.8.5 Mitigation of Risks 46
4.9 Justification for Dosing Regimen 46
4.9.1 Rezafungin for Injection 46
4.9.2 Caspofungin with Option for Oral Step-Down Regimen of Fluconazole 50
4.10 Population to be Studied 50
4.11 Rationale for Use of Rezafungin for Injection in Candidemia and Invasive Candidiasis 51
4.12 Statement of Compliance 52
5.0 STUDY OBJECTIVES 53
6.0 STUDY DESIGN 54
6.1 Description of the Study 54
6.1.1 Subjects Randomized to Rezafungin for Injection 54

6.1.2 Subjects Randomized to Caspofungin 54
6.1.3 End of Treatment Visit and Follow-up Visits 55
6.1.4 Mycological Diagnosis....55
6.1.5 Retinal Examination....56
6.1.6 Study Day Definition 56
6.1.7 Efficacy Assessments....56
6.1.8 Safety Assessments 57
6.1.9 PK Sampling 57
6.2 Number of Subjects....58
6.3 Measures Taken to Minimize Bias 58
6.4 Expected Duration of Subject Participation....58
6.5 Method of Treatment Assignment and Blinding 58
7.0 SELECTION, DISCONTINUATION, AND WITHDRAWAL OF SUBJECTS 60
7.1 Subject Inclusion Criteria 60
7.1.1 Rapid In Vitro Diagnostics for Candidemia and Invasive Candidiasis 61
7.1.2 Systemic Signs of Candidemia and Invasive Candidiasis 61
7.2 Subject Exclusion Criteria 62
7.3 Requalification for Entry 63
7.4 Subject Withdrawal Criteria 63
7.4.1 Withdrawal from Study Protocol 63
7.4.2 Early Discontinuation from Study Drug Administration....64
7.5 Replacement of Subjects....67
7.6 Study Termination by Sponsor and Termination Criteria....67
8.0 STUDY DRUGS 68
8.1 Rezafungin for Injection 69
8.1.1 Formulation....69
8.1.2 Directions for Use 69
8.1.3 Drug Storage 70
8.1.4 Dose Adjustment....70
8.2 Placebo and Comparator Treatment....70
8.2.1 Placebo 70
8.2.2 Caspofungin 70
8.2.2.1 Formulation....71
8.2.2.2 Directions for Use 71
8.2.2.3 Drug Storage 71

8.2.2.4 Dose Adjustment.....72
8.2.3 Oral Step-Down Therapy: Fluconazole 72
8.2.3.1 Formulation.....72
8.2.3.2 Directions for Use 72
8.2.3.3 Oral Step-Down Therapy Eligibility Criteria 73
8.2.3.4 Drug Storage 74
8.2.3.5 Dose Adjustment for Renal Impairment.....74
8.3 Compliance 74
8.4 Breaking the Blind 74
8.5 Prior and Concomitant Medications and Substances.....75
8.6 Accountability Procedures 76
8.7 Study Drug Handling and Disposal 76
9.0 STUDY PROCEDURES 78
9.1 Screening (≤4 days [96 hours]).....78
9.2 Required Treatment Period.....80
9.2.1 Day 1 80
9.2.2 Day 2 82
9.2.3 Day 3 83
9.2.4 Day 4 85
9.2.5 Days 5–7 87
9.2.6 Day 8 (±1 day) 88
9.2.7 Days 9–13 90
9.2.8 Day 14 (±1 day) 91
9.2.9 Day 30 (-2 days).....93
9.2.10 End of Treatment (≤2 days of last dose) 94
9.3 Follow-up (Days 52–59).....95
9.4 Optional Treatment Period.....97
9.4.1 Days 15 (±1 day).....97
9.4.2 Days 16–21 98
9.4.3 Day 22 (±1 day) 100
9.4.4 Days 23–28 101
10.0 ASSESSMENT OF EFFICACY.....103
10.1 Primary Efficacy Outcome 103
10.2 Secondary Efficacy Outcomes.....104
10.2.1 Mycological Response 104
10.2.2 Clinical Response.....106

10.2.3 Radiological Response 106
10.3 Exploratory Outcomes 107
11.0 ASSESSMENT OF PHARMACOKINETIC/PHARMACODYNAMIC PARAMETERS 108
12.0 ASSESSMENT OF SAFETY 109
12.1 Safety Parameters....109
12.2 Adverse Events 109
12.3 Adverse Event Reporting....110
12.3.1 Notification of Serious Adverse Events....110
12.3.2 Notification of Adverse Events of Special Interest....111
12.3.3 Notification of Emerging Safety Issues 111
12.4 Definitions....111
12.4.1 Adverse Event 111
12.4.2 Suspected Adverse Reaction 112
12.4.3 Life-Threatening AE or Life-Threatening Suspected Adverse Reaction....112
12.4.4 Serious Adverse Event or Serious Suspected Adverse Reaction....112
12.4.5 Unexpected AE or Unexpected Suspected Adverse Reaction 113
12.4.6 Emerging Safety Issue....113
12.4.7 Urgent Safety Measure....114
12.5 Adverse Event Classification....114
12.5.1 Relationship to Investigational Drug 114
12.5.2 Severity 114
12.5.3 Serious Adverse Event 115
12.6 Adverse Event Follow-up 115
12.7 Adverse Events of Special Interest 115
12.7.1 Intravenous Infusion Intolerability....116
12.7.2 Phototoxicity 116
12.7.3 Ataxia, Neuropathy, and Tremors....116
12.8 Adverse Event Management....116
12.9 Risks for Women of Childbearing Potential or During Pregnancy 116
12.10 Risks for Males of Childbearing Potential....117
13.0 STATISTICAL METHODS 118
13.1 Analysis Populations....118
13.2 Subject Characteristics....118
13.3 Study Drug Exposure....119
13.4 Efficacy Analyses 119

13.4.1 Primary Efficacy Analyses for the FDA 119
13.4.1.1 Additional Analyses of the FDA Primary Efficacy Outcome 120
13.4.2 Primary Efficacy Analyses for the EMA 120
13.4.3 Secondary Efficacy Analyses 122
13.4.4 Additional Efficacy Analyses 123
13.5 Safety Analyses 123
13.6 Health Economics Outcome Research 124
13.7 Pharmacokinetic Analyses 124
13.8 Determination of Study Sample Size 124
13.9 Data and Safety Monitoring Board 125
13.10 Handling of Dropouts and Missing, Unused, and Spurious Data 125
13.11 Subject Disposition 125
13.12 Deviation Reporting 125
13.13 Impact of the COVID-19 Pandemic on Analyses 125
14.0 INVESTIGATOR REQUIREMENTS 127
14.1 Protocol Adherence 127
14.2 Electronic Case Report Forms 127
14.3 Source Document Maintenance 127
14.4 Study Monitoring Requirements 127
14.5 Study Completion 128
15.0 QUALITY CONTROL AND QUALITY ASSURANCE 129
16.0 PROTECTION OF HUMAN SUBJECTS 130
16.1 Informed Consent 130
16.2 IRB/IEC Approval 130
17.0 DATA HANDLING AND RECORD KEEPING 131
17.1 Direct Access to Source Data/Documentation 131
17.2 Study Drug Accountability 131
17.3 Retention of Records 131
18.0 FINANCING AND INSURANCE 133
19.0 PUBLICATION POLICY 134
20.0 REFERENCES 135
21.0 APPENDICES 139

LIST OF TABLES

Table 1: Schedule of Assessments and Procedures for the Required Treatment, End of Treatment, and Follow-up Periods 17
Table 2: Schedule of Assessments and Procedures for the Optional Treatment Period 21
Table 3: Dosing Schedule by Day 22
Table 4: Predicted Pharmacokinetic-Pharmacodynamic Target Attainment for Rezafungin Regimens, Stratified by Week and Minimal Inhibitory Concentration, Against *Candida albicans* 48
Table 5: Predicted Pharmacokinetic-Pharmacodynamic Target Attainment for Rezafungin Regimens, Stratified by Week and Minimal Inhibitory Concentration, Against *Candida glabrata* 49
Table 6: Percent Probabilities of Pharmacokinetic-Pharmacodynamic Target Attainment by Anidulafungin, Caspofungin, Micafungin, and Rezafungin against *Candida* spp. 50
Table 7: Global Response 103
Table 8: Mycological Response Definitions 104
Table 9: Investigator's Assessment of Clinical Response Definitions 106
Table 10: Radiological Response Definitions 107
Table 11: Guidelines for Assessing Relationship of Event to Study Drug 114
Table 12: Guidelines for Severity Assessment by the CTCAE Version 5.0 115

LIST OF FIGURES

Figure 1: Study Design Diagram 14
Figure 2: Dosing Schedule for Required Treatment Period 15
Figure 3: Dosing Schedule for Optional Treatment Period 16
Figure 4: Rezafungin Penetrates and Accumulates at a Higher Level at Site of Liver Infection than Micafungin 37
Figure 5: Predicted Pharmacokinetic-Pharmacodynamic Target Attainment for Rezafungin Regimens, Against *Candida albicans* Relative to MIC Distribution 48
Figure 6: Predicted Pharmacokinetic-Pharmacodynamic Target Attainment for Rezafungin Regimens, Against *Candida glabrata* Relative to MIC Distribution 49

3.0 LIST OF ACRONYMS, ABBREVIATIONS, AND DEFINITIONS OF TERMS

ACM	all-cause mortality
ADL	activities of daily living
AE	adverse event
AESI	adverse event of special interest
ANC	absolute neutrophil count
APACHE II	Acute Physiology and Chronic Health Evaluation
AUC	area under the concentration-time curve
AUC_{0-t}	area under the concentration-time curve from time 0 to the final sample
BCRP	breast cancer resistance protein transporter
BMI	body mass index
CDC	Centers for Disease Control and Prevention
CE	clinically evaluable
CI	confidence interval
C_{max}	maximum plasma concentration
COVID-19	Coronavirus disease 2019
CRRT	continuous renal replacement therapy
CTCAE	common terminology criteria for adverse events
CYP	cytochrome P450
DDI	drug-drug interaction
DRC	Data Review Committee
DSMB	Data and Safety Monitoring Board
ECG	electrocardiogram
eCRF	electronic case report form
EDC	electronic data capture
EOT	End of Treatment
EMA	European Medicines Agency
ESCMID	European Society of Clinical Microbiology and Infectious Diseases
*f*AUC	free-drug area under the rezafungin concentration-time curve
fAUC_{0-168}	free-drug area under the rezafungin concentration-time curve from time 0 to 168 hours
FDA	Food and Drug Administration
GCP	Good Clinical Practice
GLP	Good Laboratory Practice
GS	1,3-β-D-glucan synthase
hERG	human ether-à-go-go-related gene
IC_{50}	half maximal inhibitory concentration

ICF	informed consent form
ICH	International Council for Harmonisation
ICU	intensive care unit
IDSA	Infectious Diseases Society of America
IEC	Independent Ethics Committee
IFD	invasive fungal disease
IM	intramuscular
IME	important medical event terms
IND	investigational new drug
IP	intraperitoneal
IRB	Institutional Review Board
IRT	interactive response technology
ITT	intent-to-treat
IV	intravenous(ly)
IVD	in vitro diagnostic
KM	Kaplan-Meier
LC-MS/MS	liquid chromatography–tandem mass spectrometry
LOAEL	lowest-observed-adverse-effect level
MAD	multiple-ascending dose
MALDI	matrix-assisted laser desorption ionization
MALDI-MS	matrix-assisted laser desorption ionization mass spectrometry
MALDI-MSI	matrix-assisted laser desorption ionization mass spectrometry imaging
MATE	multidrug and toxin extrusion
MedDRA	Medical Dictionary for Regulatory Activities
MIC	minimal inhibitory concentration
MIC_{50}	minimal inhibitory concentration required to inhibit the growth of 50% of the isolates
MIC_{90}	minimal inhibitory concentration required to inhibit the growth of 90% of the isolates
mITT	modified intent-to-treat
NI	non-inferiority
NOAEL	no-observed-adverse-effect level
OATP	organic anion-transporting polypeptide transporter
OCT	organic cation transporter
PCP	*Pneumocystis* pneumonia
PCR	polymerase chain reaction
PCS	potentially clinically significant

PD	pharmacodynamic(s)
P-gp	P-glycoprotein transporter
PI	Principal Investigator
PK	pharmacokinetic(s)
PT	Preferred Term
PT/INR	Prothrombin Time or International Normalized Ratio
QTcF	QT interval corrected for heart rate by Fridericia's formula
SAD	single-ascending dose study
SAE	serious adverse event
SAER	serious adverse event report
SAP	statistical analysis plan
SARS-CoV-2	severe acute respiratory syndrome coronavirus 2
SC	subcutaneous
SmPC	Summary of Product Characteristics
SOC	System Organ Class
SOP	Standard Operating Procedure
spp.	species
$t_{½}$	half-life
TEAE	treatment-emergent adverse event
T_{max}	time to C_{max}
ULN	upper limit of normal
US	United States
UV	ultraviolet
V_{ss}	volume of distribution at steady state
V_z	volume of distribution

4.0 BACKGROUND AND RATIONALE

4.1 Candidemia and Invasive Candidiasis

Rezafungin for Injection, a new echinocandin antifungal agent, is being developed to treat patients with systemic infections caused by *Candida* species (spp.). These serious and life-threatening infections represent a significant public health issue, particularly in highly vulnerable patient populations such as the elderly, post-surgical, critically ill, and other hospitalized patients with serious medical conditions (Magill 2014; Andes 2012; Wisplinghoff 2004). In addition, because of increasing resistance to existing antifungal drugs, there is an urgent need to develop new and more effective antifungal agents to treat these serious infections (Alexander 2013; Arendrup 2013; Ostrosky-Zeichner 2013; Pfaller 2012). The Centers for Disease Control and Prevention (CDC) recently warned that fluconazole-resistant *Candida* spp. have the potential to pose a serious threat to public health (CDC 2013; CDC 2016). However, since 2007, no new antifungal agents have been approved for treatment of candidemia (The White House 2014; Executive Office of the President 2014; CDC 2013).

There are currently 3 commercially authorized echinocandins in the United States (US) and the European Union (EU): caspofungin, micafungin, and anidulafungin. These echinocandins are administered once daily via intravenous (IV) infusion and share similar pharmacokinetic (PK) parameters and safety profiles (Eraxis [anidulafungin] Prescribing Information; Mycamine [micafungin sodium] Prescribing Information; Cancidas [caspofungin acetate] Prescribing Information; Caspofungin acetate Prescribing Information; Ecalta [anidulafungin] Summary of Product Characteristics; Mycamine [micafungin sodium] Summary of Product Characteristics; Cancidas [caspofungin acetate] Summary of Product Characteristics).

Caspofungin, the first US Food and Drug Administration (FDA) approved echinocandin, originated from the "pneumocandin" series of compounds, so named as they were first developed for *Pneumocystis* pneumonia (PCP), an Ascomycetes fungus with a high amount of 1,3-β-D-glucan in cell walls (Balkovec 2014). Development of the drug for this use was discontinued when PCP was considered less relevant in autoimmune deficiency syndrome (AIDS) patients due to advances in human immunodeficiency virus (HIV) therapy. Development was subsequently restarted for treatment of candidemia.

Echinocandins are considered the first-line therapy for candidemia and invasive candidiasis by the Infectious Diseases Society of America (IDSA) guidelines (Pappas 2016) and the European Society of Clinical Microbiology and Infectious Diseases (ESCMID) guidelines (Cornely 2012; Ullmann 2012), regardless of immunosuppression.

4.2 Rezafungin for Injection

Rezafungin is a novel semi-synthetic echinocandin synthesized from a fermentation product of *Aspergillus nidulans* that is administered by IV infusion.

Rezafungin is a water soluble and stable echinocandin with an extended half-life ($t_{½}$) compared with echinocandins that have been approved for marketing. Rezafungin has a choline moiety at the C5 position of the 4,5-dihydroxyornithine residue of the cyclic hexapeptide core that affords greater stability as a solid, in aqueous buffered solutions, and in plasma relative to marketed echinocandins.

Rezafungin acts through inhibiting the synthesis of 1,3-β-D-glucan, an essential component of the fungal cell wall of *Candida* species and regions of active cell growth of *Aspergillus* hyphae. Because 1,3-β-D-glucan is also present in the asci (cyst) form of *Pneumocystis* spp., rezafungin depletes asci and inhibits the organism from completing sexual reproduction and proliferation. The synthesis of 1,3-β-D-glucan is dependent upon the activity of 1,3-β-D-glucan synthase (GS), an enzyme complex in which the catalytic subunit is encoded by *FKS1*, *FKS2*, and *FKS3* genes. Inhibition of GS by echinocandins results in rapid, concentration-dependent, fungicidal activity for *Candida* spp. 1,3-β-D-Glucan is not present in mammalian cells.

Across in vitro studies performed to date, rezafungin consistently demonstrates potent activity against *Candida* spp. and *Aspergillus* spp. at concentrations comparable to other echinocandins. Rezafungin is included in the annual SENTRY international fungal surveillance program. In a pooled analysis of the 2016-2018 SENTRY data, rezafungin generated $MIC_{50/90}$ values of 0.03/0.06 µg/mL against *Candida albicans*, *Candida tropicalis*, and *Candida krusei*, 0.06/0.12 µg/mL against *Candida glabrata* and *Candida dubliniensis*, and 1/2 µg/mL against *Candida parapsilosis*. For the subset of 20 characterized *fks* hot spot mutants (*C. glabrata* n = 17, *C. albicans* n = 1, and *C. tropicalis* n = 2), rezafungin minimal inhibitory concentration (MIC) values ranged from 0.06–2 µg/mL. Overall, rezafungin activity was comparable to activity of anidulafungin, caspofungin, and micafungin, against the same wild-type and *fks* mutant isolates (Pfaller 2020).

When tested against multiple clinical isolates of wild-type and echinocandin-resistant and/or azole-resistant *Candida* spp., rezafungin activity was similar with that of anidulafungin versus all strains tested with minimal inhibitory concentration required to inhibit the growth of 90% of the isolates tested (MIC_{90}) values ≤1 µg/mL for all species except *C. parapsilosis*, which was 2 µg/mL, consistent with the susceptibility profile of this species to echinocandins. The potency of rezafungin against strains with documented fks mutations was 2- to 8-fold greater than that of caspofungin and similar with that of anidulafungin.

The activity of rezafungin was also tested against *Candida auris*, an emerging fungal pathogen that presents a serious global health threat. When evaluated against a CDC panel of 100 *C. auris* isolates representing 4 clades, rezafungin demonstrated strong activity with minimal inhibitory concentration required to inhibit the growth of 50% of the isolates (MIC_{50}) and MIC_{90} values of 0.125 and 0.5 µg/mL, respectively (Berkow 2018).

4.3 Nonclinical Efficacy Models

A series of studies in the neutropenic mouse systemic candidiasis model show that rezafungin is efficacious when administered by either the IV, intraperitoneal (IP) or subcutaneous (SC) route across a wide range of doses. Dose-dependent reductions of *C. albicans* kidney burden have been observed in all studies. The lowest dose level to achieve a significant reduction in kidney burden in this model was 0.5 mg/kg IP and 0.6 mg/kg IV, both given once. Higher doses (≥1 mg/kg) have demonstrated a significant reduction in kidney burden relative to vehicle control for up to 7 days following a single IP dose. Rezafungin was also shown to be efficacious at doses of 3 to 30 mg/kg against azole-resistant *C. albicans* spp. R357 and DPL001 in separate studies. For efficacy against echinocandin-resistant *C. albicans* strains, a heterozygous and a homozygous *fks* mutant strain with elevated minimal inhibitory concentration (MIC) and half maximal inhibitory concentration (IC_{50}) values for micafungin and rezafungin were studied. Rezafungin at dose levels (10 to 60 mg/kg) selected to estimate the anticipated human exposure of once weekly Rezafungin for Injection, was found to be significantly more active than micafungin, especially at 48 hours. Against the highly resistant homozygous *fks* mutant, improved survival was noted only in the highest rezafungin dose group suggesting that a high dose of rezafungin may be efficacious to control severe echinocandin-resistant candidiasis where administration of the human therapeutic exposures of micafungin loses effect. Rezafungin, given IV twice a day for 5 days in the treatment of murine *A. fumigatus* septicemia, was associated with a significant increase in the 10-day survival compared to the vehicle group.

In response to *C. auris* as an emerging fungal pathogen that presents a serious global health threat, direct evidence of the in vivo efficacy of rezafungin in animal models of infection was obtained. Rezafungin at human exposure equivalent to 400 mg dose (mouse 20 mg/kg IP) was effective as treatment in neutropenic mouse models of disseminated *C. auris* candidiasis as measured by increased survival rate or by colony forming unit reduction compared with micafungin, amphotericin B, or fluconazole.

4.4 Nonclinical Pharmacokinetics

Rezafungin was found to be stable in liver microsomes from all spp. tested. A cross-species stability study also determined that rezafungin is stable in intestinal microsomes and no metabolic products were identified during incubations of rezafungin with mouse, rat, monkey, or human liver microsomes, indicating good metabolic stability. In vivo, metabolite profiling of rat plasma and excreta samples is consistent with in vitro results confirming the lack of significant biotransformation.

The PK profile of rezafungin was investigated in mice, rats, dogs, cynomolgus monkeys, and chimpanzees following IV administration, the intended clinical route of administration, and also after IP administration to mice, intramuscular (IM) to rats, SC to rats and cynomolgus monkeys, and orally to dogs, monkeys, and chimpanzees. Rezafungin consistently exhibited very low clearance, modest volume of distribution (V_z) or volume of distribution at steady state (V_{ss}), and long $t_{½}$. Across all spp. tested, rezafungin exhibited a favorable PK profile, mainly

attributable to lower clearance (resulting in a longer $t_{½}$). Additionally, there was low to no drug accumulation or gender differences found with rezafungin across all spp. tested after multiple doses and routes of administration.

Tissue distribution of rezafungin was evaluated in rats and mean area under the concentration-time curve (AUC) from time 0 to the final sample (AUC_{0-t}) was lowest in brain tissue and highest in kidney tissue. Calculation of tissue/plasma AUC ratios indicated exposure relative to plasma was comparable between major organs (4- to 5-fold higher in kidney, lung, liver, and spleen) with the exception of much lower ratios in tissue from heart and brain (≤1). More importantly, rezafungin appears to concentrate within fungal lesions even after the majority of drug has been cleared from an infected organ, as shown in a separate study by Perlin and colleagues (Zhao 2016). In the study, the authors utilized matrix-assisted laser desorption ionization (MALDI) imaging mass spectrometry technology to image kidney and liver cross-sections of mice with invasive candidiasis. MALDI imaging allowed for multiplexed analysis of different molecules simultaneously in the same tissue section, providing semi-quantitative information of fungal lesions and drug localization. From cross-sections of liver and kidney, across different time points from 1 to 48 hours, Perlin and colleagues noted that rezafungin tissue levels begin to decline after reaching the maximum plasma concentration (C_{max}) at 6 hours post-dose but increase and concentrate within fungal lesions at later time points (24 and 48 hours).

A second study was carried out using a mouse model of intra-abdominal *C. albicans* candidiasis to determine drug distribution and concentration of rezafungin in infected tissues by matrix-assisted laser desorption ionization mass spectrometry imaging (MALDI-MSI) and liquid chromatography–tandem mass spectrometry (LC-MS/MS) quantitation in livers and kidneys of infected mice (Zhao 2017). *C. albicans* SC5314 was inoculated at 1×10^7 CFU in sterile stools and saline to induce peritonitis and abscess formation. A single dose of rezafungin at 20 mg/kg (equivalent to human 400 AUC exposure) was given on Day 3 post-inoculation and compared with once daily treatment of micafungin at 5 mg/kg (equivalent to human 150 mg therapeutic exposure) starting on Day 3 post-inoculation, with a total of 3 doses of micafungin administered. Livers and kidneys were collected up to 72 hours post first dose of each drug for MALDI imaging as well as drug concentration determination.

It was observed that even as drug levels in the tissue declined, rezafungin was concentrated within the lesion 6 hours post-dose in both liver and kidney tissue. For example, it was noted that rezafungin had an extensive tissue distribution with a drug level of 80.1 μg/mL in the non-lesion part of liver at 6 hours after a single dose treatment at 20 mg/kg (Zhao 2017; Figure 4). Importantly, rezafungin was observed to quickly penetrate into abscesses as early as 3 hours and rapidly reach the necrotic core interacting with the main fungal population at 6 hours, with an average of 31.6 μg/mL drug in lesions (Zhao 2017). Given the long $t_{½}$ of rezafungin, sustained drug penetration and accumulation within the lesion was continuously observed for all remaining time points included in the study. At the last time point of 72 hours following a single dose of rezafungin, drug levels inside the lesions were still close to 30 μg/mL, about 6-fold higher than

that for micafungin at steady-state (after 3 doses). Micafungin was found to penetrate more slowly into liver and kidney abscesses and reached detectable levels inside lesions at 6 hours after the first dose. The penetration improved upon multiple doses of treatment, and only at steady-state were drug signals observed from the necrotic core where a large number of fungal cells were found to proliferate. In this in vivo experiment, rezafungin demonstrated superior penetration and concentration at the site of the abscess versus micafungin suggesting that rezafungin offers the potential to fill an unmet need in invasive candidiasis patients.

Figure 4: Rezafungin Penetrates and Accumulates at a Higher Level at Site of Liver Infection than Micafungin

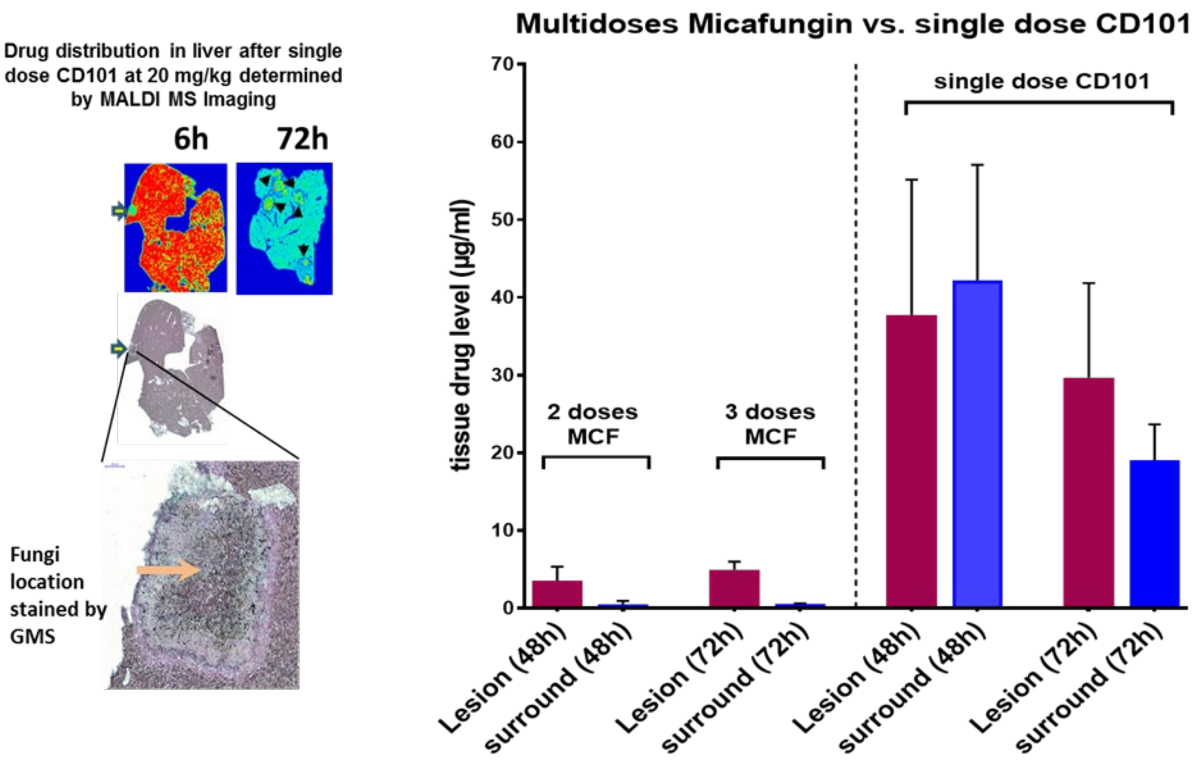

Abbreviations: GMS = Gömöri methenamine silver stain; MALDI-MS = matrix-assisted laser desorption ionization mass spectrometry; MCF = micafungin.

With low clearance, a long $t_{½}$, widespread penetration into tissues, no biotransformation, and extensive excretion as unchanged drug, rezafungin is suitable for once weekly treatment, providing high drug exposures relative to other echinocandins for candidemia and invasive candidiasis.

4.5 Nonclinical Pharmacology and Toxicology

The summary of nonclinical information is considered accurate as of the date of this protocol amendment. The Investigator's Brochure should be referenced for additional details or updated information throughout the study.

An overview of information on the efficacy, PK, and toxicology of rezafungin in animal models is provided below. Additional nonclinical data is available in the Investigator's Brochure.

4.5.1 Safety Pharmacology

The summary of safety pharmacology information provided in the protocol is considered accurate as of the date of this protocol amendment. The Investigator's Brochure should be referenced for additional details or updated information throughout the conduct of the study.

In the safety pharmacology studies, rezafungin was tested for effects on neurobehavioral, cardiovascular (hemodynamic and electrocardiographic), and respiratory functional endpoints. There were no clinical observations or statistically significant changes in neurobehavioral parameters or body temperatures that were attributed to administration of rezafungin when administered once every 3 days over one week by IV slow bolus to male rats at doses up to 45 mg/kg, nor were there changes in the gross behavioral, physiological, or neurological state of the animals.

Rezafungin did not affect human ether-à-go-go-related gene (hERG) current at concentrations up to 1.1 μM, the maximum concentration able to be evaluated, which exceeds the estimated free drug C_{max} in the clinic. At a total C_{max} plasma level of 22.68 μg/mL observed in a study in healthy human subjects, the free drug C_{max} plasma level is 0.43 μM, which is lower than the maximum concentration tested in the hERG assay.

In a study in cynomolgus monkeys, there were no rezafungin-related changes in blood pressure, electrocardiographic, or respiratory parameters, or in body temperature, but there was a minimal and transient decrease in heart rate relative to vehicle control in restrained cynomolgus monkeys at ≥3 mg/kg that was of uncertain relationship to rezafungin. The no-observed-adverse-effect level (NOAEL) for cardiovascular and respiratory functions following a 20-minute IV infusion of rezafungin to male radio telemetry instrumented cynomolgus monkeys was 10 mg/kg, the highest dose tested.

4.5.2 Nonclinical Toxicology

The summary of nonclinical toxicology information provided in the protocol is considered accurate as of the date of this protocol amendment. The Investigator's Brochure should be referenced for additional details or updated information throughout the conduct of the study.

Nonclinical safety of rezafungin was evaluated in 1) single and repeat dose toxicity studies up to 4 weeks in duration in rats and cynomolgus monkeys, 2) in vitro and in vivo in genotoxicity studies, 3) in vitro and in vivo phototoxicity, and 4) female fertility and early embryonic development in pregnant rats and embryo-fetal development in pregnant rats and rabbits.

Findings from nonclinical toxicology studies are summarized below. The Investigator's Brochure should be referenced for more information.

- Adverse toxicology findings were seen only in rats and were related to transient elevations in plasma histamine-associated acute clinical signs that were transient after the first dose in surviving rats and generally not seen after the third or fourth dose.
- Important target organ toxicities (hepatotoxicity, renal toxicity, and skeletal muscle changes) reported with marketed echinocandins were not seen with rezafungin.
- Rezafungin did not affect the gross behavioral, physiological, or neurological state of rats.
- In a combined cardiovascular and respiratory study in cynomolgus monkey, there were no rezafungin-related changes in blood pressure, electrocardiogram (ECG) and respiratory parameters, and body temperature.
- Mild rezafungin-induced hematological changes observed in rats primarily at the high dose, were not associated with histopathology, were not considered adverse, and were not seen in monkeys.
- Minor clinical chemistry changes were noted in rats and monkeys at the higher doses used in the toxicology studies.
- Rezafungin caused local vascular irritation in rats, but not in monkeys; local vascular injury in rats was confined to high dose males during the 4-week toxicity study.
- Dose-related and transient acute histamine-mediated effects in rats associated with mortality at 15 and 45 mg/kg. The NOAEL for these C_{max}-driven acute effects was 5 mg/kg, which generated C_{max} values of 8.04 and 10.4 µg/mL, in males and females respectively, after the first dose. These species-specific (rat) histamine effects are consistent with observations for marketed echinocandins.
- In a fertility study in male rats, no rezafungin-related effects were seen on reproductive performance up to the highest dose tested (45 mg/kg), dose-related lower mean sperm motility, concentration, and/or increased incidence of abnormal sperm morphology, along with microscopic findings of testicular seminiferous tubular epithelial degeneration associated with an effect on spermiation were noted at ≥30 mg/kg. Based on these data, the NOAEL for male reproductive toxicity was 15 mg/kg (2.5-fold over the estimated steady state plasma level in humans at 200 mg). Based on the lack of any effects on intrauterine survival in females at any dosage level, the NOAEL for early embryonic toxicity was 45 mg/kg, the highest dose evaluated.
- Rezafungin was administered IV to cynomolgus monkeys at 3, 10, 30, and 60/45 mg/kg once every 3 days for 3 consecutive months. The high dose of 60 mg/kg was lowered to 45 mg/kg on Day 42 (during the seventh week of dosing) due to unexpected neurobehavioral effects and the 60/45 mg/kg group females were terminated on Day 49 (during the eighth week of dosing) due to adverse clinical observations, including severe tremors. Throughout the remainder of the study, dose-related incidence of slight to moderate tremors were observed sporadically at ≥30 mg/kg. These lower grade tremors did not interfere with the daily normal function of the animals and demonstrated

reversibility during the recovery period and were therefore considered non-adverse. Rezafungin-related microscopic findings of Schwann cell inclusions (interpreted as phospholipidosis) were noted in the sensory ganglia at ≥3 mg/kg and in peripheral nerves at ≥30 mg/kg, with partially reversible Schwann cell hyperplasia in the ganglia at ≥30 mg/kg; both changes are considered adaptive and nonadverse. Transmission electron microscopy confirmed the presence of phospholipidosis in Schwann cells and found no evidence of axonal degeneration or axonopathy.

- The NOAEL level for neuropathology in monkeys is 30 mg/kg, which is approximately 11.4-fold over the human plasma exposure.
- As a follow-up to the 13-week GLP monkey study, an investigative 13-week study in female monkeys was conducted to evaluate the onset of neurotoxicity, through detailed neurobehavioral assessments and quantitative measures of nerve conduction velocities and amplitudes, as well as histopathological changes, and reversibility of any findings over a longer recovery period (13-weeks). Neurobehavioral findings consisted of barely perceptible (slight to moderate) tremors in limbs of all animals administered 30 mg/kg rezafungin beginning at Week 4; tremors reversed in all recovery group animals by recovery Day 30. Nerve conduction findings consisted of no change in nerve conduction pre-dose and after 5 and 8 weeks of dosing. After 13 weeks of dosing, minimal changes (<10% decrease in conduction) of peroneal motor nerve and sural sensory nerve; cauda equina measure was similar between rezafungin and control groups. Clear evidence of full recovery was observed in peroneal and sural nerve conduction measures by first assessment in recovery Week 7. Nervous system histopathology (after 13 weeks of dosing) consisted of Schwann cell inclusions (considered lysosomal accumulations of myelin/lipids). However, as evidenced in the initial GLP 13-week study, there was no evidence of axonal degeneration, thinning of myelin sheaths, or demyelination in the 13-week investigative study, corroborating these findings.
- Rezafungin was negative for mutagenic activity and induction of chromosome aberrations in vitro, and did not increase micronucleated polychromatic erythrocytes in the in vivo bone marrow micronucleus study in rats.
- Rezafungin induced a phototoxicity response in vitro and in rats in which multiple doses generated a minimal phototoxic response at plasma concentrations 3.6-fold above those achieved clinically.

4.6 Clinical Pharmacology

The summary of clinical pharmacology information provided in the protocol is considered accurate as of the date of this protocol amendment. The Investigator’s Brochure should be referenced for additional details or updated information throughout the conduct of the study.

Long Half-Life and Dose-Proportional

The plasma PK of rezafungin was generally well-characterized following single doses of 50, 100, 200, and 400 mg (Studies CD101.IV.1.01 and CD101.IV.1.02). Exposure to rezafungin increased with increasing Rezafungin for Injection doses. Time to reach maximum plasma concentration (i.e., T_{max}) was observed at the end of infusion, as expected, at approximately 1 hour after the start of study drug infusion for all doses. Elimination of rezafungin appears multiphasic. Area under the concentration-time curve (AUC) and C_{max} increased in a dose proportional manner indicating linear kinetics. Total body clearance was slow (approximately 0.2 L/h) throughout the dose levels with a long apparent terminal half-life ($t_{½}$ ranged from 127-146 hours). Volume of distribution (V_z) and volume of distribution at steady state (V_{ss}) ranged from 33 to 48 L. The fraction of dose excreted in urine was <1% at all dose levels, indicating minor contribution of renal clearance in rezafungin excretion. The plasma PK of rezafungin was well characterized following 2 or 3 once weekly doses of rezafungin: 100 mg on Days 1 and 8; 200 mg on Days 1 and 8; and 400 mg on Days 1, 8, and 15. Exposures following the first dose were comparable to that observed in the single ascending dose (SAD) study, with AUC and C_{max} generally increasing in a dose proportional manner. Minor accumulation was observed for the 400 mg dose; 1.74-fold as measured by C_{max} ratio or 1.55-fold as measured by the area under the concentration time-curve from time 0 to 168 hours (AUC_{0-168}) ratio of last/first dose after 3 doses.

Lack of Drug-Drug Interactions

Pharmacokinetic results were obtained in a drug-drug interaction (DDI) study (Study CD101.IV.1.09) in 26 healthy subjects to assess effect of rezafungin on several substrates of cytochrome P450 (CYP) enzymes and/or drug transport proteins. Subjects were dosed each of three cocktails (tacrolimus and repaglinide; metformin, rosuvastatin, and pitavastatin; caffeine, efavirenz, midazolam, and digoxin) alone and in combination with rezafungin in a single sequence crossover study. Rezafungin was dosed at 600 mg on Day 1, and at 400 mg once weekly for two weeks to match peak exposure at the highest dose tested in Phase 2 with the first and subsequent doses. The geometric mean ratio of and 90% confidence interval (CI) for each probe drug when administered with rezafungin relative to when the probe drug given alone were largely within the default no effect boundary of 80–125%. No clinically significant drug interactions were observed, and no dose adjustments were necessary for these agents when combined with rezafungin.

Elimination Primarily Non-Renal, with Inactive Metabolites Excreted in Urine

During a study with [^{14}C]rezafungin for Injection (Study CD101.IV.1.12), the radiocarbon was primarily excreted in feces. Based on interpolated data (using data from the subjects' return visits to the clinical research unit [CRU] on Days 29 and 60), it was estimated that the majority of the dose (an overall mean estimate of 88.3%) would have been recovered had the subjects been continuously confined to the clinic through Day 60. Approximately 74.3% of this total was

recovered in feces and the total in urine was 25.7%, indicating that elimination is primarily nonrenal.

Radiochemical analysis indicated that radioactivity in urine was primarily due to metabolites hydroxylated (in one of three positions) or dealkylation (resulting in des-pentyl), whereas radioactivity in feces was primarily rezafungin. In plasma, metabolites were present at low levels relative to parent. The AUC of rezafungin accounted for ~77% of total radiocarbon AUC, with individual metabolites accounting for less than 10% each.

Based on a lack of correlation between creatinine clearance and rezafungin PK in a population PK analysis, renal elimination does not appear to be an important route of elimination for rezafungin. Therefore, no adjustment of dose is proposed for subjects with renal impairment.

No Effect on QT Interval

Rezafungin for Injection, in single doses up to 1400 mg did not prolong the QT interval (Study CD101.IV.1.06). There were no clinically significant findings in other cardiac parameters, including heart rate, PR interval, and QRS interval, between Rezafungin for Injection 600 mg or 1400 mg and placebo.

Echocardiogram results post-dose were normal in all subjects indicating no effect on cardiac contractility or ejection fraction for single doses of rezafungin up to 1400 mg.

Mild Increased Photosensitivity

Due to results from a nonclinical phototoxicity study in rats, the photosensitivity of Rezafungin for Injection was examined in an assessor-blinded Phase 1 study (Study CD101.IV.1.07), with a 400 mg once weekly regimen administered (which provided almost double the exposure compared to the Phase 3 and proposed marketed dose regimen of 400 mg during the first week followed by 200 mg once weekly thereafter). The study demonstrated that within skin types and across multiple time points, there was mildly increased photosensitivity to rezafungin. One subject in the Phase 2 study (Study CD101.IV.2.03) had a treatment-emergent adverse event (TEAE) of sunburn following substantial sun exposure potentially representing photosensitivity. Future rezafungin subjects and patients are advised to avoid sun exposure and other sources of ultraviolet (UV) radiation without adequate protection. Photosensitivity is an adverse event of special interest (AESI), and investigators should report any AE potentially related to photosensitivity to the Sponsor.

4.7 Overview of Clinical Experience

The summary of clinical safety and efficacy information provided in the protocol is considered current and accurate as of the date of this protocol amendment. The Investigator’s Brochure should be referenced for additional details or updated information throughout the conduct of the study.

4.7.1 Overview of Clinical Safety

In the Phase 2 STRIVE study (CD101.IV.2.03), there were no notable differences in TEAEs across the study groups. Between the rezafungin groups, there were no dose-related patterns in study drug-related TEAEs, severe TEAEs, serious adverse events (SAEs), related SAEs, or SAEs leading to death. There were no discernable differences between TEAE categories observed for the Rezafungin for Injections groups compared to caspofungin with the exception of the higher study drug discontinuation for the Rezafungin for Injection regimen of 400 mg once weekly up to 4 weeks, a higher dosing regimen than the one selected for ongoing or future studies. Most adverse events (AEs) were mild or moderate in severity.

Adverse Events from tremor or neuropathy were equally represented between rezafungin and caspofungin arms (3%) in the Phase 2 STRIVE study.

There was one episode of infusion reaction in the Phase 2 study that occurred with the fourth dose in the 400 mg once weekly dosing group. All symptoms resolved after stopping the infusion with no further interventions. The infusion was not restarted.

In the Phase 1 multiple ascending dose study, 4 subjects in the Rezafungin for Injection group experienced mild, transient infusion reactions, characterized by flushing, sensation of warmth, nausea, and chest tightness. These infusion reactions were associated primarily with the third 400 mg once weekly dose, occurred within minutes of infusion initiation, and disappeared within minutes without interruption or discontinuation of the study drug infusion. No pharmacological or procedural interventions were required or instituted for the infusion reactions. In another Phase 1 study (CD101.IV.1.07), an infusion reaction occurred during the fourth dose of a 400 mg once weekly for 4 weeks regimen. The third dose had been administered only 3 days prior (instead of 7 days) due to a delay in the return of the subject to the clinic. Symptoms of the infusion reaction resolved within a few minutes of infusion interruption without pharmacological interventions. The infusion was then restarted and completed at half the usual infusion rate with no resulting adverse symptoms or signs.

The Investigator's Brochure can be referenced for more details on the safety of rezafungin.

4.7.2 Overview of Clinical Efficacy

In the Phase 2 STRIVE study, demographics were generally balanced across the groups with no differences in sex, race, ethnicity, or age that would appreciably affect clinical efficacy. Diagnosis at study entry was 79.2% candidemia and 20.8% invasive candidiasis with reasonable balance across the treatment groups. The study demonstrated efficacy for Rezafungin for Injection comparable to standard of care at Day 14 for all endpoints. The Rezafungin for Injection regimen starting with a 400 mg once weekly loading dose followed by 200 mg once weekly for a total of 2 to 4 doses, had robust results for the primary endpoint of overall success compared with standard of care (Rezafungin for Injection, 76.1%; caspofungin, 67.2%).

Secondary endpoints of mycological success, Investigator assessment of clinical cure, and all-cause mortality supported the primary endpoint.

4.8 Summary of Benefits and Risks

4.8.1 Known Benefits

There are no known benefits of treatment with Rezafungin for Injection as of the date of the original protocol for this study.

The therapeutic benefits of caspofungin and fluconazole for the treatment of fungal infections are documented in the prescribing information and published literature. Approximately half of the subjects enrolled in this study will be randomized to receive IV caspofungin with the option of oral fluconazole step-down therapy.

4.8.2 Potential Benefits

In the Phase 2 STRIVE study, efficacy for Rezafungin for Injection was comparable to standard of care at Day 14 for all endpoints.

4.8.3 Known Risks

Infusion reactions characterized by flushing, shortness of breath, and nausea have been observed with rezafungin and are similar to infusion reactions noted with other echinocandins. Symptoms of the infusion reaction may resolve without intervention or with either slowing of the infusion rate or interruption of the infusion.

Mild photosensitivity to ultraviolet (UV) light has been demonstrated in subjects administered rezafungin in a Phase 1 clinical trial. Subjects should use measures to reduce sun and other UV light exposure while on study drug, and for 5 half-lives (approximately 30 days) after the last dose of rezafungin administered.

The Investigator's Brochure should be referenced for additional details or updated information throughout the conduct of the study.

4.8.4 Potential Risks

Echinocandins are typically well tolerated (Eraxis [anidulafungin] Prescribing Information; Mycamine [micafungin sodium] Prescribing Information; Cancidas [caspofungin acetate] Prescribing Information). Potential drug class effects include risk of abnormal liver function tests, hepatitis, and hepatic failure. General and administration site events (infusion related reactions, peripheral edema, rigors, infusion site inflammation, and pyrexia) have been reported for echinocandins (Eraxis [anidulafungin] Prescribing Information; Mycamine [micafungin sodium] Prescribing Information; Cancidas [caspofungin acetate] Prescribing Information). Possible histamine-mediated symptoms have been reported in patients who received rapid infusions of echinocandins, including rash, urticaria, flushing, pruritus, dyspnea, hypotension,

facial swelling, and vasodilation. Anaphylactic-like reactions have been reported with micafungin (Mycamine [micafungin sodium] Prescribing Information). While uncommon, subjects with a history of hypersensitivity to caspofungin or its excipients, including but not limited to anaphylaxis and exfoliative skin disorders, should be excluded from this study.

Based on a fertility study in male rats, there is the potential risk for decreased sperm motility, increased incidences of abnormal sperm morphology, and testicular seminiferous tubular epithelial degeneration. These findings were noted at 2.5-fold the exposure (the estimated steady state plasma level in humans at 200 mg) for Rezafungin subjects in this clinical trial, who will receive 400 mg once weekly loading dose followed by 200 mg once weekly for a total of 2 to 4 doses. The risk to humans is unknown, thus the male contraception requirements in inclusion criterion 4 prohibit unprotected sexual intercourse and sperm donation within the study period and for 90 days thereafter (and a total of 120 days from the last dose of study drug). This risk is included in the informed consent form (ICF).

No major findings occurred in monkeys after 4 weeks of dosing at 30 mg/kg, the highest dose tested. Therefore, for the 3-month toxicity study in monkeys the high dose was increased to 60 mg/kg. Unexpected tremors occurred during the sixth week of dosing that led to lowering the high dose from 60 to 45 mg/kg. Beginning the seventh week of dosing, tremors began at 30 mg/kg, though much less frequently than in the 60/45 mg/kg group. The low-grade tremors at 30 mg/kg did not interfere with the daily function of the animals, and demonstrated reversibility during the recovery period; therefore, they were considered non-adverse. Due to presence of tremors, a detailed neuropathology assessment was performed including whole body perfusions and evaluations of plastic embedded and/or silver stained sections of peripheral nerves/axons and sensory ganglia. Dose-related microscopic findings of Schwann cell phospholipidosis (determined by electron microscopy) were observed in the ganglia (≥3 mg/kg) and peripheral nerves (≥30 mg/kg) and dose-related Schwann cell hyperplasia was observed in the ganglia at ≥30 mg/kg; hyperplasia partially reversed but phospholipidosis was still present at the end of a 4-week recovery period.

A follow-up 3-month investigative monkey study with a 3-month recovery period at 30 mg/kg (NC-154), confirmed the presence of a slight-moderate non-adverse tremoring but no adverse change in nerve conduction. Phospholipidosis in Schwann cells was identified, no change in myelin-to-axon nerve ratios was found, and no hyperplasia of Schwann cells was observed. These findings confirmed lack of neuronal and central nervous system microscopic changes, including no primary axonal degeneration/axonopathy. Tremoring reversed after 1 month of recovery but Schwann cell phospholipidosis was still present at the end of a 3-month recovery period. No other rezafungin-related microscopic changes, including no changes in neurons of the sensory ganglia or structures within the spinal cord or brain, were observed in monkeys. Given the high exposure relative to the Rezafungin for Injection dosing regimen, the risk to study subjects is assessed as low. Exclusions and higher acuity neurological monitoring measures have been included in the protocol to protect patients.

Because there may be unknown and potential risks with administration of Rezafungin for Injection, all subjects will be closely monitored for safety and tolerability by repeated assessment of clinical, vital signs, ECGs, and clinical laboratory safety parameters and reporting of AEs. Additionally, there are AESIs, for which investigators will be trained to monitor closely and thorough investigations will occur (see Section 12.7). Finally, an independent Data and Safety Monitoring Board (DSMB) will review all safety parameters throughout the study.

4.8.5 Mitigation of Risks

Due to findings of toxicity at elevated exposures in nonclinical toxicology studies and AEs noted in clinical studies, special measures have been instituted in this protocol.

For the risk of an infusion reaction typically associated with echinocandins, the option to slow the infusion rate to alleviate symptoms has been included in this protocol. This is listed as an AESI and will entail a thorough assessment if it occurs.

For the risk of photosensitivity with sun exposure, the Sponsor has notified investigators of the findings and added wording to the ICF for subjects to use measures to reduce sun exposure and other types of UV radiation while on study drug, and up to 30 days post dose. This is listed as an AESI and will entail a thorough assessment if it occurs.

For the spermatogenesis findings in the nonclinical studies, all subjects will be informed of the risks in the informed consent and will be prohibited from unprotected sexual intercourse and sperm donation during the conduct of the study and for 90 days thereafter, and at least 120 days from the last dose of study drug.

For the neurologic findings in the 13-week cynomolgus monkey toxicology study, this protocol has enhanced exclusion criteria, physical exam monitoring including periodic neurological exams, and use of an independent DSMB to protect those who are potentially at the greatest risk of developing a neurological AE secondary to rezafungin use.

4.9 Justification for Dosing Regimen

4.9.1 Rezafungin for Injection

A population PK model has been developed for rezafungin (Lakota 2018). Data from a subset of Phase 2 Part A data as well as data from a Phase 1 study was added to update the structural model and evaluate covariate effects. Preliminary data from the modeling was used to set doses for this study.

In brief, these data were best described using a 4-compartment model with 0-order drug input via the IV infusion and first-order, linear elimination. This model fit the observed data with very little bias and excellent precision.

Monte Carlo simulations for 2000 hypothetical patients were conducted to assess the probability of PK/PD target attainment using the weekly free-drug area under the rezafungin

concentration-time curve (*f*AUC) from time 0 to 168 hours (*f*AUC_{0-168}) after each dose for the two dosing regimens tested in Part A; albumin, weight, and sex were bootstrapped (using random sampling and replacement) from the dataset of subjects and plasma protein binding of 97.4% was used.

Nonclinical studies of *C. albicans* infection models in mice have shown that a rezafungin free-drug *f*AUC:MIC is the PK/PD parameter most predictive of efficacy, and *f*AUC_{0-168}:MIC targets have been established in the mouse model for important pathogenic *Candida* spp.

The PK/PD target attainment for the 2 regimens used in Part A (400 mg once weekly for 2 to 4 weeks and a 400 mg loading dose on Week 1 followed by 200 mg once weekly for a total of 2 to 4 weeks) relative to the reported MIC distribution (Pfaller 2020) is shown in Table 4 and Figure 5 for *C. albicans* and in Table 5 and Figure 6 for *C. glabrata*. Both regimens provided adequate PK/PD target attainment throughout 4 weeks of dosing, up to a MIC of 0.5 µg/mL for *C.albicans*, and up to an MIC of 16 µg/mL for *C. glabrata*. The 400 mg once weekly dosing regimen seems to provide some additional benefit in terms of target attainment, improving by 40 to 70% for an MIC = 1, but not until Week 2 or beyond. However, most of the benefits of antifungal treatment occur in the first week of therapy, and the incremental improvement for the 400 mg once weekly dose regimen for Weeks 2 and 3 will likely be of little real clinical benefit in the treatment of candidemia or invasive candidiasis. In fact, in comparison to other echinocandins, the 400 mg loading dose on Week 1, followed by 200 mg once-weekly dose regimen provides a significant advantage in terms of potential to cover pathogens with higher MICs (Table 6).

The data from Part A indicate that both rezafungin regimens were comparable to each other and to caspofungin for efficacy and safety. There were no AEs that seemed to cluster in the 400 mg once weekly group to indicate any concern for increased toxicity with the higher dose. However, the Phase 1 results do indicate that there are infusion reactions that can occur in the higher dose rezafungin regimen that are an echinocandin class effect and are related to the rate of infusion (mg/min). Thus, given there is no apparent clinical benefit to the 400 mg once weekly dose regimen over the regimen with a 400 mg loading dose in Week 1, followed by 200 mg once weekly, and that both options provide at least a three dilution MIC improvement in target attainment over the best of the currently approved echinocandins (Table 6), the regimen of a 400 mg loading dose in Week 1, followed by 200 mg once weekly for a total of 2 to 4 doses can be selected for safety and efficacy in the treatment of candidemia and/or invasive candidiasis.

Table 4: Predicted Pharmacokinetic-Pharmacodynamic Target Attainment for Rezafungin Regimens, Stratified by Week and Minimal Inhibitory Concentration, Against *Candida albicans*

Regimen [a]	Week	MIC (mg/L) [b]					
		0.06	0.12	0.25	0.5	1	2
400/400	1	100	100	100	98.8	35.1	0.05
	2	100	100	100	99.95	73.4	2.45
	3	100	100	100	100	84.3	7.1
	4	100	100	100	100	88.3	10.75
400/200	1	100	100	100	98.8	35.1	0.05
	2	100	100	100	92.55	14.95	0
	3	100	100	100	91.05	14.45	0
	4	100	100	100	90.55	15	

Abbreviation: MIC = minimal inhibitory concentration; PD = pharmacodynamic; PK = pharmacokinetic.

a. Regimens defined by the weekly dose (e.g., 400/200 represents 400 mg for the first dose, followed by 200 mg IV once weekly).

b. Shaded cells indicate PK/PD target attainment above 90%.

Figure 5: Predicted Pharmacokinetic-Pharmacodynamic Target Attainment for Rezafungin Regimens, Against *Candida albicans* Relative to MIC Distribution

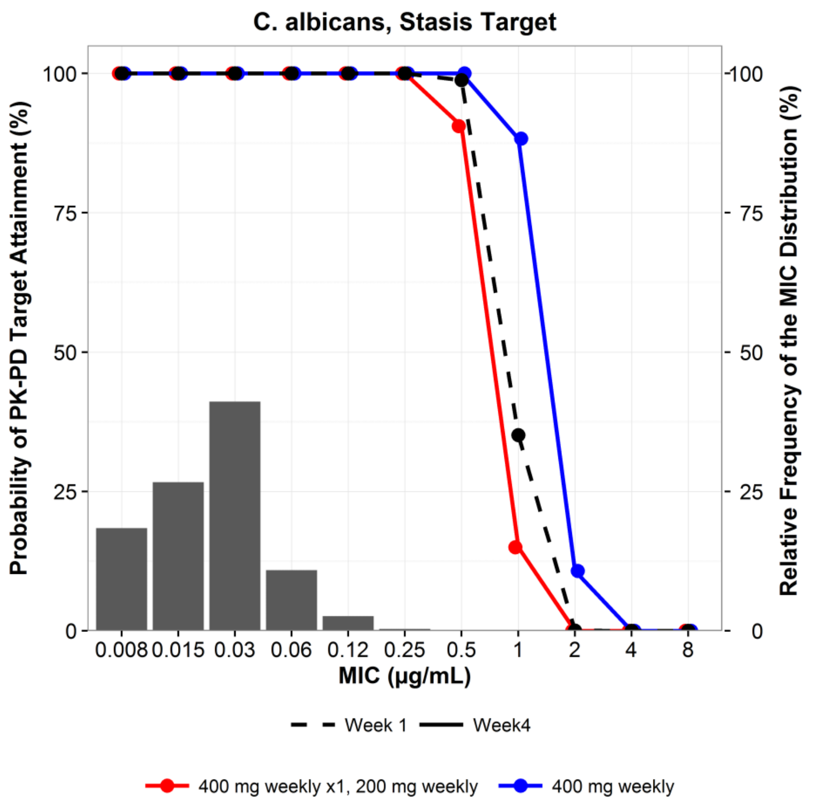

Table 5: Predicted Pharmacokinetic-Pharmacodynamic Target Attainment for Rezafungin Regimens, Stratified by Week and Minimal Inhibitory Concentration, Against *Candida glabrata*

Regimen [a]	Week	MIC (mg/L) [b]					
		0.12	0.5	2	4	8	16
400/400	1	100	100	100	100	100	100
	2	100	100	100	100	100	100
	3	100	100	100	100	100	100
	4	100	100	100	100	100	100
400/200	1	100	100	100	100	100	100
	2	100	100	100	100	100	100
	3	100	100	100	100	100	100
	4	100	100	100	100	100	98.65

Abbreviation: MIC = minimal inhibitory concentration; PD = pharmacodynamic; PK = pharmacokinetic.
[a.] Regimens defined by the weekly dose (e.g., 400/200 represents 400 mg for the first dose, followed by 200 mg once weekly).
Shaded cells indicate PK/PD target attainment above 90%.

Figure 6: Predicted Pharmacokinetic-Pharmacodynamic Target Attainment for Rezafungin Regimens, Against *Candida glabrata* Relative to MIC Distribution

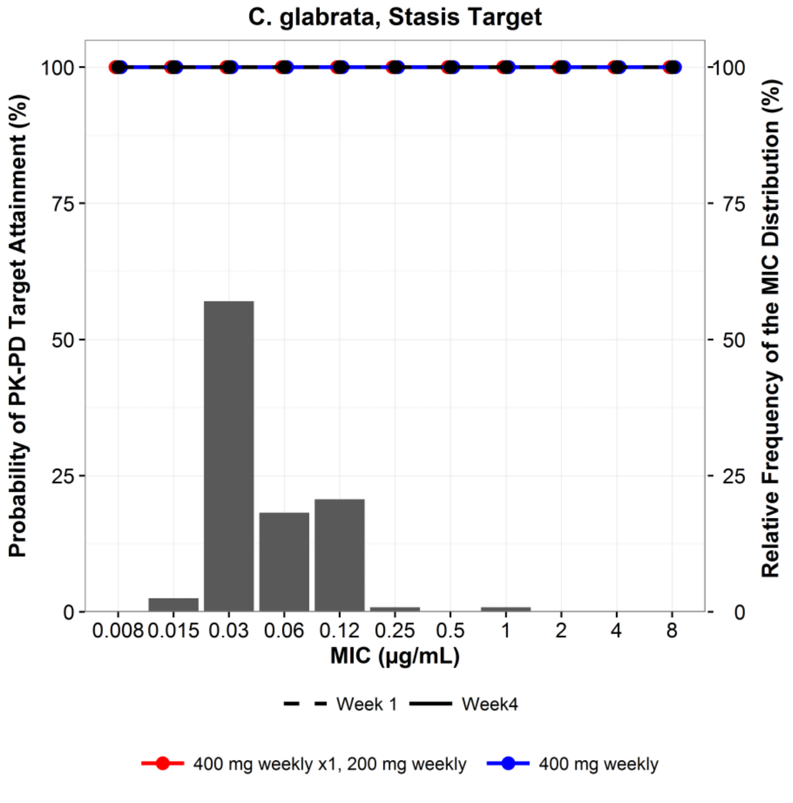

Table 6: Percent Probabilities of Pharmacokinetic-Pharmacodynamic Target Attainment by Anidulafungin, Caspofungin, Micafungin, and Rezafungin against *Candida* spp.

MIC (µg/mL)	Anidulafungin		Caspofungin		Micafungin		Rezafungin*	
	C. albicans	*C. glabrata*	*C. albicans*	*C. glabrata*	*C. albicans*	*C. glabrata*	*C. albicans*	*C. glabrata*
0.008	100[a]	100[a]	100[a]	100[a]	99.4[a]	100[a]	100[a]	100[a]
0.015	99.1[a]	100[a]	100[a]	100[a]	71.2	100[a]	100[a]	100[a]
0.03	**52.7**[b]	99.2[a]	**100**[a,b]	100[a]	**10.1**[b]	**97.5**[a,b]	100[a,c]	100[a,c]
0.06	0.90	54.3	97.9[a]	**100**[a,b]	0.10	49.9[c]	**100**[a,b]	100[a]
0.12	0	**0.95**[b,c]	76.7	100[a,c]	0	3.4	100[a]	**100**[a,b]
0.25	0[c]	0	35.7[c]	100[a]	0[c]	0	100[a]	100[a]
0.5	0	0	12.1	97.0[a]	0	0	90.55[a]	100[a]
1	0	0	4.4	73.2	0	0	15	100[a]
2	0	0	1.35	33.9	0	0	0	100[a]
4	0	0	0.25	11.3	0	0	0	100[a]
8	0	0	0.05	4.35	0	0	0	100[a]

Abbreviations: *C.* = *Candida*; MIC = minimal inhibitory concentration; MIC_{90} = minimal inhibitory concentration required to inhibit the growth of 90% of the isolates; PD = pharmacodynamic; PK = pharmacokinetic.

a Shaded cells indicate PK-PD target attainment values ≥90%.

b Bolded values represent the susceptible breakpoints for each agent against the respective *Candida* spp.

c Cells with bolded outlines represent the MIC_{90} values for each agent against the respective *Candida* spp.

* 400/200/200/200 at wk 4

Note: MIC and target attainment data for other echinocandins (Bader 2016).

4.9.2 Caspofungin with Option for Oral Step-Down Regimen of Fluconazole

Treatment regimens with caspofungin and fluconazole are consistent with standard of care regimens in approved labeling (Cancidas [caspofungin acetate] Prescribing Information; Diflucan [fluconazole] tablets Prescribing Information).

4.10 Population to be Studied

Adult subjects with candidemia and/or invasive candidiasis.

4.11 Rationale for Use of Rezafungin for Injection in Candidemia and Invasive Candidiasis

Candidemia is a fungal bloodstream infection usually affecting patients who are already ill with other comorbidities. Invasive candidiasis defines a number of different organ-specific infections with *Candida* spp. Candidemia and invasive candidiasis may present without the other syndrome or may exist together as part of the same pathophysiology of disseminated candidiasis. Risk factors for candidemia and invasive candidiasis include recent surgery, organ and bone marrow transplants, broad-spectrum antibiotic use, receipt of total parenteral nutrition, end stage renal disease, indwelling vascular catheters or other devices, etc. While candidemia is often associated with other forms of invasive candidiasis, including intra-abdominal abscess, peritonitis, or pleuritic involvement, candidemia without other overt organ involvement is the most common form of invasive candidiasis diagnosed. A recent study by the ESCMID Fungal Infection Study Group reported the incidence of candidemia as 6.9 per 1000 intensive care unit (ICU) patients with mortality rates between 20% and 49% (Kett 2011; Gudlaugsson 2003; Arendrup 2011). Candidemia and invasive candidiasis are serious and potentially life-threatening infectious complications that require aggressive and timely antifungal management.

The current IDSA and ESCMID guidelines recommend echinocandins as first-line therapy for candidemia and most forms of invasive candidiasis (Pappas 2016; Cornely 2012). There are currently 3 commercially authorized echinocandins in the US and the European Union: caspofungin, micafungin, and anidulafungin. These echinocandins are administered once daily via IV infusion and share similar PK parameters and safety profiles. Rezafungin is a novel echinocandin derived from another echinocandin (anidulafungin). Rezafungin has a more stable molecular structure compared to the other echinocandins, which results in a reduction in the formation of toxic metabolites and an improvement in the duration of exposure to active drug. This stability further translates into a potentially improved safety profile as demonstrated in both nonclinical and clinical studies, as well as a unique PK profile that allows for high dose, front-loaded exposures for improved fungal killing and once weekly IV dosing.

Both in vitro and in vivo data have demonstrated the potency and efficacy of rezafungin against *Candida* and disseminated candidiasis (Section 4.1 and Investigator's Brochure). In all nonclinical efficacy studies performed for Rezafungin in neutropenic disseminated candidiasis, rezafungin has performed as well as, or better than, currently approved echinocandin comparators. Furthermore, nonclinical data indicate the exposure of rezafungin is substantially higher in infected tissues compared to other echinocandins, indicating the potential to improve outcomes in invasive candidiasis (Zhao 2017). The completed Part A of the Phase 2 study demonstrated efficacy for Rezafungin for Injection comparable to standard of care at Day 14 for all endpoints. The Rezafungin for Injection regimen starting with a 400 mg once weekly loading dose followed by 200 mg once weekly for a total of 2 to 4 doses, had robust results for the primary endpoint of overall success compared with standard of care (Rezafungin for Injection, 71.0%; caspofungin, 64.3%). Additionally, the Phase 2 results for Part A also indicated that Rezafungin for Injection appears to be well-tolerated at 400 mg once weekly and at 400 mg

loading dose Week 1 followed by 200 mg once weekly. In PK and PD models, the proposed Rezafungin for Injection dosing regimens for this Phase 3 study demonstrate 100% target attainment of wild-type *C. albicans* and *C. glabrata* (*C. albicans* data is presented in Section 4.9). The duration of antifungal therapy in standard practice depends on the time it takes to clear the *Candida* from infected blood or tissues, and thus patients with invasive candidiasis often require a longer duration of therapy than patients with candidemia alone. To that end, all subjects in this study must be administered study drug for at least 14 days followed by an optional third or fourth week of treatment if clinically indicated.

This Phase 3 study is intended to confirm the efficacy and safety of Rezafungin for Injection administered at 400 mg loading dose in Week 1 followed by 200 mg once weekly for the treatment of patients with candidemia and/or invasive candidiasis.

4.12 Statement of Compliance

This study will be conducted in compliance with the protocol, Good Clinical Practice (GCP), the ethical principles of the Declaration of Helsinki, and applicable regulatory and Institutional Review Board (IRB) or Independent Ethics Committee (IEC) requirements.

5.0 STUDY OBJECTIVES

The primary objectives of this study are to:

- Demonstrate that Rezafungin for Injection is non-inferior to caspofungin for all-cause mortality (ACM) at Day 30 (-2 days) in the modified intent-to-treat (mITT) population (US Food and Drug Administration [FDA] primary objective)
- Demonstrate that Rezafungin for Injection is non-inferior to caspofungin for global cure (clinical cure as assessed by the Investigator, radiological cure [for qualifying invasive candidiasis subjects], and mycological eradication, as confirmed by the Data Review Committee [DRC]) at Day 14 (±1 day) in the mITT population (European Medicines Agency [EMA] primary objective)

The secondary objectives of this study are to:

- Compare global cure (clinical cure as assessed by the Investigator, radiological cure [for qualifying invasive candidiasis subjects], and mycological eradication, as confirmed by the DRC) for subjects receiving Rezafungin for Injection and caspofungin at Day 5, Day 30 (-2 days), End of Treatment (EOT) (≤2 days of last dose), and Follow-up (Days 52–59) in the mITT population
- Compare mycological eradication for subjects receiving Rezafungin for Injection and caspofungin at Day 5, Day 14 (±1 day), Day 30 (-2 days), EOT (≤2 days of last dose), and Follow-up (Days 52–59) in the mITT population
- Compare clinical cure as assessed by the Investigator for subjects receiving Rezafungin for Injection and caspofungin at Day 5, Day 14 (±1 day), Day 30 (-2 days), EOT (≤2 days of last dose), and Follow-up (Days 52–59) in the mITT population
- Compare radiological cure for invasive candidiasis subjects receiving Rezafungin for Injection and caspofungin at Day 5, Day 14 (±1 day), Day 30 (-2 days), EOT (≤2 days of last dose), and Follow-up (Days 52–59) in the mITT population
- Compare the safety and tolerability for subjects receiving Rezafungin for Injection and caspofungin in the safety population
- Evaluate the PKs of Rezafungin for Injection

An exploratory objective of this study is to:

- Compare resolution of systemic signs attributable to candidemia and/or invasive candidiasis for subjects receiving Rezafungin for Injection and caspofungin at Day 5, Day 14 (±1 day), Day 30 (-2 days) and Follow-up Days (52–59) in the mITT population

6.0 STUDY DESIGN

6.1 Description of the Study

The study design figure is provided in Figure 1. The Schedule of Assessments and Procedures for the Required Treatment, EOT, and Follow-up Periods is presented in Table 1 and the Schedule of Assessments and Procedures for the Optional Treatment Period is presented in Table 2.

This is a Phase 3, multicenter, prospective, randomized, double-blind, efficacy and safety study of Rezafungin for Injection (IV) versus a comparator regimen of caspofungin (IV) followed by optional oral fluconazole step-down therapy in subjects with candidemia and/or invasive candidiasis. Subjects will be randomly assigned (1:1 ratio) to receive either Rezafungin for Injection or caspofungin. After ≥3 days IV treatment (or the minimum duration of IV therapy advised by the site's national/regional/local guidelines, whichever is greater), subjects may be switched to oral step-down therapy by the Investigator if they meet the oral step-down therapy criteria.

6.1.1 Subjects Randomized to Rezafungin for Injection

Subjects randomized to Rezafungin for Injection will receive a 400 mg loading dose in Week 1, followed by 200 mg once weekly, for a total of 2 to 4 doses. To maintain the blind, subjects will also receive daily placebo to match for caspofungin IV, which will be administered on Days 2–7, Days 9–14, and during the optional dosing period on Days 16–21 and Days 23–28, or will receive daily placebo for oral step-down therapy (first eligibility on Day 4 or later as advised by a site's national/regional/local guidelines), which will be administered every day including rezafungin infusion days (see Figure 1 and Figure 2).

6.1.2 Subjects Randomized to Caspofungin

Subjects randomized to caspofungin will receive a total treatment of ≥14 days beginning with a single caspofungin 70 mg IV loading dose on Day 1, followed by caspofungin 50 mg IV once daily with the option to continue treatment ≤28 days. After ≥3 days (or the minimum duration of IV therapy advised by the site's national/regional/local guidelines, whichever is greater) of caspofungin treatment, subjects who meet the oral step-down therapy eligibility criteria (see Section 8.2.3.3) may be switched to oral fluconazole at a dose of 6 mg/kg administered once daily (rounded to the nearest 200 mg increment) with a maximum daily dose of 800 mg (e.g., a subject weighing 73 kg would receive fluconazole 400 mg dose [2 capsules of 200 mg each] based on a 6 mg/kg target dose [73 kg × 6 mg/kg = 438 mg]. NOTE: Refer to dosing section for alternative regimens of fluconazole in subjects with renal impairment in Section 8.2.3.5). To maintain the blind, subjects in the caspofungin treatment group who have switched to oral step-down therapy will receive matching IV placebo for Rezafungin for Injection once weekly on the days of scheduled Rezafungin for Injection doses until study drug is stopped (Day 8; Day 15 [if applicable] and Day 22 [if applicable]) (see Figure 1 and Figure 2).

6.1.3 End of Treatment Visit and Follow-up Visits

Subjects will complete an EOT visit within 2 calendar days after the last dose of study drug, and a Follow-up visit within the Day 52–59 period. Subjects who stop study drug prior to Day 22 AND are considered clinical failures (i.e., require a change in antifungal therapy to treat candidemia and/or invasive candidiasis) may have their Follow-up visit earlier, but the visit must still be ≥30 days following the last once weekly infusion of Rezafungin for Injection/placebo (i.e., Day 1, Day 8, Day 15, or Day 22).

6.1.4 Mycological Diagnosis

Mycological diagnosis of candidemia and/or invasive candidiasis sufficient for inclusion in the study will be established by one of the following test results from a blood sample or normally sterile site collected ≤4 days (96 hours) before randomization:

- ≥1 blood culture positive for yeast or *Candida*
- sponsor-approved rapid in vitro diagnostic (IVD) (e.g., T2Candida Panel, Septifast) test positive for *Candida* spp.
- a positive gram stain (or other method of direct microscopy) for yeast or positive culture for *Candida* spp. from a specimen obtained from a normally sterile site

Note: If the positive blood culture used to qualify the subject for the study is drawn >12 hours prior to randomization, an additional set of blood cultures must be obtained ≤12 hours before randomization to determine which subjects are still candidemic at enrollment. Results of the blood cultures obtained ≤12 hours before randomization are not required prior to randomization.

The dose and duration of any prior antifungal treatment received ≤4 weeks before randomization will be recorded at Screening.

For diagnosis of invasive candidiasis, cultures should be obtained from purulent fluid or necrotic tissues from normally sterile sites. Drainage tubes following invasive procedures may only be used to acquire fluid for culture for up to 24 hours following placement of the tube (after 24 hours tubes could be colonized with non-infecting pathogens). While potentially indicative of disease, a urine culture alone may not be used as the qualifying culture for entry into the study, though the presence of a positive urine culture is not a reason to exclude a patient from participation. Additionally, a respiratory tract specimen would not be considered sufficient to prove invasive candidiasis as these specimens are rarely sterile and pulmonary infection with *Candida* spp. is extremely rare.

Every reasonable effort must be made for source control of the *Candida* infection to properly evaluate the efficacy of study drug. Central catheters should be removed within 48 hours after diagnosis with candidemia, consistent with IDSA and ESCMID guidelines. For subjects with invasive candidiasis, surgical drainage or debridement of potentially infected fluid collections or necrotic tissues should be performed.

6.1.5 Retinal Examination

A retinal examination for evidence of a *Candida* eye infection, including endophthalmitis or chorioretinitis, should be performed on all subjects with candidemia during Screening when possible, although the exam may occur as late as Day 7. Retinal examination is standard of care for subjects with candidemia and should be performed by an ophthalmologist, when available. The retinal examination should be repeated in subjects who were negative at baseline if the subject develops visual signs or symptoms of a *Candida* eye infection during the study. Additionally, any neutropenic subjects who recover their neutrophil counts during the study should have an additional retinal examination. Subjects who were negative at baseline and diagnosed with a *Candida* eye infection after initiating study drug must discontinue study drug (see Section 7.4.2) but may continue in the study at the Investigator's discretion; all subsequent antifungal drugs, interventions, and outcomes for endophthalmitis or chorioretinitis will be recorded.

6.1.6 Study Day Definition

Study Day 1 is defined as the first day of study drug administration. Subsequent study days are counted as the number of consecutive calendar days thereafter.

6.1.7 Efficacy Assessments

Efficacy assessments for all subjects occur on Day 5, Day 14 (±1 day), Day 30 (-2 days), EOT (≤2 days of last dose), and the Follow-up visit (Days 52–59). Clinical cure, radiological cure (for qualifying invasive candidiasis subjects), and mycological eradication will be assessed on all efficacy assessment days. Survival status will be assessed at Day 30 (-2 days) and the Follow-up visit (Days 52–59). Subjects who complete Day 30 assessments on Day 28 or Day 29 (within the -2 days period) without further information thereafter will be considered alive at Day 30. For mycological response, the location of *Candida* infection determines how eradication will be demonstrated:

a. **Subjects with ≥1 blood culture positive for yeast or *Candida***

   Blood cultures for efficacy following the first dose of study drug will be performed until the first negative blood culture result for *Candida* spp. with no subsequent positive culture (in cases when one or more samples are drawn and cultured after the first negative culture is available). Blood samples for cultures should be drawn daily when possible although may be drawn every other day until blood cultures are negative. Generally, blood cultures should involve samples from 2 separate draws with ≥1 draw from a peripheral vein without an IV catheter.

b. **Subjects with a positive culture for *Candida* spp. from a normally sterile site**

   Note: only applies when infected sites are accessible; fluid samples from drainage tubes may only be collected for up to 24 hours following placement of the tube. Culture specimens for efficacy following the first dose of study drug should be

collected from all normally sterile sites of baseline infection at least every other day until the first negative culture result for *Candida* spp. with no subsequent positive culture (in cases when one or more samples are drawn and cultured after the first negative culture is available). If access to the infected site is not possible, then mycological eradication will be determined based on clinical outcome (see Table 8). Any time there is a culture obtained from a normally sterile site, there should be a surgical procedure recorded in the Medical History or in Concomitant Procedures (depending upon the study interval during which the sample was obtained).

6.1.8 Safety Assessments

Safety monitoring data is collected throughout the study. Adverse events are collected from the time the informed consent document is signed through the Follow-up visit (Days 52–59). Vital signs will be measured at each study visit. A physical examination will be performed at the Screening, Day 5, Day 14 (±1 day), Day 30 (-2 days), EOT (≤2 days of last dose), and Follow-up (Days 52–59) visits. Subjects will be assessed for signs and symptoms of tremor, ataxia, and peripheral neuropathy using a neurological exam at least at Screening and at EOT. More frequent neurological exams may be performed as directed by subject symptoms. Hematology and chemistry laboratory tests will be performed at the Screening, Day 1, Day 2, Day 4, Day 8 (±1 day), Day 14 (±1 day), Day 30 (-2 days), EOT (≤2 days of last dose), and Follow-up visits (Days 52–59). Urinalysis will be performed at the Screening and EOT (≤2 days of last dose) visits. Coagulation laboratory tests (Prothrombin Time or International Normalized Ratio [PT/INR]) will be performed at the Screening visit. An ECG will be performed at the Screening visit and on Day 1 at any time following administration of study drug (i.e., post-end IV infusion). An independent external DSMB will review safety data when approximately 50% of subjects are randomized in the study and as required for AESIs as provided in the DSMB Charter.

6.1.9 PK Sampling

Blood samples for PK analysis must be collected from the OPPOSITE arm of the infusion or through an arterial line during the infusion; after the infusion is complete, opposite arm or arterial line draws are preferred, but not mandatory. Blood samples will be collected as follows:

- Day 1: within 10 minutes before the end of infusion, and one sample taken any time between end of infusion and 12 hours after the end of infusion
- Days 2, 3, 4, or 5: One sample taken at any time on one day only
- Day 8: Pre-dose
- Day 14 (for subjects not receiving a Day 15 dose): One sample taken at any time
- Day 15: Pre-dose (if applicable)
- Day 22: Pre-dose (if applicable)

For subjects completing treatment on Day 14, a PK sample should be drawn at the same time as hematology and chemistry blood draws (i.e., random PK draw). **Note**: Pre-dose samples should be collected ≤30 minutes prior to the start of the infusion. For subjects receiving hemodialysis, an attempt should be made to obtain PK samples directly following hemodialysis if still within the required time window.

Blood samples will be collected from all subjects receiving study drug, although only PK samples from subjects receiving Rezafungin for Injection will be analyzed. PK sample analysis will be performed by an independent, central bioanalytical laboratory using a validated assay.

6.2 Number of Subjects

Approximately 218 subjects will be randomized to treatment (1:1, Rezafungin for Injection to caspofungin). The target of 218 subjects assumes approximately 85% of randomized subjects will be evaluable for the mITT population. Thus, 184 subjects will be in the mITT population (92 subjects in each treatment group). If the evaluability rate for the mITT population is higher than 85%, enrollment will be stopped when at least 184 randomized subjects have met the criteria to be included in the mITT population.

6.3 Measures Taken to Minimize Bias

Treatment assignment will be blinded to all blinded clinical site and sponsor personnel (double-blind) until the study database is locked. Unblinded personnel includes the site pharmacist preparing the infusions and the unblinded monitor to ensure study drug accountability and assignment throughout the trial as well as the Sponsor's unblinded team. Blinding is maintained with a double-dummy design wherein all subjects receive daily infusions until switched to oral step-down therapy. Subjects switched to oral step-down therapy receive daily oral therapy and weekly infusions.

6.4 Expected Duration of Subject Participation

Maximum study duration for an individual subject is 63 days, including Screening (≤4 days, 96 hours), the Required Treatment Period (Day 1 to Day 14 [14 days]), the Optional Extended Treatment Period (Day 15 to Day 28), and Follow-up (Days 52–59).

6.5 Method of Treatment Assignment and Blinding

After informed consent has been obtained, subjects will be screened for study eligibility before randomization.

Subjects will be randomly assigned (1:1 ratio) to receive either Rezafungin for Injection or caspofungin. Randomization will be stratified based on diagnosis (candidemia only; invasive candidiasis) and by Acute Physiology and Chronic Health Evaluation (APACHE II) score/ absolute neutrophil count (ANC) (APACHE II score ≥20 OR ANC <500 cells/µL; APACHE II score <20 AND ANC ≥500 cells/µL) at Screening. If a subject has a positive result

for both blood culture/rapid IVD and culture specimen obtained from a normally sterile site, the subject will be randomized within the invasive candidiasis stratum.

The study site's pharmacist (or pharmacist designee) will obtain the study drug assignment via an Interactive Response Technology (IRT). Randomized subjects will be assigned the treatment corresponding to the next available randomization number from the computer-generated randomization schedule. A subject is considered randomized when a randomization transaction is recorded in the IRT.

All study personnel, including the Sponsor, Investigator, and site personnel involved in study conduct, and subjects will remain blinded to study medication assignment until the study is completed and the database is locked with the exception of the pharmacy monitor, unblinded project manager, clinical supplies, document manager, and quality (per the Sponsor Blinding Plan) who may be unblinded to study medication at any time during study conduct. The DSMB will be unblinded to safety data for periodic safety reviews as defined in the DSMB Charter. The Pharmacy Monitor will monitor study drug preparation and accountability during the study and cases in which unblinding is required due to a safety or tolerability issue. To maintain study blinding, study drug preparation will be performed by an unblinded site pharmacist (or qualified unblinded personnel at the study site not involved with study procedures or evaluations).

Instructions for study drug preparation and dosing are outlined in the Pharmacy Manual provided separately to the site. To maintain the blind, oral study drug pills must never be crushed. In the case of a medical emergency requiring the Investigator to know the identity of the study drug, the Investigator will follow the procedures outlined in Section 8.4.

7.0 SELECTION, DISCONTINUATION, AND WITHDRAWAL OF SUBJECTS

7.1 Subject Inclusion Criteria

Subjects must meet ALL the following inclusion criteria to qualify for the study:

1. Willing and able to provide written informed consent. If the subject is unable to consent for himself/herself, a legally acceptable representative (i.e., acceptable to International Council on Harmonisation [ICH] and local law, as applicable) must provide informed consent on his/her behalf.
2. Males or females ≥18 years of age.
3. Established mycological diagnosis of candidemia and/or invasive candidiasis from a sample taken ≤4 days (96 hours) before randomization defined as:
    a. ≥1 blood culture positive for yeast or *Candida*

       OR
    b. Positive test for *Candida* from a Sponsor-approved rapid IVD

       OR
    c. Positive gram stain (or other method of direct microscopy) for yeast or positive culture for *Candida* spp. from a specimen obtained from a normally sterile site.
4. Presence of one or more systemic signs attributable to candidemia or invasive candidiasis (e.g., fever, hypothermia, hypotension, tachycardia, tachypnea, local signs of inflammation) appearing from ≤12 hours prior to the qualifying positive culture through time of randomization.
5. Willing to initiate or continue medical treatment to cure infections, including receipt of antibiotics and surgical procedures, if required. Patients receiving only medications and measures for comfort and not cure should not be enrolled.
6. Female subjects of childbearing potential (all female subjects between 18 years and <2 years post-menopausal unless surgically sterile) must agree to and comply with using one barrier method (e.g., female condom with spermicide) plus one other highly effective method of birth control (e.g., oral contraceptive, implant, injectable, indwelling intrauterine device, vasectomized partner), or sexual abstinence (only possible if it corresponds to the subject's usual lifestyle) while participating in this study and at least 30 days from the last dose of study drug. Male subjects must be vasectomized, abstain from sexual intercourse, or agree to use barrier contraception (condom with spermicide), and also agree not to donate sperm while participating in the study and for 90 days thereafter (and at least 120 days from the last dose of study drug).
7. For candidemia only subjects, drawing of a set of blood cultures within 12 hours prior to randomization in the study. The result of these blood cultures is not required for inclusion in the study.

7.1.1 Rapid In Vitro Diagnostics for Candidemia and Invasive Candidiasis

The addition of rapid IVD for the identification of subjects with candidemia and/or invasive candidiasis is being employed to reduce the amount of prior empiric antifungal therapy being administered to potential study subjects. Rapid IVDs for *Candida* spp. used for inclusion in this study must be approved by the Sponsor prior to use in the study. Currently approved rapid IVDs include the T2Candida Panel (T2 Biosystems) and the Septifast polymerase chain reaction (PCR) test (Roche). Subjects may be enrolled based on a positive result from a Sponsor-approved rapid IVD, but a culture from blood or normally sterile tissue or fluid that is positive for *Candida* spp. will still be the required objective measure for candidemia or invasive candidiasis in the study analyses for efficacy.

Potential subjects with 1 or more risk factors for candidemia and invasive candidiasis should have a Sponsor-approved rapid IVD for *Candida* spp. performed only if the site is drawing a blood sample for blood culture and if empiric antifungal therapy for candidiasis has been administered or will be administered within the subsequent day. Risk factors for candidemia and invasive candidiasis include (but are not limited to):

- Immunosuppression
- Central venous catheter
- Recent abdominal surgery
- Pancreatitis
- End stage renal disease
- Current daily administration of total parenteral nutrition
- Systemic inflammatory response syndrome while on broad spectrum antibiotics

Sites may choose to perform a sponsor-approved rapid IVD in potential subjects without the listed risk factors for candidemia and invasive candidiasis, but the potential subject still must have a blood culture drawn and empiric antifungal therapy administered. A call to the Medical Monitor is recommended for any questions about risk factors for candidemia and the appropriate subjects on which to perform a rapid IVD for *Candida* spp.

7.1.2 Systemic Signs of Candidemia and Invasive Candidiasis

It is a requirement that at least 1 systemic sign attributable to candidemia and/or invasive candidiasis be present at Screening for the subject to be eligible for enrollment. The Screening period for assessing systemic signs for inclusion in the study may include the 12 hours prior to the collection of the qualifying positive culture when systemic signs of infection were the reason for specimen collection (blood culture or fluid from a sterile site culture or qualifying rapid IVD [e.g., T2Candida Panel]) through study randomization. The possible signs of infection that might be attributable to candidemia and/or invasive candidiasis at baseline include fever, hypothermia,

hypotension, tachycardia, tachypnea, and local signs of inflammation (erythema, edema, heat, and pain at the site of the qualifying blood culture).

For the listed systemic signs of candidemia, the qualifying parameters are provided below:

**Systemic Sign of Candidemia**	**Qualifying Parameters**
Fever	Oral or axillary temperature ≥38°C (100.4°F) or a tympanic, temporal, rectal, or core body temperature ≥38.3°C (101°F)
Hypothermia	Tympanic, temporal, rectal, or core body temperature ≤35°C (95.2°F)
Hypotension	Systolic blood pressure <90 mm Hg or mean arterial pressure <70 mm Hg with a normovolemic or hypervolemic status
Tachycardia	Heart rate >100 beats per minute with a normovolemic or hypervolemic status
Tachypnea	Respiratory rate >20 breaths per minute
Local Signs of Inflammation	Erythema (rubor), edema (tumor), heat (calor), and pain (dolor) at the site of infection

7.2 Subject Exclusion Criteria

Subjects must NOT meet any of the following exclusion criteria to qualify for the study:

1. Any of the following forms of invasive candidiasis at baseline:
    a. Septic arthritis in a prosthetic joint (septic arthritis in a native joint is allowed)
    b. Osteomyelitis
    c. Endocarditis or myocarditis
    d. Meningitis, endophthalmitis, chorioretinitis, or any central nervous system infection
    e. Chronic disseminated candidiasis
    f. Urinary tract candidiasis due to ascending *Candida* infection secondary to obstruction or surgical instrumentation of the urinary tract
2. Received systemic treatment with an antifungal agent at approved doses for treatment of candidemia for >48 hours (e.g., >2 doses of a once daily antifungal agent or >4 doses of a twice daily antifungal agent) ≤4 days (96 hours) before randomization.
    a. Exception: Receipt of antifungal therapy to which any *Candida* spp. isolated in culture is not susceptible (see Appendix 1)
3. Alanine aminotransferase or aspartate aminotransferase levels >10-fold the upper limit of normal (ULN).
4. Severe hepatic impairment in subjects with a history of chronic cirrhosis (Child-Pugh score >9).

5. Presence of an indwelling vascular catheter or device that cannot be removed or an abscess that cannot be drained and is likely to be the source of candidemia or invasive candidiasis.
6. Known hypersensitivity to Rezafungin for Injection, caspofungin, any echinocandin, or to any of their excipients, including but not limited to hereditary sugar disorders (e.g., fructose intolerance, sucrose-isomaltase insufficiency), anaphylaxis, or echinocandin-induced exfoliative skin disorders (e.g., Stevens-Johnson syndrome or toxic epidermal necrolysis).
7. Meets National Cancer Institute Common Terminology Criteria for Adverse Events (CTCAE) version 5.0, criteria for ataxia, tremor, motor neuropathy, or sensory neuropathy of Grade 2 or higher.
8. History of severe ataxia, tremor, or neuropathy or a diagnosis of multiple sclerosis or a movement disorder (including Parkinson's Disease or Huntington's Disease).
9. Planned or ongoing therapy at Screening with a known severe neurotoxic medication (see Appendix 9) or with a known moderate neurotoxic medication (see Appendix 9) in a patient with ataxia, tremor, motor neuropathy, or sensory neuropathy of CTCAE version 5.0 Grade 1 or higher.
10. Previous participation in this or any previous rezafungin study.
11. Current participation in another interventional treatment trial with an investigational agent.
12. Recent use of an investigational medicinal product within 28 days of the first dose of study drug or presence of an investigational device at the time of Screening.
13. Pregnant or lactating females.
14. The Principal Investigator (PI) is of the opinion the subject should not participate in the study.

7.3 Requalification for Entry

Subjects not fulfilling the entry criteria and not randomized may be rescreened for participation if their eligibility characteristics have changed.

7.4 Subject Withdrawal Criteria

7.4.1 Withdrawal from Study Protocol

Subjects may withdraw consent to participate in this study at any time without penalty or loss of benefits to which the subject is otherwise entitled. Subjects on study drug who wish to withdraw completely from this clinical study should be encouraged to complete the assessments at EOT. Every reasonable effort should be made to determine the reason a subject withdraws prematurely, and this information should be recorded on the appropriate page(s) of the electronic case report form (eCRF).

Subjects may be withdrawn from the study for any of the following reasons:

- Subject unable or unwilling to continue
- Subject elects to withdraw informed consent
- AE (whether related to study drug or not) that precludes further participation in the study in the judgment of the PI and/or Sponsor
- Protocol non-compliance
- Subject lost to follow up
- The PI considers that it is in the subject's best interest not to continue participation in the study

Subjects withdrawn from the study may refuse to participate in the Follow-up visit; however, every effort should be made to collect survival data and safety assessments on Day 30 (-2 days) for all subjects who withdraw for reasons other than withdrawal of informed consent. Following enrollment, subjects diagnosed with invasive candidiasis involving one or more organ systems and normally excluded from study participation (Exclusion Criterion 1, Section 7.2) do not need to exit the study. Documentation of the disease should be placed in the eCRF along with any further interventions related to the disease.

7.4.2 Early Discontinuation from Study Drug Administration

For subjects who prematurely discontinue study drug (i.e., before the anticipated full course of study drug therapy required for effective treatment of candidemia and/or invasive candidiasis), EOT assessments should be performed on the day of discontinuation. For premature study drug discontinuation, Day 14 efficacy assessments should also be performed at EOT, except when the subject is believed to be a clinical cure, then the efficacy assessments should still be performed at the Day 14 visit to ensure there is no relapse between EOT and Day 14. All safety assessments and assessment of mortality should be done for subjects who discontinue study drug early. The reason for premature discontinuation of study drug administration should be recorded on the appropriate page(s) of the eCRF. Reasons for withdrawal from study drug may include, but are not limited to:

- Cultures negative for *Candida* spp.
    - Blood culture is positive for yeast at enrollment, but the final local blood culture results conclude the yeast is not *Candida* spp.
    - Subject is enrolled based on a rapid IVD test positive for *Candida* spp., but final local blood culture results from Screening or tissue/fluid culture results from a normally sterile site are negative for *Candida* spp. (withdrawal not required but based on the Investigator's discretion)

- Safety
  - Subject unable or unwilling to continue or informed consent withdrawn
  - Occurrence of an AE that, in the opinion of the Investigator, warrants the subject's permanent discontinuation from study drug therapy. This includes development of a clinically significant laboratory abnormality that requires discontinuation from study drug (IV and IV/oral) therapy. In the event of discontinuation from study drug therapy due to the occurrence of an AE, the study site should notify the Medical Monitor as soon as possible
  - Suspected or confirmed pregnancy or nursing during the study drug administration period
- Excluded types of invasive candidiasis

  Echinocandins are not appropriate therapy for *Candida* infections of the eye or central nervous system due to low drug distribution to these sites (Pappas 2016; Cornely 2012). A retinal examination for evidence of a *Candida* eye infection, including endophthalmitis or chorioretinitis, should be performed in all subjects with candidemia during Screening when possible, although may occur as late as Day 7 only on subjects with candidemia by blood culture, and should be repeated in subjects who were negative at baseline if the subject develops visual signs or symptoms of a *Candida* eye infection during the study. Additionally, any neutropenic subjects who recover their neutrophil counts during the study should have an additional retinal examination. Subjects who were negative at baseline and diagnosed with a *Candida* eye infection after initiating study drug should have an urgent ophthalmologic consultation (if not already done), should stop study drug, and be initiated on appropriate therapy for *Candida* eye infection per the local guidelines. If possible, all subjects diagnosed with endophthalmitis or chorioretinitis should remain in the study and all subsequent antifungal drugs, interventions, and outcomes for endophthalmitis or chorioretinitis should be recorded.

  Study drug may be continued in the event of diagnosis of other forms of invasive candidiasis listed in the exclusion criteria if the involvement of other organ systems was thought to be present at the time of enrollment and the diagnosis was delayed. If, on the other hand, the spread of *Candida* infection to other organ systems was thought to have occurred following enrollment and initiation of study drug, the Investigator should consider a change of therapy due to insufficient therapeutic effect of study drug. In all cases of other forms of invasive candidiasis, the Investigator should consult local guidelines for the most appropriate management of disease and whether or not additional antifungal therapy may be warranted either during study drug administration or following completion of study drug therapy.

- Insufficient therapeutic effect

  An insufficient therapeutic effect may be determined prior to the planned duration of therapy in the study. This determination will require an assessment of clinical status including the synthesis of symptoms and signs data (both local and systemic). Subjects who are deemed to have an insufficient therapeutic effect should be discontinued from study drug therapy.

  If a subject has daily blood cultures positive for *Candida* spp. through Day 7 despite appropriate study drug administration, this should be considered an insufficient therapeutic effect and study drug should be discontinued and salvage therapy initiated. The subject should remain in the study through the Follow-up visit (Days 52–59) to complete safety assessments and assess for mortality.

  Seven days is the maximum number of daily positive blood cultures allowed prior to a change in antifungal therapy. The Investigator may determine that there is an insufficient therapeutic response prior to Day 7.

- Investigator discretion
  - Resistant Pathogen(s): In the event an organism resistant to ≥1 of the potential study drugs is isolated, the Investigator will determine whether the subject remains on study drug therapy. The Investigator may decide to continue study drug therapy if, in the Investigator's opinion, there is clear and continuing clinical improvement while on therapy.
  - The Investigator may decide to prematurely discontinue study drug therapy and to initiate an alternative and appropriate therapy if, in the Investigator's opinion, the subject is not benefiting from study drug.

NOTE: The Medical Monitor should be contacted to discuss whether to continue or prematurely discontinue a subject on the study drug in the event the subject requires systemic concomitant antifungal therapy.

In the event salvage therapy is required due to either insufficient therapeutic effect or Investigator discretion, the Investigator should note that since the subject has failed echinocandin therapy, other echinocandins may not be the best choice for salvage therapy. If the subject is switched to oral step-down therapy prior to study drug failure, the Investigator should consider that subjects in the caspofungin arm will also have been taking fluconazole. In both circumstances, the Investigator should refer to the IDSA (Pappas 2016) or ESCMID (Cornely 2012; Ullmann 2012) guidelines for treatment failure in candidemia, and will need to determine whether or not breaking the blind is necessary to make an informed choice of future antifungal therapies.

7.5 Replacement of Subjects

Randomized subjects who are withdrawn will not be replaced.

7.6 Study Termination by Sponsor and Termination Criteria

The Sponsor reserves the right to terminate an investigational site or this clinical study at any time. Reasons for termination may include, but are not limited to, the following:

- The incidence or severity of AEs in this or other studies of Rezafungin for Injection indicate a potential health hazard to subjects
- Serious or persistent noncompliance by the Investigator with the protocol, clinical research agreement, or applicable regulatory guidelines in conducting the study
- IRB/IEC decision to terminate or suspend approval for the investigation or the Investigator
- Investigator request to withdraw from participation
- Subject enrollment is unsatisfactory

8.0 STUDY DRUGS

Subjects will be randomized in a 1:1 ratio to receive Rezafungin for Injection IV or Caspofungin IV. Oral step-down therapy is allowed in both treatment groups; oral placebo in the Rezafungin for Injection group and oral fluconazole in the Caspofungin group. The total IV plus oral treatment duration will be ≥14 days and up to a maximum of 28 days (Table 3).

In order to stop study drug for successful completion of therapy prior to 28 days, the following criteria must be met:

- ≥14 days of study drug
- The subject's clinical status is considered stable based on Investigator assessment
- All systemic signs attributable to candidemia and/or invasive candidiasis that were present at baseline have resolved
- For subjects with invasive candidiasis, there is documented or presumed eradication of invasive candidiasis
- If a blood culture is positive at Screening, the most recent post-baseline blood culture is negative for *Candida* spp.

Once these criteria are met, the total duration of study drug is at the discretion of the PI.

Subjects randomized to the Rezafungin for Injection treatment group will receive 1 dose of Rezafungin for Injection IV (400 mg) nominally over 60 (±10) minutes on Day 1 and 200 mg on Day 8 (±1 day) (Table 3). Subjects in the Rezafungin for Injection treatment group who require >14 days of therapy will receive an optional third dose of Rezafungin or Injection IV (200 mg) on Day 15. Subjects in the Rezafungin for Injection treatment group who require >21 days of therapy will receive an optional fourth dose of Rezafungin IV (200 mg) on Day 22. Subjects in the Rezafungin for Injection treatment group will receive IV or oral placebo on other study days in order to maintain the blind. Intravenous study drug is administered 24 (±2) hours after study drug was administered on the previous day.

Subjects may receive oral step-down therapy after ≥3 days of IV therapy or the minimum duration of IV therapy advised by the site's national/regional/local guidelines, whichever is greater. Step-down therapy in the Rezafungin for Injection treatment group will be oral placebo, in order to maintain the blind. Subjects who have already switched to oral step-down therapy will receive both oral placebo and Rezafungin for Injection IV on Day 8 (±1 day), Day 15 (±1 day) for subjects who require >14 days of therapy, and Day 22 (±1 day) for subjects who require >21 days of therapy.

Subjects randomized to the caspofungin group will receive IV caspofungin (a single 70 mg loading dose on Day 1 followed by 50 mg once daily over 60 (±10) minutes) for ≥3 days and up to a maximum of 28 days (Table 3). Intravenous study drug is administered 24 (±2) hours after study drug was administered on the previous day.

Step-down therapy in the caspofungin treatment group will be oral fluconazole (subjects with a creatinine clearance >50 mL/min will receive oral fluconazole at a dose of 6 mg/kg administered once daily rounded to the nearest 200 mg increment with a maximum daily dose of 800 mg; subjects with creatinine clearance ≤50 mL/min will receive oral fluconazole at a dose of 3 mg/kg administered once daily rounded to the nearest 200 mg increment with a maximum daily dose of 400 mg). In order to maintain the blind, subjects who have already switched to oral step-down therapy will receive both oral fluconazole and IV placebo on Day 8 (±1 day), Day 15 (±1 day) for subjects who require >14 days of therapy, and Day 22 (±1 day) for subjects who require >21 days of therapy.

Intravenous infusion of Rezafungin for Injection and IV placebo is administered over 60 (±10) minutes, although the infusion time may be increased up to 180 minutes to manage evolving symptoms of infusion reaction consistent with management of echinocandin class infusion reactions, whereby decreasing the rate of infusion often alleviates symptoms. Dosage adjustments of Rezafungin for Injection are not allowed. Treatment will be initiated on an inpatient basis; a switch to outpatient IV treatment is allowed at the Investigator's discretion.

The total IV plus oral treatment duration will be ≥14 days and up to 28 days.

The daily dosing schedule with options (i.e., not switched versus switched to oral therapy) for the required treatment period is presented in Figure 2 and for the optional treatment period is in Figure 3. The daily dosing schedule for the entire study period (required and optional) with options (i.e., not switched versus switched to oral therapy) is presented in Table 3.

8.1 Rezafungin for Injection

8.1.1 Formulation

Rezafungin for Injection is a lyophilized formulation of the active pharmaceutical ingredient, rezafungin acetate. Rezafungin for Injection is supplied in vials as a sterile lyophilized powder (200 mg) for reconstitution prior to dilution into normal saline infusion bags (250 mL). Excipients for rezafungin include polysorbate 80, histidine, and mannitol.

8.1.2 Directions for Use

Rezafungin for Injection is administered IV with a 400 mg loading dose on Day 1 of Week 1, followed by 200 mg once weekly, for a total of 2 to 4 doses.

Intravenous infusion of Rezafungin for Injection and IV placebo is administered over 60 (±10) minutes, although the infusion time may be increased up to 180 minutes as needed to manage evolving symptoms of infusion reaction consistent with management of echinocandin class infusion reactions, whereby decreasing the rate of infusion often alleviates symptoms. Dosage adjustments of Rezafungin for Injection are not allowed. Treatment will be initiated on an inpatient basis; a switch to outpatient IV treatment is allowed at the Investigator's discretion.

8.1.3 Drug Storage

Vials of Rezafungin for Injection and infusion bags with diluted Rezafungin for Injection should be stored per the directions presented in the study Pharmacy Manual.

8.1.4 Dose Adjustment

Dosage adjustments are not allowed in this study.

8.2 Placebo and Comparator Treatment

8.2.1 Placebo

In this double-blind, double-dummy, active comparator study, two placebo regimens are required; one for subjects randomized to Rezafungin for Injection and one for subjects randomized to caspofungin. In addition, because subjects in the caspofungin arm may be switched to oral step-down therapy, an oral placebo is required for subjects in the Rezafungin for Injection arm. Thus, placebo requirements for each treatment assignment are as follows:

Treatment Assignment	Placebo
Rezafungin for Injection	Placebo for caspofungin IV<br>Placebo for oral step-down therapy (fluconazole)
Caspofungin IV	Placebo for Rezafungin for Injection

In more detail, placebo regimens for each treatment assignment are as follows:

Placebo Regimen for Rezafungin for Injection Treatment Assignment

Subjects will receive a caspofungin IV equivalent regimen of daily placebo IV infusion in a blinded manner. Because subjects randomized to caspofungin may be switched to fluconazole oral step-down therapy, a fluconazole-equivalent regimen of daily oral placebo will be administered for subjects randomized to Rezafungin for Injection at that time.

Placebo Regimen for Caspofungin IV Treatment Assignment

Subjects who are switched to fluconazole oral step-down therapy will receive a Rezafungin for Injection equivalent regimen of weekly placebo IV infusion in a blinded manner.

8.2.2 Caspofungin

Anaphylaxis has been reported with administration of caspofungin. If this occurs, caspofungin should be discontinued immediately and appropriate treatment administered.

Possible histamine-like reactions, including rash, facial swelling, sensation of warmth, pruritis, and bronchospasm, have been observed during infusion of caspofungin. These reactions may require discontinuation of the infusion and/or administration of appropriate treatment.

Cases of Stevens-Johnson Syndrome and toxic epidermal necrolysis have been reported after post-marketing use of caspofungin.

Finally, abnormal liver function tests have been observed with healthy volunteers and patients treated with caspofungin. In patients with serious underlying conditions, cases of clinically significant hepatic dysfunction, hepatitis, and hepatic failure have been reported without a causal relationship established.

Levels of caspofungin may increase with concomitant administration of cyclosporine and may decrease with concomitant use of efavirenz, nevirapine, rifampin, dexamethasone, phenytoin, and carbamazepine.

See Appendix 7A for potential caspofungin drug interactions, warnings, and precautions. The list is not exhaustive, and the Investigator must refer to the US Prescribing Information or Summary of Product Characteristics (SmPC).

8.2.2.1 Formulation

Caspofungin IV is provided in its commercial formulation. Excipients for caspofungin include sucrose, mannitol, glacial acetic acid, and sodium hydroxide (refer to the US Prescribing Information or SmPC for excipients used in specific generic versions).

8.2.2.2 Directions for Use

The manufacturer's instructions for caspofungin dose preparation should be followed.

Subjects randomized to caspofungin will receive a total treatment of ≥14 days beginning with a single caspofungin 70 mg IV loading dose on Day 1 followed by 50 mg once daily with the option to continue treatment ≤28 days, unless they qualify for an alternative caspofungin regimen per Section 8.2.2.4 or for step-down therapy after ≥3 days (or the minimum duration of IV therapy advised by the site's national/regional/local guidelines, whichever is greater) of caspofungin IV treatment per Section 8.2.3. Doses are to be administered 24 (±2) hours after the start of study drug administration on the previous day. The infusion time for caspofungin is the same as for Rezafungin for Injection. The infusion time of time of 60 (±10) minutes may be increased as needed up to 180 minutes to manage evolving symptoms of infusion reaction consistent with management of echinocandin class infusion reactions, whereby decreasing the rate of infusion often alleviates symptoms.

8.2.2.3 Drug Storage

The manufacturer's instructions for caspofungin storage should be followed.

8.2.2.4 Dose Adjustment

Subjects with moderate hepatic impairment (Child-Pugh score of 7–9): loading dose of caspofungin of 70 mg on Day 1 followed by 35 mg once daily.

Subjects weighing >80 kg or on concomitant rifampin, nevirapine, efavirenz, phenytoin, dexamethasone, or carbamazepine: caspofungin 70 mg once daily. Dose adjustment due to DDIs or weight may be considered according to the approved labeling and at the Investigator's discretion.

Use of concomitant cyclosporine should be limited to subjects for whom potential benefit outweighs potential risk of DDIs and hepatotoxicity with caspofungin.

8.2.3 Oral Step-Down Therapy: Fluconazole

Fluconazole should not be co-administered with terfenadine, cisapride, astemizole, erythromycin, pimozide, quinidine, halofantrine, or any other drug metabolized via the CYP3A4 enzymatic pathway and may cause prolongation of the QT interval.

Fluconazole is a potent CYP2C9 inhibitor and a moderate CYP3A4 inhibitor and thus all subjects on fluconazole who are concomitantly taking drugs that are also metabolized through these pathways should be monitored closely.

See Appendix 7B for potential fluconazole drug interactions, warnings, and precautions. The list is not exhaustive, and the investigator must refer to the US Prescribing Information or SmPC.

8.2.3.1 Formulation

Commercial fluconazole tablets have been over-encapsulated for study blinding. Excipients for fluconazole include microcrystalline cellulose, dibasic calcium phosphate anhydrous, povidone, croscarmellose sodium, FD&C Red Dye No. 40 aluminum lake dye, and magnesium stearate.

8.2.3.2 Directions for Use

After ≥3 days of caspofungin treatment (or the minimum duration of IV therapy advised by the site's national/regional/local guidelines, whichever is greater), subjects who meet the step-down therapy eligibility criteria may be switched to oral fluconazole (earliest oral dose on Day 4 or later, as advised by a site's national/regional/local guidelines). The total comparator treatment period (caspofungin plus fluconazole) is ≤28 days. Subjects with a creatinine clearance >50 mL/min (see Appendix 2) will receive oral fluconazole at a dose of 6 mg/kg administered once daily rounded to the nearest 200 mg increment with a maximum daily dose of 800 mg (e.g., a subject weighing 73 kg would receive a fluconazole 400 mg dose [2 capsules of 200 mg each] based on a 6 mg/kg target dose [73 kg × 6 mg/kg = 438 mg). Subjects with creatinine clearance ≤50 mL/min will receive oral fluconazole at a dose of 3 mg/kg administered once daily rounded to the nearest 200 mg increment with a maximum daily dose of 400 mg (e.g., a subject

weighing 73 kg would receive a fluconazole 200 mg dose [1 capsule of 200 mg] based on a 3 mg/kg target dose [73 kg × 3 mg/kg = 219 mg]). Subjects receiving hemodialysis receive the same dose as subjects with a creatinine clearance >50 mL/min; however, they are only dosed following hemodialysis. Subjects receiving continuous renal replacement therapy (CRRT) should be treated as a subject with normal renal function at 6 mg/kg rounded to the nearest 200 mg each day (Cousin 2003). Subjects receiving peritoneal dialysis should be treated with 1.5 mg/kg rounded to the nearest 200 mg each day (Cousin 2003).

Investigators are advised to pay close attention to signs of overdosing and underdosing with underweight (<50 kg) and overweight (>85 kg) subjects, respectively. Additionally, signs of potential fluconazole toxicity, including hepatic toxicity and QT prolongation, should be closely monitored for any subject on oral step-down therapy. Subjects weighing over 130 kg will not be eligible for oral step-down therapy.

8.2.3.3 Oral Step-Down Therapy Eligibility Criteria

An oral step-down therapy is allowed in both treatment groups (fluconazole for caspofungin, placebo for oral step-down therapy for Rezafungin for Injection), provided the following criteria are met:

- Able to take oral medication
- ≥3 days of IV study drug (or the minimum duration of IV therapy advised by the site's national/regional/local guidelines, whichever is greater)
- The *Candida* spp. isolated is susceptible to fluconazole
- All signs and symptoms of candidemia and/or invasive candidiasis that were present at baseline have resolved
- The subject's clinical status is considered stable based on Investigator assessment
- Most recent blood culture must be drawn following the first dose of study drug AND ≥48 hours prior to oral study drug initiation AND must be negative for *Candida* spp.
- No evidence of moderate or severe hepatic injury (alanine aminotransferase or aspartate aminotransferase >5× the ULN)
- No history of hypersensitivity to any azole or any other contraindications to the use of fluconazole or its excipients (Appendix 7B), including concomitant use of the following medications: terfenadine, cisapride, astemizole, erythromycin, pimozide, and quinidine
- No personal or family history of Long QT interval on ECG (QT) syndrome or a prolonged QT interval corrected for heart rate by Fridericia's formula (QTcF) interval (>470 msec in males and >480 msec in females)
- In the Investigator's opinion, the subject can tolerate oral fluconazole therapy (refer to current fluconazole Prescribing Information)

- Subject's weight is ≤130 kg

While QT prolongation is rare with fluconazole without the contraindicated interacting medications listed above, investigators should monitor subjects with ECGs as clinically indicated.

Subjects who are switched to oral step-down therapy may switch back to IV study drug therapy in the event of the development of an unforeseen condition that prevents the subject from taking oral medications (e.g., pancreatitis, urgent surgery), but may not switch back to IV study drug therapy for worsening or relapse of candidemia/invasive candidiasis or for intolerance or toxicity due to study drug.

The daily dosing schedule with options for the required treatment period is presented in Figure 2 and for the optional treatment period is in Figure 3. The daily dosing schedule for the entire study period (required and optional) is presented in Table 3.

8.2.3.4 Drug Storage

Capsule containing fluconazole tablets (over-encapsulated fluconazole) should be stored per the directions presented in the study Pharmacy Manual.

8.2.3.5 Dose Adjustment for Renal Impairment

Subjects with creatinine clearance ≤50 mL/min (see Appendix 2) will receive oral fluconazole at a dose of 3 mg/kg administered once daily rounded to the nearest 200 mg increment with a maximum daily dose of 400 mg. Subjects receiving hemodialysis receive the same dose as subjects with a creatinine clearance >50 mL/min; however, they are only dosed following hemodialysis. Subjects receiving CRRT should be treated as a subject with normal renal function at 6 mg/kg rounded to the nearest 200 mg each day (Cousin 2003). Subjects receiving peritoneal dialysis should be treated with 1.5 mg/kg rounded to the nearest 200 mg each day (Cousin 2003).

8.3 Compliance

Treatment compliance for IV study drugs will be documented in the eCRF by recording the date, start time, stop time, and whether the dose of study drug was completely infused. For oral fluconazole (caspofungin group) or placebo for oral step-down therapy (Rezafungin for Injection groups), treatment compliance will be documented by recording start date, end date, and pill counts.

8.4 Breaking the Blind

The study is a double-blind design. The Sponsor, PI, study site personnel, and subjects will not make any effort to determine which blinded study drug therapy (Rezafungin for Injection or caspofungin with the option of step-down therapy) is being administered or received.

Unblinded pharmacy personnel will be responsible for preparing infusion bags such that the double-blind is maintained.

Only in the case of an emergency, when knowledge of the study drug is essential for the clinical management or welfare of a specific subject, may the PI unblind a subject's treatment assignment.

As soon as possible and without revealing the subject's study treatment assignment (unless important to the safety of subjects remaining in the study), the PI must notify the Sponsor if the blind is broken for any reason. The PI will record in source documentation the date and reason for revealing the blinded treatment assignment for that subject.

Unblinded data will be provided to the DSMB for the interim analysis of safety as detailed in the DSMB charter. No interim unblinded data will be provided to the Sponsor. After the database is locked and the Statistical Analysis Plan (SAP) is final, the study blind codes will be broken.

8.5 Prior and Concomitant Medications and Substances

All systemic antifungal therapy administered within 4 weeks and all non-antifungal therapy administered within 1 week prior to randomization will be documented and recorded in the eCRF. Subjects who received systemic treatment with an antifungal agent at approved doses for treatment of candidemia for >48 hours (e.g., >2 doses of a once daily antifungal agent or >4 doses of a twice daily antifungal agent) ≤4 days (96 hours) before randomization will be excluded from the study.

Concomitant systemic antifungal agents, other than those listed as part of study drug therapy, are not permitted and their use, for any reason other than the subject being considered a treatment failure, must be discussed with the Medical Monitor before administration.

Caspofungin is contraindicated in subjects with hypersensitivity to the active substance or any of the excipients (listed in Section 8.2.2.1). Hypersensitivity includes but is not limited to hereditary sugar disorders (e.g., fructose intolerance or sucrose-isomaltase deficiency), anaphylaxis and exfoliative skin disorders (e.g., Stevens-Johnson syndrome or toxic epidermal necrolysis), which may be severe.

Caspofungin is not a substrate for P-glycoprotein and is a poor substrate for cytochrome P450 enzymes. However, caspofungin has been shown to interact with other medicinal products in pharmacological and clinical studies.

For subjects receiving caspofungin and tacrolimus, standard monitoring of tacrolimus trough whole blood concentrations and appropriate tacrolimus dosage adjustments are recommended.

Rifampin is a potent CYP3A4 inducer and concomitant administration with caspofungin is expected to reduce the plasma concentrations of caspofungin. A similar effect could be expected for other medicinal products that induce metabolic enzymes, therefore subjects on rifampin

or the inducers efavirenz, nevirapine, dexamethasone, phenytoin, or carbamazepine may receive caspofungin 70 mg once daily.

Use of concomitant cyclosporine should be limited to subjects for whom potential benefit outweighs potential risk of DDIs and hepatotoxicity with caspofungin. All other concomitant medications necessary for the health and wellbeing of the subject are permitted.

Fluconazole is contraindicated in subjects with hypersensitivity to fluconazole and should be used with caution in subjects with hypersensitivity to other azoles. Hypersensitivity includes but is not limited to anaphylaxis and exfoliative skin disorders, which may be severe. Fluconazole should not be co-administered with terfenadine, cisapride, astemizole, erythromycin, pimozide, quinidine, or any other drug metabolized via the CYP3A4 enzymatic pathway and may cause prolongation of the QT interval. Fluconazole should be administered with caution in patients with evidence of hepatic toxicity or liver dysfunction. Patients who have or develop abnormal liver function tests during fluconazole therapy should be monitored for the development of more severe hepatic injury. Hepatic toxicity secondary to fluconazole is usually reversible. Finally, subjects should use caution when driving or operating machinery as dizziness and seizures can occur with fluconazole.

Fluconazole is a potent CYP2C9 inhibitor and a moderate CYP3A4 and CYP2C9 inhibitor and thus all subjects on fluconazole who are concomitantly taking drugs that are also metabolized through these pathways should be monitored closely. Please refer to the US Prescribing Information or the European Union Summary of Product Characteristics for fluconazole for more information about the contraindications, warnings and precautions, and likely DDIs.

8.6 Accountability Procedures

The pharmacy or other unblinded study personnel are responsible for ensuring that a current record of Rezafungin for Injection, caspofungin, fluconazole, and placebo inventory and accountability is maintained.

A Pharmacy Monitor will be responsible for checking study drug accountability at the site. Inventory records must be readily available for inspection by regulatory authorities at any time. Each shipment of study drug will contain an acknowledgment of receipt section for site signature. Upon receipt of study drug, the pharmacy or study personnel will visually inspect the shipment and verify the number and condition of vials or capsules received. Refer to the study Pharmacy Manual for additional information.

8.7 Study Drug Handling and Disposal

Unless expressly disallowed by institution rules, used and unused vials of study drug (including Rezafungin for Injection and caspofungin) will be retained at the study site until study drug accountability has been performed by the Pharmacy Monitor. Upon completion of the study,

termination of the study, or upon written authorization from the Sponsor, all retained unused and partially used study drug will be centrally destroyed. All records of disposal by a centralized destruction site will be maintained by the Sponsor.

9.0 STUDY PROCEDURES

Screening procedures are summarized in Section 9.1, the Required Treatment Period procedures and Follow-up procedures are summarized in Section 9.2 and Section 9.3, respectively, and the Optional Treatment Period (Days 15–28) procedures are summarized in Section 9.4.

Study procedures should be completed within the windows provided in the Schedule of Assessments and Procedures for the Required Treatment, EOT, and Follow-up Periods in Table 1 and the Schedule of Assessments and Procedures for the Optional Treatment Period in Table 2, when applicable. However, if a subject is unable to attend a visit within the specified windows, the PI (or qualified designee) should discuss appropriate scheduling with the Medical Monitor (or appropriate designee).

9.1 Screening (≤4 days [96 hours])

- Obtain written informed consent prior to initiating any study related assessments or procedures. Consent from a legally acceptable representative (i.e. acceptable to ICH and local law, as applicable) may be obtained if the subject is unable to consent for themselves.
- Obtain a complete medical history for the last 5 years and *Candida* risk factors for the last 3 months (e.g., central line, active malignancy, broad-spectrum antibiotic therapy, diabetes mellitus, immunosuppression, major surgery, total parenteral nutrition, transplant recipient, trauma, dialysis, burns, pancreatitis) and ICU admission and discharge (if applicable).
- Record all prior and/or concomitant medications.
- Presence of central line during Screening or placed during the study should be documented with dates and times of insertion and removal.
- Short-term central venous catheters (e.g., peripherally-inserted central catheters, internal jugular, or subclavian central venous catheters) and long-term central venous catheters (e.g., tunneled catheters) are recommended to be removed within 48 hours after diagnosis with candidemia, consistent with IDSA (Pappas 2016) and ESCMID (Cornely 2012; Ullmann 2012) guidelines.
- Record all systemic antifungal therapy administered within 4 weeks and all therapies (non-antifungal) within 1 week prior to randomization.
- Clinical Assessments:
    - Conduct complete physical examination, including weight and height.
    - Conduct a thorough neurological examination as part of the physical examination to confirm no evidence of Grade 2 ataxia, tremor, sensory or motor neuropathy based on CTCAE version 5.0 criteria.

- Measure vital signs (temperature and the method used [oral, rectal, temporal, tympanic, or core], heart rate, blood pressure, and respiratory rate). Axillary temperatures are allowed, but not preferred.
- Perform 12-lead ECG prior to randomization.
- Assess presence or absence of systemic signs and symptoms and determine which are attributable to candidemia and/or invasive candidiasis. The Screening period for assessing systemic signs for inclusion in the study may include the 12 hours prior to the collection of the qualifying positive blood culture when systemic signs of infection were the reason for specimen collection (blood culture or fluid from a sterile site culture or qualifying rapid IVD [e.g., T2Candida Panel]) through study randomization.
- Retinal examination for *Candida* eye infection (may occur as late as Day 7). Retinal examination is standard of care for subjects with candidemia and should be performed by an ophthalmologist, when available.

- Laboratory assessments:
  - Draw blood for hematology and chemistry.
  - Draw blood for coagulation panel (PT/INR).
  - **Only for subjects enrolled prior to Amendment 5:** Draw PK blood sample for protein binding and for albumin core analyses. To limit invasive procedures these samples would ideally be collected at the same time into two vacutainers, though each sample could be collected separately.
  - Collect urine for urinalyses, microscopy. Subjects who are anuric do not need to complete this assessment. Urine microscopy is only required in the presence of abnormal urine chemistry tests.
  - Conduct serum pregnancy test (women of childbearing potential only; do not perform for women ≥2 years postmenopausal or surgically sterile).
  - A culture must be obtained as part of the standard of care for inclusion in the study. Established mycological diagnosis of candidemia or invasive candidiasis from a sample taken ≤4 days (96 hours) before randomization defined as one of the following:
    - ≥1 blood culture positive for yeast or *Candida.*
    - a positive test for *Candida* from sponsor-approved rapid IVD.
    - a positive gram stain (or other method of direct microscopy) for yeast or positive culture for *Candida* spp. from a specimen obtained from a normally sterile site.

NOTE: If the positive blood culture used to qualify the subject for the study is drawn >12 hours prior to randomization, an additional set of blood cultures must be obtained ≤12 hours before randomization. Identification and susceptibility testing at the local laboratory for *Candida* for blood or normally sterile tissue/fluid for culture is required at Screening and for any positive culture requiring a change of antifungal therapy (i.e., local identification and susceptibility testing is not required for *Candida* isolates cultured from specimens on other study days provided there is no required change of antifungal therapy).

  - If the subject is suspected of having invasive candidiasis, then all reasonable attempts to obtain a culture from the suspected site of infection should be made as early as possible before empiric therapy.
  - Perform identification and susceptibility testing at local laboratory for *Candida* for any positive blood culture or any positive culture from normally sterile site.
  - Record the spp. and susceptibilities for all bacteria isolated within 1 week prior to randomization from blood or any other normally sterile site.
  - Send all fungal isolates cultured from blood or normally sterile tissue/fluid to the Central Laboratory.

- Calculate Child-Pugh score only if the subject has a history of chronic cirrhosis (see Appendix 4).
- Record modified APACHE II parameters (see Appendix 5) with Glasgow Coma Score (see Appendix 6); note that the APACHE II score must be calculated prior to enrollment using the vital signs and laboratory results from the Screening visit. If multiple vital signs and laboratory values are obtained during the Screening period, it is preferred to use those collected most closely to the time of randomization.
- Determine ANC for stratification from the most recent hematology results obtained prior to randomization.
- If radiologic test is performed, record radiologic test type with Findings and Interpretation only if radiologic test provides evidence of invasive candidiasis.
- Assess, identify, and record any AEs following signing of the ICF.
- Confirm subject qualification by inclusion and exclusion criteria.

9.2 Required Treatment Period

9.2.1 Day 1

- Randomize subject just prior to dosing.
- Administer IV infusion of study drug over 60 (±10) minutes (refer to Section 8.0 and Study Pharmacy Manual for additional infusion rate guidance).
- Clinical Assessments:

- A focused physical exam (including a neurological exam) should be performed as clinically indicated by AE screening and course of the underlying disease.
- Measure vital signs (temperature and the method used [oral, rectal, temporal, tympanic, or core], heart rate, blood pressure, and respiratory rate). Axillary temperatures are allowed, but not preferred.
- If not completed during Screening, perform retinal examination for *Candida* eye infection for subjects with candidemia by blood culture (by Day 7). Additionally, any neutropenic subjects who recover their neutrophil counts during the study should have an additional retinal examination. The retinal examination should be repeated in subjects who were negative at baseline if the subject develops visual signs or symptoms of a *Candida* eye infection during the study. Subjects who were negative at baseline and diagnosed with a *Candida* eye infection after initiating study drug must discontinue study drug (see Section 7.4.2) but may continue in the study at the Investigator's discretion; all subsequent antifungal drugs, interventions, and outcomes for endophthalmitis or chorioretinitis will be recorded.
- Perform 12-lead ECG following administration of study drug (i.e., post-end IV infusion).

- Laboratory Assessments:
  - Draw blood for hematology and serum chemistry tests (see Appendix 3); for subjects having routine daily blood draws for hematology and serum chemistry as standard of care, values to be entered into the eCRF will generally be the first draw of the day.
  - Draw blood or normally sterile tissue/fluid (if possible) for culture.
    - Perform identification and susceptibility testing for *Candida* for any positive culture requiring a change of antifungal therapy (i.e., identification and susceptibility testing not required for *Candida* isolates cultured from specimens obtained without a required change in antifungal therapy).
    - All fungal isolates cultured from blood and normally sterile tissue/fluid must be sent to the Central Laboratory.
    - Record the spp. for any new bacteria isolated from blood and the spp. and susceptibilities for any new bacteria isolated from any other normally sterile site.
- One blood sample for PK will be collected within 10 minutes before the end of infusion, and one sample taken any time between the end of infusion and 12 hours after the end of infusion. Draw blood for PK analysis from the OPPOSITE arm or through an arterial line during the infusion; after the infusion is complete opposite arm or arterial line draws are preferred. For subjects receiving hemodialysis, an attempt should be made to obtain PK samples directly following hemodialysis if still within the required time window.
- Record all prior and/or concomitant medications.
- Record AEs.

- Presence of central line during Screening or placed during the study should be documented with dates and times of insertion and removal.
- Short-term central venous catheters (e.g., peripherally-inserted central catheters, internal jugular, or subclavian central venous catheters) and long-term central venous catheters (e.g., tunneled catheters) are recommended to be removed within 48 hours after diagnosis with candidemia, consistent with IDSA (Pappas 2016) and ESCMID (Cornely 2012; Ullmann 2012) guidelines.
- If radiologic test is performed, record radiologic test type with Findings and Interpretation only if radiologic test provides evidence of invasive candidiasis or evidence of progression, stabilization, improvement, or resolution of invasive candidiasis from previous radiographs. Criteria for cure, indeterminate, and failure responses are found in Table 10.

9.2.2 Day 2

- Administer IV infusion of study drug over 60 (±10) minutes (refer to Section 8.0 and Study Pharmacy Manual for additional infusion rate guidance).
- Clinical Assessments:
  - A focused physical exam (including a neurological exam) should be performed as clinically indicated by AE screening and course of the underlying disease.
  - Measure vital signs (temperature and the method used [oral, rectal, temporal, tympanic, or core], heart rate, blood pressure, and respiratory rate). Axillary temperatures are allowed, but not preferred.
  - If not completed during Screening, perform retinal examination for *Candida* eye infection for subjects with candidemia by blood culture (by Day 7). Additionally, any neutropenic subjects who recover their neutrophil counts during the study should have an additional retinal examination. The retinal examination should be repeated in subjects who were negative at baseline if the subject develops visual signs or symptoms of a *Candida* eye infection during the study. Subjects who were negative at baseline and are diagnosed with a *Candida* eye infection after initiating study drug must discontinue study drug (see Section 7.4.2) but may continue in the study at the Investigator's discretion; all subsequent antifungal drugs, interventions, and outcomes for endophthalmitis or chorioretinitis will be recorded.
- Laboratory Assessments:
  - Draw blood for hematology and serum chemistry tests (see Appendix 1); for subjects having routine daily blood draws for hematology and serum chemistry as standard of care, values to be entered into the eCRF will generally be the first draw of the day.
  - Draw blood or normally sterile tissue/fluid (if possible) for culture.

    - Perform identification and susceptibility testing for *Candida* for any positive culture requiring a change of antifungal therapy (i.e., identification and susceptibility testing not required for *Candida* isolates cultured from specimens obtained without a required change in antifungal therapy).
    - All fungal isolates cultured from blood and normally sterile tissue/fluid must be sent to the Central Laboratory.
    - Record the spp. for any new bacteria isolated from blood and the spp. and susceptibilities for any new bacteria isolated from any other normally sterile site.
  - One blood sample for PK will be collected at any time on one of the following Days 2, 3, 4, or 5 (i.e., if blood sample for PK not collected on Day 2, must be collected on Day 3 [or Day 4 or Day 5]). Draw blood for PK analysis from the OPPOSITE arm or through an arterial line during the infusion; after the infusion is complete opposite arm or arterial line draws are preferred. For subjects receiving hemodialysis, an attempt should be made to obtain PK samples directly following hemodialysis if still within the required time window.
- Record all concomitant medications.
- Record AEs.
- Presence of central line during Screening or placed during the study should be documented with dates and times of insertion and removal.
- Short-term central venous catheters (e.g., peripherally-inserted central catheters, internal jugular, or subclavian central venous catheters) and long-term central venous catheters (e.g., tunneled catheters) are recommended to be removed within 48 hours after diagnosis with candidemia, consistent with IDSA (Pappas 2016) and ESCMID (Cornely 2012; Ullmann 2012) guidelines.
- If radiologic test is performed, record radiologic test type with Findings and Interpretation only if radiologic test provides evidence of invasive candidiasis or evidence of progression, stabilization, improvement, or resolution of invasive candidiasis from previous radiographs. Criteria for cure, indeterminate, and failure responses are found in Table 10.

9.2.3 Day 3

- Administer IV infusion of study drug over 60 (±10) minutes (refer to Section 8.0 and Study Pharmacy Manual for additional infusion rate guidance).
- Clinical Assessments:
  - A focused physical exam (including a neurological exam) should be performed as clinically indicated by AE screening and course of the underlying disease.

- Measure vital signs (temperature and the method used [oral, rectal, temporal, tympanic, or core], heart rate, blood pressure, and respiratory rate). Axillary temperatures are allowed, but not preferred.
- If not completed during Screening, perform retinal examination for *Candida* eye infection for subjects with candidemia by blood culture (by Day 7). Additionally, any neutropenic subjects who recover their neutrophil counts during the study should have an additional retinal examination. The retinal examination should be repeated in subjects who were negative at baseline if the subject develops visual signs or symptoms of a *Candida* eye infection during the study. Subjects who were negative at baseline and are diagnosed with a *Candida* eye infection after initiating study drug must discontinue study drug (see Section 7.4.2) but may continue in the study at the Investigator's discretion; all subsequent antifungal drugs, interventions, and outcomes for endophthalmitis or chorioretinitis will be recorded.

- Laboratory Assessments:
  - Draw blood or normally sterile tissue/fluid (if possible) for culture if demonstrating mycological eradication (if not already done) or if clinically indicated.
    - Perform identification and susceptibility testing for *Candida* for any positive culture requiring a change of antifungal therapy (i.e., identification and susceptibility testing not required for *Candida* isolates cultured from specimens obtained without a required change in antifungal therapy).
    - All fungal isolates cultured from blood and normally sterile tissue/fluid must be sent to the Central Laboratory.
    - Record the spp. for any new bacteria isolated from blood and the spp. and susceptibilities for any new bacteria isolated from any other normally sterile site.
  - One blood sample for PK will be collected at any time on Days 2, 3, 4, or 5 (i.e., if blood sample for PK not collected on Day 2, must be collected on Day 3 [or Day 4 or Day 5]). Draw blood for PK analysis from the OPPOSITE arm or through an arterial line during the infusion; after the infusion is complete opposite arm or arterial line draws are preferred. For subjects receiving hemodialysis, an attempt should be made to obtain PK samples directly following hemodialysis if still within the required time window.
- Record all concomitant medications.
- Record AEs.
- Presence of central line during Screening or placed during the study should be documented with dates and times of insertion and removal.

- Short-term central venous catheters (e.g., peripherally-inserted central catheters, internal jugular, or subclavian central venous catheters) and long-term central venous catheters (e.g., tunneled catheters) are recommended to be removed within 48 hours after diagnosis with candidemia, consistent with IDSA (Pappas 2016) and ESCMID (Cornely 2012; Ullmann 2012) guidelines.
- If radiologic test is performed, record radiologic test type with Findings and Interpretation only if radiologic test provides evidence of invasive candidiasis or evidence of progression, stabilization, improvement, or resolution of invasive candidiasis from previous radiographs. Criteria for cure, indeterminate, and failure responses are found in Table 10.

9.2.4 Day 4

- Administer study drug:
  - IV infusion of study drug over 60 (±10) minutes (refer to Section 8.0 and the Study Pharmacy Manual for additional infusion rate guidance). Administration should occur 24 (±2) hours after study drug was administered on the previous day.

    OR
  - Oral step-down therapy.
- Clinical Assessments:
  - A focused physical exam (including a neurological exam) should be performed as clinically indicated by AE screening and course of the underlying disease.
  - Measure vital signs (temperature and the method used [oral, rectal, temporal, tympanic, or core], heart rate, blood pressure, and respiratory rate). Axillary temperatures are allowed, but not preferred.
  - If not completed during Screening, perform retinal examination for *Candida* eye infection for subjects with candidemia by blood culture (by Day 7). Additionally, any neutropenic subjects who recover their neutrophil counts during the study should have an additional retinal examination. The retinal examination should be repeated in subjects who were negative at baseline if the subject develops visual signs or symptoms of a *Candida* eye infection during the study. Subjects who were negative at baseline and are diagnosed with a *Candida* eye infection after initiating study drug must discontinue study drug (see Section 7.4.2) but may continue in the study at the Investigator's discretion; all subsequent antifungal drugs, interventions, and outcomes for endophthalmitis or chorioretinitis will be recorded.
- Laboratory Assessments:
  - Draw blood for hematology and serum chemistry tests (see Appendix 3); for subjects having routine daily blood draws for hematology and serum chemistry as standard of care, values to be entered into the eCRF with generally be the first draw of the day.

- Draw blood or normally sterile tissue/fluid (if possible) for culture if demonstrating mycological eradication (if not already done) or if clinically indicated.
  - Perform identification and susceptibility testing for *Candida* for any positive culture requiring a change of antifungal therapy (i.e., identification and susceptibility testing not required for *Candida* isolates cultured from specimens obtained without a required change in antifungal therapy).
  - All fungal isolates cultured from blood and normally sterile tissue/fluid must be sent to the Central Laboratory.
  - Record the spp. for any new bacteria isolated from blood and the spp. and susceptibilities for any new bacteria isolated from any other normally sterile site.
- One blood sample for PK will be collected at any time on Days 2, 3, 4, or 5 (i.e., if blood sample for PK not collected on Day 2 or Day 3, must be collected on Day 4 [or Day 5]). Draw blood for PK analysis from the OPPOSITE arm or through an arterial line during the infusion; after the infusion is complete opposite arm or arterial line draws are preferred. For subjects receiving hemodialysis, an attempt should be made to obtain PK samples directly following hemodialysis if still within the required time window.

- Record all concomitant medications.
- Record AEs.
- Presence of central line during Screening or placed during the study should be documented with dates and times of insertion and removal.
- Short-term central venous catheters (e.g., peripherally-inserted central catheters, internal jugular, or subclavian central venous catheters) and long-term central venous catheters (e.g., tunneled catheters) are recommended to be removed within 48 hours after diagnosis with candidemia, consistent with IDSA (Pappas 2016) and ESCMID (Cornely 2012; Ullmann 2012) guidelines.
- If radiologic test is performed, record radiologic test type with Findings and Interpretation only if radiologic test provides evidence of invasive candidiasis or evidence of progression, stabilization, improvement, or resolution of invasive candidiasis from previous radiographs. Criteria for cure, indeterminate, and failure responses are found in Table 10.

9.2.5 Days 5–7

- Administer study drug
  - IV infusion of study drug over 60 (±10) minutes (refer to Section 8.0 and the Study Pharmacy Manual for additional infusion rate guidance). Administration should occur 24 (±2) hours after study drug was administered on the previous day.
    OR
  - Oral step-down therapy.
- Clinical Assessments:
  - Conduct complete physical examination on Day 5 only.
  - Conduct neurological examination as part of the physical examination, as clinically indicated.
  - On Day 6 and Day 7, conduct a focused physical examination (including neurological exam) only if clinically indicated by adverse event screening and course of the underlying disease.
  - Measure vital signs (temperature and the method used [oral, rectal, temporal, tympanic, or core], heart rate, blood pressure, and respiratory rate). Axillary temperatures are allowed, but not preferred.
  - If not completed during Screening, perform retinal examination for *Candida* eye infection for subjects with candidemia by blood culture (by Day 7). Additionally, any neutropenic subjects who recover their neutrophil counts during the study should have an additional retinal examination. The retinal examination should be repeated in subjects who were negative at baseline if the subject develops visual signs or symptoms of a *Candida* eye infection during the study. Subjects who were negative at baseline and are diagnosed with a *Candida* eye infection after initiating study drug must discontinue study drug (see Section 7.4.2) but may continue in the study at the Investigator’s discretion; all subsequent antifungal drugs, interventions, and outcomes for endophthalmitis or chorioretinitis will be recorded.
  - Assess presence or absence of systemic signs and symptoms and determine which are attributable to candidemia and/or invasive candidiasis on Day 5 only.
  - Assess for clinical response on Day 5 only (Table 9).
- Laboratory Assessments:
  - Draw blood or normally sterile tissue/fluid (if possible) for culture if demonstrating mycological eradication (if not already done) or if clinically indicated.

  - Perform identification and susceptibility testing for *Candida* for any positive culture requiring a change of antifungal therapy (i.e., identification and susceptibility testing not required for *Candida* isolates cultured from specimens obtained without a required change in antifungal therapy).
  - All fungal isolates cultured from blood and normally sterile tissue/fluid must be sent to the Central Laboratory.
  - Record the spp. for any new bacteria isolated from blood and the spp. and susceptibilities for any new bacteria isolated from any other normally sterile site.
- On Day 5, one blood sample for PK will be collected at any time if not collected on Day 2 or Day 3 or Day 4. Draw blood for PK analysis from the OPPOSITE arm or through an arterial line during the infusion; after the infusion is complete opposite arm or arterial line draws are preferred. For subjects receiving hemodialysis, an attempt should be made to obtain PK samples directly following hemodialysis if still within the required time window.

- Record all concomitant medications.
- Record AEs.
- Presence of central line during Screening or placed during the study should be documented with dates and times of insertion and removal.
- Short-term central venous catheters (e.g., peripherally-inserted central catheters, internal jugular, or subclavian central venous catheters) and long-term central venous catheters (e.g., tunneled catheters) are recommended to be removed within 48 hours after diagnosis with candidemia, consistent with IDSA (Pappas 2016) and ESCMID (Cornely 2012; Ullmann 2012) guidelines.
- If radiologic test is performed, record radiologic test type with Findings and Interpretation only if radiologic test provides evidence of invasive candidiasis or evidence of progression, stabilization, improvement, or resolution of invasive candidiasis from previous radiographs. Criteria for cure, indeterminate, and failure responses are found in Table 10.

9.2.6 Day 8 (±1 day)

- One blood sample for PK will be collected predose (≤30 minutes prior to the start of the infusion). Draw blood for PK analysis from the OPPOSITE arm or through an arterial line during the infusion; after the infusion is complete, opposite arm or arterial line draws are preferred. For subjects receiving hemodialysis, an attempt should be made to obtain PK samples directly following hemodialysis if still within the required time window.
- Administer study drug:

- IV infusion of study drug over 60 (±10) minutes (refer to Section 8.0 and the Study Pharmacy Manual for additional infusion rate guidance). Administration should occur 24 (±2) hours after study drug was administered on the previous day.

  OR

- IV infusion of study drug over 60 (±10) minutes (refer to Section 8.0 and the Study Pharmacy Manual for additional infusion rate guidance). Administration should occur 24 (±2) hours after study drug was administered on the previous day PLUS oral step-down therapy, if already stepped down.

- Clinical Assessments:
  - A focused physical exam (including a neurological exam) should be performed as clinically indicated by AE screening and course of the underlying disease.
  - Measure vital signs (temperature and the method used [oral, rectal, temporal, tympanic, or core], heart rate, blood pressure, and respiratory rate). Axillary temperatures are allowed, but not preferred.
  - The retinal examination should be repeated in subjects who were negative at baseline if the subject develops visual signs or symptoms of a *Candida* eye infection during the study. Additionally, any neutropenic subjects who recover their neutrophil counts during the study should have an additional retinal examination. Subjects who were negative at baseline and are diagnosed with a *Candida* eye infection after initiating study drug must discontinue study drug (see Section 7.4.2) but may continue in the study at the Investigator's discretion; all subsequent antifungal drugs, interventions, and outcomes for endophthalmitis or chorioretinitis will be recorded.
- Laboratory Assessments:
  - Draw blood for hematology and serum chemistry tests (see Appendix 3); for subjects having routine daily blood draws for hematology and serum chemistry as standard of care, values to be entered into the eCRF with generally be the first draw of the day.
  - Draw blood or normally sterile tissue/fluid (if possible) for culture if demonstrating mycological eradication (if not already done) or if clinically indicated.
    - Perform identification and susceptibility testing for *Candida* for any positive culture requiring a change of antifungal therapy (i.e., identification and susceptibility testing not required for *Candida* isolates cultured from specimens obtained without a required change in antifungal therapy).
    - All fungal isolates cultured from blood and normally sterile tissue/fluid must be sent to the Central Laboratory.
    - Record the spp. for any new bacteria isolated from blood and the spp. and susceptibilities for any new bacteria isolated from any other normally sterile site.

- Record all concomitant medications.
- Record AEs.
- Presence of central line during Screening or placed during the study should be documented with dates and times of insertion and removal.
- Short-term central venous catheters (e.g., peripherally-inserted central catheters, internal jugular, or subclavian central venous catheters) and long-term central venous catheters (e.g., tunneled catheters) are recommended to be removed within 48 hours after diagnosis with candidemia, consistent with IDSA (Pappas 2016) and ESCMID (Cornely 2012; Ullmann 2012) guidelines.
- If radiologic test is performed, record radiologic test type with Findings and Interpretation only if radiologic test provides evidence of invasive candidiasis or evidence of progression, stabilization, improvement, or resolution of invasive candidiasis from previous radiographs. Criteria for cure, indeterminate, and failure responses are found in Table 10.

9.2.7 Days 9–13

- Administer study drug:
  - IV infusion of study drug over 60 (±10) minutes (refer to Section 8.0 and the Study Pharmacy Manual for additional infusion rate guidance). Administration should occur 24 (±2) hours after study drug was administered on the previous day.

    OR
  - Oral step-down therapy.
- Clinical Assessments:
  - A focused physical exam (including a neurological exam) should be performed as clinically indicated by AE screening and course of the underlying disease.
  - Measure vital signs (temperature and the method used [oral, rectal, temporal, tympanic, or core], heart rate, blood pressure, and respiratory rate). Axillary temperatures are allowed, but not preferred.
  - The retinal examination should be repeated in subjects who were negative at baseline if the subject develops visual signs or symptoms of a *Candida* eye infection during the study. Additionally, any neutropenic subjects who recover their neutrophil counts during the study should have an additional retinal examination. Subjects who were negative at baseline and are diagnosed with a *Candida* eye infection after initiating study drug must discontinue study drug (see Section 7.4.2) but may continue in the study at the Investigator's discretion; all subsequent antifungal drugs, interventions, and outcomes for endophthalmitis or chorioretinitis will be recorded.

- Laboratory Assessments:
  - Draw blood or normally sterile tissue/fluid (if possible) for culture if demonstrating mycological eradication (if not already done) or if clinically indicated.
    - Perform identification and susceptibility testing for *Candida* for any positive culture requiring a change of antifungal therapy (i.e., identification and susceptibility testing not required for *Candida* isolates cultured from specimens obtained without a required change in antifungal therapy).
    - All fungal isolates cultured from blood and normally sterile tissue/fluid must be sent to the Central Laboratory.
    - Record the spp. for any new bacteria isolated from blood and the spp. and susceptibilities for any new bacteria isolated from any other normally sterile site.
- Record all concomitant medications.
- Record AEs.
- Presence of central line during Screening or placed during the study should be documented with dates and times of insertion and removal.
- Short-term central venous catheters (e.g., peripherally-inserted central catheters, internal jugular, or subclavian central venous catheters) and long-term central venous catheters (e.g., tunneled catheters) are recommended to be removed within 48 hours after diagnosis with candidemia, consistent with IDSA (Pappas 2016) and ESCMID (Cornely 2012; Ullmann 2012) guidelines.
- If radiologic test is performed, record radiologic test type with Findings and Interpretation only if radiologic test provides evidence of invasive candidiasis or evidence of progression, stabilization, improvement, or resolution of invasive candidiasis from previous radiographs. Criteria for cure, indeterminate, and failure responses are found in Table 10.

9.2.8 Day 14 (±1 day)

- Administer study drug:
  - IV infusion of study drug over 60 (±10) minutes (refer to Section 8.0 and the Study Pharmacy Manual for additional infusion rate guidance). Administration should occur 24 (±2) hours after study drug was administered on the previous day.

    OR
  - Oral step-down therapy.
- Clinical Assessments:
  - Conduct complete physical examination, including weight.

- Conduct neurological examination as part of the physical examination, as clinically indicated.
- Measure vital signs (temperature and the method used [oral, rectal, temporal, tympanic, or core], heart rate, blood pressure, and respiratory rate). Axillary temperatures are allowed, but not preferred.

The retinal examination should be repeated in subjects who were negative at baseline if the subject develops visual signs or symptoms of a *Candida* eye infection during the study. Additionally, any neutropenic subjects who recover their neutrophil counts during the study should have an additional retinal examination. Subjects who were negative at baseline and are diagnosed with a *Candida* eye infection after initiating study drug must discontinue study drug (see Section 7.4.2) but may continue in the study at the Investigator's discretion; all subsequent antifungal drugs, interventions, and outcomes for endophthalmitis or chorioretinitis will be recorded.

- Assess presence or absence of systemic signs and symptoms and determine which are attributable to candidemia and/or invasive candidiasis.
- Assess for clinical response (Table 9).

Laboratory assessments:

- Draw blood for hematology and chemistry.
- Draw blood or normally sterile tissue/fluid (if possible) for culture if demonstrating mycological eradication (if not already done) or if clinically indicated.
  - Perform identification and susceptibility testing for *Candida* for any positive culture requiring a change of antifungal therapy (i.e., identification and susceptibility testing not required for *Candida* isolates cultured from specimens obtained without a required change in antifungal therapy).
  - All fungal isolates cultured from blood and normally sterile tissue/fluid must be sent to the Central Laboratory.
  - Record the spp. for any new bacteria isolated from blood and the spp. and susceptibilities for any new bacteria isolated from any other normally sterile site.
- <u>For subjects not receiving a Day 15 dose</u>, on Day 14, one blood sample for PK will be collected at any time. Draw blood for PK analysis from the OPPOSITE arm or through an arterial line during the infusion; after the infusion is complete opposite arm or arterial line draws are preferred. For subjects receiving hemodialysis, an attempt should be made to obtain PK samples directly following hemodialysis if still within the required time window.

Record all concomitant medications.

Record AEs.

- Presence of central line during Screening or placed during the study should be documented with dates and times of insertion and removal.
- Short-term central venous catheters (e.g., peripherally-inserted central catheters, internal jugular, or subclavian central venous catheters) and long-term central venous catheters (e.g., tunneled catheters) are recommended to be removed within 48 hours after diagnosis with candidemia, consistent with IDSA (Pappas 2016) and ESCMID (Cornely 2012; Ullmann 2012) guidelines.
- If radiologic test is performed, record radiologic test type with Findings and Interpretation only if radiologic test provides evidence of invasive candidiasis or evidence of progression, stabilization, improvement, or resolution of invasive candidiasis from previous radiographs. Criteria for cure, indeterminate, and failure responses are found in Table 10.

9.2.9 Day 30 (-2 days)

- Clinical Assessments:
  - Conduct complete physical examination.
  - Conduct neurological examination as part of the physical examination, as clinically indicated.
  - Measure vital signs (temperature and the method used [oral, rectal, temporal, tympanic, or core], heart rate, blood pressure, and respiratory rate). Axillary temperatures are allowed, but not preferred.
  - The retinal examination should be repeated in subjects who were negative at baseline if the subject develops visual signs or symptoms of a *Candida* eye infection during the study. Additionally, any neutropenic subjects who recover their neutrophil counts during the study should have an additional retinal examination.
  - Assess presence or absence of systemic signs and symptoms and determine which are attributable to candidemia and/or invasive candidiasis.
  - Assess for clinical response (Table 9).
  - Assess ACM.
- Laboratory assessments:
  - Draw blood for hematology and chemistry.
  - Draw blood or normally sterile tissue/fluid (if possible) for culture if demonstrating mycological eradication (if not already done) or if clinically indicated.
    - Perform identification and susceptibility testing for *Candida* for any positive culture requiring a change of antifungal therapy (i.e., identification and susceptibility testing not required for *Candida* isolates cultured from specimens obtained without a required change in antifungal therapy).

- All fungal isolates cultured from blood and normally sterile tissue/fluid must be sent to the Central Laboratory.
- Record the spp. for any new bacteria isolated from blood and the spp. and susceptibilities for any new bacteria isolated from any other normally sterile site.

- Record all concomitant medications.
- Record AEs.
- Presence of central line during Screening or placed during the study should be documented with dates and times of insertion and removal.
- Short-term central venous catheters (e.g., peripherally-inserted central catheters, internal jugular, or subclavian central venous catheters) and long-term central venous catheters (e.g., tunneled catheters) are recommended to be removed within 48 hours after diagnosis with candidemia, consistent with IDSA (Pappas 2016) and ESCMID (Cornely 2012; Ullmann 2012) guidelines.
- If radiologic test is performed, record radiologic test type with Findings and Interpretation only if radiologic test provides evidence of invasive candidiasis or evidence of progression, stabilization, improvement, or resolution of invasive candidiasis from previous radiographs. Criteria for cure, indeterminate, and failure responses are found in Table 10.

9.2.10 End of Treatment (≤2 days of last dose)

- Clinical Assessments:
  - Assess for clinical response (Table 9).
  - Conduct complete physical examination.
  - Conduct a thorough neurological examination as part of the physical examination.
  - Measure vital signs (temperature and the method used [oral, rectal, temporal, tympanic, or core], heart rate, blood pressure, and respiratory rate). Axillary temperatures are allowed, but not preferred.
  - The retinal examination should be repeated in subjects who were negative at baseline if the subject develops visual signs or symptoms of a *Candida* eye infection during the study. Additionally, any neutropenic subjects who recover their neutrophil counts during the study should have an additional retinal examination.
- Laboratory assessments:
  - Draw blood for hematology and chemistry.

- Collect urine for urinalyses, microscopy. Subjects who are anuric do not need to complete this assessment. Urine microscopy is only required in the presence of abnormal urine chemistry tests.
- Draw blood or normally sterile tissue/fluid (if possible) for culture if demonstrating mycological eradication (if not already done) or if clinically indicated.
  - Perform identification and susceptibility testing for *Candida* for any positive culture requiring a change of antifungal therapy (i.e., identification and susceptibility testing not required for *Candida* isolates cultured from specimens obtained without a required change in antifungal therapy).
  - All fungal isolates cultured from blood and normally sterile tissue/fluid must be sent to the Central Laboratory.
  - Record the spp. for any new bacteria isolated from blood and the spp. and susceptibilities for any new bacteria isolated from any other normally sterile site.

- Record all concomitant medications.
- Record AEs.
- Presence of central line during Screening or placed during the study should be documented with dates and times of insertion and removal.
- Short-term central venous catheters (e.g., peripherally-inserted central catheters, internal jugular, or subclavian central venous catheters) and long-term central venous catheters (e.g., tunneled catheters) are recommended to be removed within 48 hours after diagnosis with candidemia, consistent with IDSA (Pappas 2016) and ESCMID (Cornely 2012; Ullmann 2012) guidelines.
- If radiologic test is performed, record radiologic test type with Findings and Interpretation only if radiologic test provides evidence of invasive candidiasis or evidence of progression, stabilization, improvement, or resolution of invasive candidiasis from previous radiographs. Criteria for cure, indeterminate, and failure responses are found in Table 10.

  NOTE: The EOT visit may be performed at the same time as the Day 14 or Day 30 visit if completed within 2 calendar days of the last dose.

9.3 Follow-up (Days 52–59)

- Clinical Assessments:
  - Conduct complete physical examination, including weight.

  - Conduct neurological examination as part of the physical examination, as clinically indicated.
  - Measure vital signs (temperature and the method used [oral, rectal, temporal, tympanic, or core], heart rate, blood pressure, and respiratory rate). Axillary temperatures are allowed, but not preferred.
  - The retinal examination should be repeated in subjects who were negative at baseline if the subject develops visual signs or symptoms of a *Candida* eye infection during the study. Additionally, any neutropenic subjects who recover their neutrophil counts during the study should have an additional retinal examination.
  - Assess presence or absence of systemic signs and symptoms and determine which are attributable to candidemia and/or invasive candidiasis.
  - Assess for clinical response (Table 9).
  - Assess ACM.
- Laboratory assessments:
  - Draw blood for hematology and chemistry.
  - Conduct serum pregnancy test (women of childbearing potential only; do not perform for women ≥2 years postmenopausal or surgically sterile).
  - Draw blood or normally sterile tissue/fluid (if possible) for culture only if clinically indicated.
    - Perform identification and susceptibility testing for *Candida* for any positive culture requiring a change of antifungal therapy (i.e., identification and susceptibility testing not required for *Candida* isolates cultured from specimens obtained without a required change in antifungal therapy).
    - All fungal isolates cultured from blood and normally sterile tissue/fluid must be sent to the Central Laboratory.
    - Record the spp. for any new bacteria isolated from blood and the spp. and susceptibilities for any new bacteria isolated from any other normally sterile site.
- Record all concomitant medications.
- Record AEs.
- If radiologic test is performed, record radiologic test type with Findings and Interpretation only if radiologic test provides evidence of invasive candidiasis or evidence of progression, stabilization, improvement, or resolution of invasive candidiasis from previous radiographs. Criteria for cure, indeterminate, and failure responses are found in Table 10.

9.4 Optional Treatment Period

9.4.1 Days 15 (±1 day)

- One blood sample for PK will be collected pre-dose (≤30 minutes prior to the start of the infusion). Draw blood for PK analysis from the OPPOSITE arm or through an arterial line during the infusion; after the infusion is complete, opposite arm or arterial line draws are preferred. For subjects receiving hemodialysis, an attempt should be made to obtain PK samples directly following hemodialysis if still within the required time window.
- Administer study drug:
  - IV infusion of study drug over 60 (±10) minutes (refer to Section 8.0 and the Study Pharmacy Manual for additional infusion rate guidance). Administration should occur 24 (±2) hours after study drug was administered on the previous day.

    OR
  - IV infusion of study drug over 60 (±10) minutes (refer to Section 8.0 and the Study Pharmacy Manual for additional infusion rate guidance). Administration should occur 24 (±2) hours after study drug was administered on the previous day PLUS oral step-down therapy.
- Clinical Assessments:
  - Conduct complete physical examination.
  - Conduct neurological examination as part of the physical examination, as clinically indicated.
  - Measure vital signs (temperature and the method used [oral, rectal, temporal, tympanic, or core], heart rate, blood pressure, and respiratory rate). Axillary temperatures are allowed, but not preferred.
  - The retinal examination should be repeated in subjects who were negative at baseline if the subject develops visual signs or symptoms of a *Candida* eye infection during the study. Additionally, any neutropenic subjects who recover their neutrophil counts during the study should have an additional retinal examination. Subjects who were negative at baseline and are diagnosed with a *Candida* eye infection after initiating study drug must discontinue study drug (see Section 7.4.2) but may continue in the study at the Investigator's discretion; all subsequent antifungal drugs, interventions, and outcomes for endophthalmitis or chorioretinitis will be recorded.
- Laboratory Assessments:
  - Draw blood or normally sterile tissue/fluid (if possible) for culture if demonstrating mycological eradication (if not already done) or if clinically indicated.

  - Perform identification and susceptibility testing for *Candida* for any positive culture requiring a change of antifungal therapy (i.e., identification and susceptibility testing not required for *Candida* isolates cultured from specimens obtained without a required change in antifungal therapy).
  - All fungal isolates cultured from blood and normally sterile tissue/fluid must be sent to the Central Laboratory.
  - Record the spp. for any new bacteria isolated from blood and the spp. and susceptibilities for any new bacteria isolated from any other normally sterile site.
- Record all concomitant medications.
- Record AEs.
- Presence of central line during Screening or placed during the study should be documented with dates and times of insertion and removal.
- Short-term central venous catheters (e.g., peripherally-inserted central catheters, internal jugular, or subclavian central venous catheters) and long-term central venous catheters (e.g., tunneled catheters) are recommended to be removed within 48 hours after diagnosis with candidemia, consistent with IDSA (Pappas 2016) and ESCMID (Cornely 2012; Ullmann 2012) guidelines.
- If radiologic test is performed, record radiologic test type with Findings and Interpretation only if radiologic test provides evidence of invasive candidiasis or evidence of progression, stabilization, improvement, or resolution of invasive candidiasis from previous radiographs. Criteria for cure, indeterminate, and failure responses are found in Table 10.

9.4.2 Days 16–21

- Administer study drug:
  - IV infusion of study drug over 60 (±10) minutes (refer to Section 8.0 and the Study Pharmacy Manual for additional infusion rate guidance); administration should occur 24 (±2) hours after study drug was administered on the previous day.

    OR
  - Oral step-down therapy.
- Clinical Assessments:
  - A focused physical exam (including a neurological exam) should be performed as clinically indicated by AE screening and course of the underlying disease.
  - Measure vital signs (temperature and the method used [oral, rectal, temporal, tympanic, or core], heart rate, blood pressure, and respiratory rate). Axillary temperatures are allowed, but not preferred.

  - The retinal examination should be repeated in subjects who were negative at baseline if the subject develops visual signs or symptoms of a *Candida* eye infection during the study. Additionally, any neutropenic subjects who recover their neutrophil counts during the study should have an additional retinal examination. Subjects who were negative at baseline and are diagnosed with a *Candida* eye infection after initiating study drug must discontinue study drug (see Section 7.4.2) but may continue in the study at the Investigator's discretion; all subsequent antifungal drugs, interventions, and outcomes for endophthalmitis or chorioretinitis will be recorded.

- Laboratory Assessments:
  - Draw blood or normally sterile tissue/fluid (if possible) for culture if demonstrating mycological eradication (if not already done) or if clinically indicated.
    - Perform identification and susceptibility testing for *Candida* for any positive culture requiring a change of antifungal therapy (i.e., identification and susceptibility testing not required for *Candida* isolates cultured from specimens obtained without a required change in antifungal therapy).
    - All fungal isolates cultured from blood and normally sterile tissue/fluid must be sent to the Central Laboratory.
    - Record the spp. for any new bacteria isolated from blood and the spp. and susceptibilities for any new bacteria isolated from any other normally sterile site.
- Record all concomitant medications.
- Record AEs.
- Presence of central line during Screening or placed during the study should be documented with dates and times of insertion and removal.

- Short-term central venous catheters (e.g., peripherally-inserted central catheters, internal jugular, or subclavian central venous catheters) and long-term central venous catheters (e.g., tunneled catheters) are recommended to be removed within 48 hours after diagnosis with candidemia, consistent with IDSA (Pappas 2016) and ESCMID (Cornely 2012; Ullmann 2012) guidelines.

- If radiologic test is performed, record radiologic test type with Findings and Interpretation only if radiologic test provides evidence of invasive candidiasis or evidence of progression, stabilization, improvement, or resolution of invasive candidiasis from previous radiographs. Criteria for cure, indeterminate, and failure responses are found in Table 10.

9.4.3 Day 22 (±1 day)

- One blood sample for PK will be collected pre-dose (≤30 minutes prior to the start of the infusion). Draw blood for PK analysis from the OPPOSITE arm or through an arterial line during the infusion; after the infusion is complete opposite arm or arterial line draws are preferred. For subjects receiving hemodialysis, an attempt should be made to obtain PK samples directly following hemodialysis if still within the required time window.
- Administer study drug:
  - IV infusion of study drug over 60 (±10) minutes (refer to Section 8.0 and the Study Pharmacy Manual for additional infusion rate guidance). Administration should occur 24 (±2) hours after study drug was administered on the previous day.

    OR

  - IV infusion of study drug over 60 (±10) minutes (refer to Section 8.0 and the Study Pharmacy Manual for additional infusion rate guidance). Administration should occur 24 (±2) hours after study drug was administered on the previous day PLUS oral step-down therapy.
- Clinical Assessments:
  - Conduct complete physical examination.
  - Conduct neurological examination as part of the physical examination, as clinically indicated.
  - Measure vital signs (temperature and the method used [oral, rectal, temporal, tympanic, or core], heart rate, blood pressure, and respiratory rate). Axillary temperatures are allowed, but not preferred.
  - The retinal examination should be repeated in subjects who were negative at baseline if the subject develops visual signs or symptoms of a *Candida* eye infection during the study. Additionally, any neutropenic subjects who recover their neutrophil counts during the study should have an additional retinal examination. Subjects who were negative at baseline and are diagnosed with a *Candida* eye infection after initiating study drug must discontinue study drug (see Section 7.4.2) but may continue in the study at the Investigator's discretion; all subsequent antifungal drugs, interventions, and outcomes for endophthalmitis or chorioretinitis will be recorded.
- Laboratory Assessments:
  - Draw blood or normally sterile tissue/fluid (if possible) for culture if demonstrating mycological eradication (if not already done) or if clinically indicated.
    - Perform identification and susceptibility testing for *Candida* for any positive culture requiring a change of antifungal therapy (i.e., identification and susceptibility testing not required for *Candida* isolates cultured from specimens obtained without a required change in antifungal therapy).

- All fungal isolates cultured from blood and normally sterile tissue/fluid must be sent to the Central Laboratory.
- Record the spp. for any new bacteria isolated from blood and the spp. and susceptibilities for any new bacteria isolated from any other normally sterile site.

- Record all concomitant medications.
- Record AEs.
- Presence of central line during Screening or placed during the study should be documented with dates and times of insertion and removal.
- Short-term central venous catheters (e.g., peripherally-inserted central catheters, internal jugular, or subclavian central venous catheters) and long-term central venous catheters (e.g., tunneled catheters) are recommended to be removed within 48 hours after diagnosis with candidemia, consistent with IDSA (Pappas 2016) and ESCMID (Cornely 2012; Ullmann 2012) guidelines.
- If radiologic test is performed, record radiologic test type with Findings and Interpretation only if radiologic test provides evidence of invasive candidiasis or evidence of progression, stabilization, improvement, or resolution of invasive candidiasis from previous radiographs. Criteria for cure, indeterminate, and failure responses are found in Table 10.

9.4.4 Days 23–28

- Administer study drug:
  - IV infusion of study drug over 60 (±10) minutes (refer to Section 8.0 and the Study Pharmacy Manual for additional infusion rate guidance). Administration should occur 24 (±2) hours after study drug was administered on the previous day.

    OR

  - Oral step-down therapy.
- Clinical Assessments:
  - A focused physical exam (including a neurological exam) should be performed as clinically indicated by AE screening and course of the underlying disease.
  - Measure vital signs (temperature and the method used [oral, rectal, temporal, tympanic, or core], heart rate, blood pressure, and respiratory rate). Axillary temperatures are allowed, but not preferred.

- The retinal examination should be repeated in subjects who were negative at baseline if the subject develops visual signs or symptoms of a *Candida* eye infection during the study. Additionally, any neutropenic subjects who recover their neutrophil counts during the study should have an additional retinal examination. Subjects who were negative at baseline and are diagnosed with a *Candida* eye infection after initiating study drug must discontinue study drug (see Section 7.4.2) but may continue in the study at the Investigator's discretion; all subsequent antifungal drugs, interventions, and outcomes for endophthalmitis or chorioretinitis will be recorded.

- Laboratory Assessments:
  - Draw blood or normally sterile tissue/fluid (if possible) for culture if demonstrating mycological eradication (if not already done) or if clinically indicated.
    - Perform identification and susceptibility testing for *Candida* for any positive culture requiring a change of antifungal therapy (i.e., identification and susceptibility testing not required for *Candida* isolates cultured from specimens obtained without a required change in antifungal therapy).
    - All fungal isolates cultured from blood and normally sterile tissue/fluid must be sent to the Central Laboratory.
    - Record the spp. for any new bacteria isolated from blood and the spp. and susceptibilities for any new bacteria isolated from any other normally sterile site.
- Record all concomitant medications.
- Record AEs.
- Presence of central line during Screening or placed during the study should be documented with dates and times of insertion and removal.

- Short-term central venous catheters (e.g., peripherally-inserted central catheters, internal jugular, or subclavian central venous catheters) and long-term central venous catheters (e.g., tunneled catheters) are recommended to be removed within 48 hours after diagnosis with candidemia, consistent with IDSA (Pappas 2016) and ESCMID (Cornely 2012; Ullmann 2012) guidelines.

- If radiologic test is performed, record radiologic test type with Findings and Interpretation only if radiologic test provides evidence of invasive candidiasis or evidence of progression, stabilization, improvement, or resolution of invasive candidiasis from previous radiographs. Criteria for cure, indeterminate, and failure responses are found in Table 10.

10.0 ASSESSMENT OF EFFICACY

Efficacy assessments for all subjects occur on Day 5, Day 14 (±1 day), Day 30 (-2 days), EOT (≤2 days of last dose), and the Follow-up visit (Days 52–59).

10.1 Primary Efficacy Outcome

The primary efficacy outcome for the FDA is ACM at Day 30 (-2 days). All attempts will be made to determine the survival status of all subjects at Day 30. However, if it is unknown whether a subject is alive or deceased, the subject will be considered deceased for the primary efficacy outcome.

The primary efficacy outcome for the EMA is global cure (based on clinical cure as assessed by the Investigator, radiological cure [for qualifying invasive candidiasis subjects], and mycological eradication) confirmed by an independent DRC at Day 14 (±1 day). Global response is determined from the clinical response as assessed by the Investigator, radiologic response (for those subjects with invasive candidiasis documented by radiologic/imaging evidence at baseline), and mycological response at Day 5, Day 14 (±1 day), Day 30 (-2 days), EOT (≤2 days of last dose) and Follow-up (Days 52–59) and confirmed by the DRC. The outcome of global response as determined by clinical response, radiological response, and mycological response is in Table 7. An independent blinded DRC will review subject data and confirm the determination of global response. The global response as determined by the DRC will used for analysis. The role of the DRC and procedures for outcome determination are described in the DRC Charter.

Table 7: Global Response

Global Response	Definition		
	**Mycological Response**	**Clinical Response as Assessed by the Investigator**	**Radiological Response****
Cure	Eradication/Presumed Eradication*	Cure	Cure
Failure	Eradication/Presumed Eradication *	Cure	Failure
	Eradication/Presumed Eradication *	Failure	Cure, Failure or Indeterminate
	Eradication/Presumed Eradication *	Indeterminate	Failure
	Failure	Cure, Failure or Indeterminate	Cure, Failure or Indeterminate
	Indeterminate	Failure	Cure, Failure or Indeterminate
	Indeterminate	Cure	Failure
	Indeterminate	Indeterminate	Failure
Indeterminate	Eradication/Presumed Eradication *	Cure	Indeterminate
	Eradication/Presumed Eradication *	Indeterminate	Cure or Indeterminate
	Indeterminate	Cure	Cure or Indeterminate
	Indeterminate	Indeterminate	Cure or Indeterminate

* Presumed eradication is defined only for a culture from a normally sterile site and is not defined for a blood culture.
** For those subjects with invasive candidiasis documented by radiologic/imaging evidence at baseline.

10.2 Secondary Efficacy Outcomes

All-cause mortality at Day 30 (-2 days) is a secondary outcome for the EMA, and global cure at Day 14 (±1 day) is a secondary outcome for the FDA.

Additional secondary efficacy outcome measures are mycological eradication, clinical cure, and radiological cure (for those subjects with invasive candidiasis documented by radiologic/imaging evidence at baseline). Definitions for mycological eradication, clinical cure, and radiological cure are provided below in Section 10.2.1, Section 10.2.2, and Section 10.2.3, respectively.

10.2.1 Mycological Response

Table 8: Mycological Response Definitions

Mycological Response	Definition
Eradication*	• If positive blood culture at baseline:<br>○ The last blood culture drawn on or prior to the day of assessments is negative without a subsequent positive culture from a sample drawn following the first dose of study drug<br>• If positive culture from a normally sterile site at baseline (other than blood):<br>○ *Documented* mycological eradication: most recent culture on or prior to the day of assessment from all normally sterile sites of baseline *Candida* infection (if accessible) is negative and culture was obtained after the initiation of study drug,<br>OR<br>○ *Presumed* mycological eradication: follow-up culture from all normally sterile sites of baseline *Candida* infection is not available (e.g., normally sterile baseline site of *Candida* infection not accessible) or the most recent culture from all normally sterile sites of baseline *Candida* infection obtained after the initiation of study drug is positive, in a subject with a successful clinical outcome as assessed by the Investigator (i.e., did not receive rescue antifungal treatment and has resolution of systemic signs and symptoms of invasive candidiasis that were present at baseline) and the subject has a successful radiological outcome (for those with documented evidence of disease from imaging at baseline),<br>AND<br>• There was no change of antifungal therapy for the treatment of candidemia and/or invasive candidiasis,<br>AND<br>• The subject is not lost to follow up on the day of assessment.

Mycological Response	Definition
Failure	• If positive blood culture at baseline:<br>o The last blood culture drawn on or prior to the day of assessment is positive for *Candida* spp. from a sample drawn following the first dose of study drug,<br>OR<br>• If positive culture from a normally sterile site at baseline:<br>o *Documented* mycological persistence: most recent culture on or prior to the day of assessment from all normally sterile sites of baseline *Candida* infection (if accessible) is positive and culture was obtained after the initiation of study drug,<br>OR<br>o *Presumed* mycological persistence: follow-up culture from all normally sterile sites of baseline *Candida* infection is not available (e.g., normally sterile baseline site of *Candida* infection not accessible) OR the most recent culture from all normally sterile sites of baseline *Candida* infection obtained after initiation of study drug is positive in a subject without a successful clinical outcome as assessed by the Investigator or without a successful radiological outcome for those with documented evidence of disease from imaging at baseline,<br>OR<br>• The subject requires a change of antifungal therapy to treat candidemia and/or invasive candidiasis,<br>OR<br>• The subject dies of any cause prior to or on the day of assessment.
Indeterminate	Study data are not available for the evaluation of efficacy for any reason including:<br>• If positive blood culture at baseline: A post-baseline blood specimen was not available to culture or the result was not available.<br>• If positive culture from a normally sterile site at baseline: A sterile site/fluid post-baseline specimen was not available to culture or the result was not available AND an assessment clinical outcome by the Investigator was not available or radiographic assessments are not available.<br>• Subject is lost to follow-up on the day of assessment.

* Presumed eradication is defined only for a culture from a normally sterile site and is not defined for a blood culture.

10.2.2 Clinical Response

Table 9: Investigator's Assessment of Clinical Response Definitions

Clinical Response	Definition
Cure	• Resolution of attributable systemic signs and symptoms of candidemia and/or invasive candidiasis that were present at baseline,<br>AND<br>• No new systemic signs or symptoms attributable to candidemia and/or invasive candidiasis,<br>AND<br>• No new systemic antifungal therapy to treat candidemia and/or invasive candidiasis,<br>AND<br>• The subject is alive.
Failure	• Progression or recurrence of attributable systemic signs or symptoms of candidemia and/or invasive candidiasis,<br>OR<br>• Lack of resolution of attributable systemic signs or symptoms of candidemia and/or invasive candidiasis,<br>OR<br>• Requirement for new systemic antifungal or prolonged therapy to treat candidemia and/or invasive candidiasis *,<br>OR<br>• An AE requires discontinuation of study drug therapy (IV and IV/oral) on or prior to the day of assessment,<br>OR<br>• The subject died of any cause.
Indeterminate	• Study data are not available for the evaluation of efficacy for any reason including:<br>○ Lost to follow-up,<br>○ Withdrawal of consent,<br>○ Extenuating circumstances that preclude the classification of clinical outcome of candidemia and/or invasive candidiasis.

Abbreviations: AE = adverse event; IV = intravenous.
* Prolonged antifungal therapy is defined as therapy for the treatment of candidemia and/or invasive candidiasis extending beyond the allowable 28 days of study drug. The determination of prolonged therapy will only apply to the Follow-up visit clinical response assessment.

10.2.3 Radiological Response

Radiological response is defined in Table 10. Only subjects with invasive candidiasis, with radiologic or other imaging studies at baseline that demonstrate evidence of invasive candidiasis, should be assessed for this outcome.

Table 10: Radiological Response Definitions

Radiological Response	Definition [a]
Cure	• Improvement or resolution of radiological or other imaging findings of invasive candidiasis that were present at baseline (i.e., the radiograph/imaging study that documented evidence of the invasive candidiasis)<br>AND<br>• No new radiological or other imaging findings attributable to invasive candidiasis,<br>AND<br>• The subject is alive.
Failure	• Progression of or new radiological or other imaging findings of invasive candidiasis,<br>OR<br>• Lack of improvement of radiological or other imaging findings of invasive candidiasis,<br>OR<br>• The subject died of any cause.
Indeterminate	• Radiological or imaging data are not available for the evaluation of efficacy for any reason including:<br>o Lost to follow-up,<br>o Withdrawal of consent,<br>o Radiology/Imaging not completed<br>o Extenuating circumstances that preclude the classification of a radiological outcome of invasive candidiasis

[a] Includes radiological or other imaging studies. Only for invasive candidiasis subjects with imaging performed at baseline who had radiological or other imaging studies that documents evidence of invasive candidiasis.

10.3 Exploratory Outcomes

An exploratory efficacy outcome measure is resolution of attributable systemic signs of candidemia and/or invasive candidiasis that were present at baseline. These include fever, hypothermia, hypotension, tachycardia, tachypnea, and local signs of inflammation (i.e., erythema, edema, heat and pain at the site of the qualifying culture).

Health economics outcomes research (HEOR) variables are also exploratory outcomes. These include total length of hospital stay and length of ICU stay.

11.0 ASSESSMENT OF PHARMACOKINETIC/PHARMACODYNAMIC PARAMETERS

Blood samples will be collected from subjects to evaluate the PK of Rezafungin for Injection. Blood samples will be collected from all subjects, but only PK samples from subjects receiving Rezafungin for Injection will be analyzed. Plasma samples will be analyzed for the concentration of rezafungin at a central bioanalytical laboratory using a validated liquid chromatography-tandem mass spectrometry method. Samples from this study may also be included in a population PK analysis, which will be reported separately.

In addition, rezafungin plasma protein binding and albumin concentrations will be determined from pre-dose plasma samples from subjects enrolled prior to amendment 5, and reported separately.

Procedures for collection, storage, and shipping of PK samples are described in Appendix 8 and in the Pharmacokinetics Manual.

12.0 ASSESSMENT OF SAFETY

12.1 Safety Parameters

Safety will be assessed through the evaluation of AEs, vital signs (temperature, heart rate, blood pressure, and respiratory rate), physical examinations (including neurological examinations), ECGs, and clinical laboratory data (clinical chemistry panels, hematology evaluations, and urinalyses). Subjects will also be assessed at Screening and at the EOT visit, prior to Rezafungin for Injection dosing, with a thorough neurologic evaluation to assess for signs and symptoms of tremor, ataxia, and peripheral neuropathy. Adverse events and abnormal laboratory values will be graded for severity using the National Cancer Institute CTCAE version 5.0 (Table 12) and coded using the Medical Dictionary for Regulatory Activities (MedDRA).

12.2 Adverse Events

Adverse Events will be collected for all subjects from informed consent through Follow-up (Days 52–59). The Investigator will assess all AEs and SAEs and will record the following information on the appropriate eCRF page:

- Date of onset
- Date of resolution or stabilization
- Seriousness
- AE severity
- Causal relationship to study drug(s), including relationship to a specific study drug if a determination can be made
- Action taken with study medication

Medically indicated laboratory tests (emergency or unscheduled tests) should be conducted at the local laboratory. The Investigator should employ best medical judgment in determining how to manage AEs and SAEs. Any questions regarding AE or SAE management should be directed to the Medical Monitor.

The Sponsor is responsible for:

- Confirming the seriousness assessment of all reported AEs and SAEs
- Confirming the causal relationship between reported AEs/SAEs and the investigational product
- Assessment of SAEs for expectedness

The Sponsor will continuously assess the safety of the investigational product throughout the study.

12.3 Adverse Event Reporting

12.3.1 Notification of Serious Adverse Events

The Sponsor has requirements for expedited reporting of SAEs meeting specific criteria to worldwide regulatory authorities in accordance with ICH guidelines and local regulatory requirements. Therefore, the Investigator must notify the Sponsor immediately regarding any SAE that occurs after informed consent through the final follow-up visit (Days 52–59) via transmission of a Serious Adverse Event Report (SAER) by the study site to the safety vendor by email within 24 hours of awareness that an SAE has occurred, with a copy to the Medical Monitor. Contact details will be provided to all sites. An optional initial report can be made via telephone, but a completed SAER must still be emailed within 24 hours of the site's knowledge of the event.

The investigational site will be provided with SAER forms wherein the following information is requested:

- Subject identification, Investigator name, and site number
- SAE information: event term, onset date, AE severity, and causal relationship
- The outcomes attributable to the event (e.g., death, a life-threatening AE, inpatient hospitalization, prolongation of existing hospitalization, a persistent or significant disability or incapacity, or other important medical event[s])
- A summary of relevant test results, pertinent laboratory data, and any other relevant medical history
- The first and last dates of study drug administration (NOTE: as this is a double-blind study, SAERs should not indicate specific study drug assignments)
- Indicate if the study drug was discontinued or the study drug administration schedule modified

Supplemental information may be requested from the site, including hospital records, laboratory results, radiology reports, progress notes, admission and emergency room notes, holding and observation notes, discharge summaries, autopsy reports, and death certificates.

Relevant case report form (CRF) pages can be appended to communicate information relevant to the SAE, including study drug administration details, medical history, concomitant medications, relevant laboratory test results and subject outcome information. The SAER should be emailed within 24 hours with as much of the above information as available at the time. The following minimum information is required for reporting an SAE: subject identification, reporting source, and an event or outcome. SAEs are to be reported immediately and under no circumstance should this reporting time exceed 24 hours following knowledge of the SAE. Supplemental information may be transmitted using a follow-up report and should not delay the initial report. The Sponsor may contact the investigational site to solicit additional information or follow up on the event.

The Investigator must take all therapeutic measures necessary for resolution of the SAE. Any medications or procedures necessary for treatment of the SAE must be recorded on the appropriate pages of the subject's eCRF.

If the Investigator becomes aware of any SAEs that occur after the study period that are believed to be causally related to use of the investigational product, they should notify the Sponsor immediately.

The Sponsor will report all suspected unexpected serious adverse reactions (SUSARs) within the required regulatory timeframes to all applicable regulatory authorities and to Investigators as required.

All SAEs that do not meet expedited reporting requirements will be summarized in the Development Safety Update Report (DSUR) and reported annually by the Sponsor as required.

12.3.2 Notification of Adverse Events of Special Interest

The Investigator should also alert the Sponsor of any AESI (as described in Section 12.7) that occurs on the study within 24 hours of awareness by recording the information in the AE eCRF, even if the nature of the AE is deemed non-serious according to the usual regulatory criteria.

12.3.3 Notification of Emerging Safety Issues

Additionally, the Investigator should alert the Sponsor immediately (under no circumstance should this reporting time exceed 24 hours) by contacting the Medical Monitor of any new findings that necessitate the implementation of urgent safety measures to protect subjects against any immediate hazard. If time permits, the Investigator should contact the Sponsor to discuss the hazard and any actions taken or to be taken prior to implementation.

The Sponsor is responsible for informing other investigators who may need to implement the same procedures and for alerting the Competent Authorities and Ethics Committees of the new findings and measures taken in accordance with local regulations within the required timeframes.

12.4 Definitions

12.4.1 Adverse Event

An AE means any untoward medical occurrence associated with the use of a drug in humans, whether or not considered drug related. An AE (also referred to as an adverse experience) can be any unfavorable and unintended sign (e.g., a clinically-significant abnormal laboratory finding), symptom, or disease temporally associated with the use of a drug and does not imply any judgment about causality. An AE can arise with any use of the drug (e.g., off-label use, use in combination with another drug) and with any route of administration, formulation, or dose, including an overdose.

Events that are related to the disease under study, candidemia or invasive candidiasis, should not be considered or recorded as AEs unless the event fits the definition for an SAE (Section 12.4.2), in which case the SAE form must be submitted for safety reporting in the appropriate timeframe and entered as an AE into the eCRF.

Laboratory abnormalities should not be recorded as AEs or SAEs unless they are associated with clinical signs or symptoms, or require medical intervention. However, each laboratory abnormality (e.g., clinically significant changes detected on hematology, comprehensive metabolic panel, urinalysis) independent from any underlying medical condition that requires medical or surgical intervention, or that leads to interruption of study drug infusion or discontinuation, must be recorded as an AE, or SAE if applicable. If the laboratory abnormality is part of a clinical condition or syndrome, it should be recorded as the syndrome or diagnosis rather than as the individual laboratory abnormality. In addition, laboratory abnormalities or other abnormal test assessments (e.g., ECGs) that are associated with signs or symptoms must be recorded as AEs or SAEs if they meet the definition of an AE or SAE as described in Section 12.4.1 and Section 12.4.4, respectively.

12.4.2 Suspected Adverse Reaction

A suspected adverse reaction is any AE for which there is a reasonable possibility that the drug caused the AE. For the purposes of Investigational New Drug (IND) safety reporting, ‘‘reasonable possibility’’ means there is evidence to suggest a causal relationship between the drug and the AE. Suspected adverse reaction implies a lesser degree of certainty about causality than adverse reaction, which means any AE caused by a drug.

12.4.3 Life-Threatening AE or Life-Threatening Suspected Adverse Reaction

An AE or suspected adverse reaction is considered ‘‘life threatening’’ if, in the view of either the Investigator or Sponsor, its occurrence places the patient or subject at immediate risk of death. It does not include an AE or suspected adverse reaction that, had it occurred in a more severe form, might have caused death.

12.4.4 Serious Adverse Event or Serious Suspected Adverse Reaction

An AE or suspected adverse reaction is considered ‘‘serious’’ if, in the view of either the Investigator or Sponsor, it results in any of the following outcomes:

- Death
- Life-threatening AE – see definition above
- Inpatient hospitalization or prolongation of existing hospitalization
- A persistent or significant incapacity or substantial disruption of the ability to conduct normal life functions
- A congenital anomaly/birth defect in the offspring of a subject who received study drug

- Important medical events that may not result in death, be life-threatening, or require hospitalization may be considered serious when, based upon appropriate medical judgment, they may jeopardize the patient or subject and may require medical or surgical intervention to prevent one of the outcomes listed in this definition. The EudraVigilance Expert Working Group has coordinated the development of an Important Medical Event Terms (IME) list that is intended for guidance purposes (European Medicines Agency 2019). Examples of such medical events include:
    - Blood dyscrasias
    - Convulsions that do not result in inpatient hospitalization
    - Development of drug dependency or drug abuse

12.4.5 Unexpected AE or Unexpected Suspected Adverse Reaction

An AE or suspected adverse reaction is considered ‘‘unexpected’’:

- If it is not listed in the Investigator’s Brochure or is not listed at the nature, severity, frequency, or outcome that has been observed
- If an Investigator’s Brochure is not required or available, is not consistent with the risk information described in the general investigational plan or elsewhere in the current application, as amended
- If it is not listed in the Prescribing Information (for marketed products)

For example, under this definition, hepatic necrosis would be unexpected (by virtue of greater severity) if the Investigator’s Brochure referred only to elevated hepatic enzymes or hepatitis. Similarly, cerebral thromboembolism and cerebral vasculitis would be unexpected (by virtue of greater specificity) if the Investigator’s Brochure listed only cerebral vascular accidents.

‘‘Unexpected,’’ as used in this definition, also refers to AEs or suspected adverse reactions that are mentioned in the Investigator’s Brochure as occurring with a class of drugs or as anticipated from the pharmacological properties of the drug, but are not specifically mentioned as occurring with the particular drug under investigation.

12.4.6 Emerging Safety Issue

Any new safety information that may lead to a reassessment of the risk/benefit balance of the investigational product and/or impact subjects’ health. Examples include:

- Any new safety issue relating to the conduct of the clinical trial that may impact the safety of the trial subjects such as significant safety results from a recently-completed non-clinical study or early termination or temporary suspension of a trial for safety reasons that is conducted on the same investigational product

- Recommendations from the DSMB that may affect the safety of trial subjects

12.4.7 Urgent Safety Measure

An urgent safety measure (USM) is a procedure not defined by the protocol that can be put in place with immediately without prior authorization from Ethics Committees or Regulatory Authorities in order to protect study participants from any immediate hazard to their health and safety.

12.5 Adverse Event Classification

12.5.1 Relationship to Investigational Drug

The Investigator's assessment of causality must be provided for all AEs (serious and non-serious) (Table 11). An Investigator's causality assessment is the determination of whether there exists a reasonable possibility that the study drug caused or contributed to an AE.

Clinical failure that qualifies as an AE without evidence of study drug toxicity should be considered unrelated to study drug.

These criteria, in addition to good clinical judgment, should be used as a guide for determining the causal assessment. If the event is thought to be unrelated to study drug administration, then an alternative explanation should be provided.

Table 11: Guidelines for Assessing Relationship of Event to Study Drug

Unrelated	There is little or no chance that the Investigational Product caused the AE; other conditions, including concurrent illnesses, progression or expression of the disease state, or a reaction to a concomitant medication best explain the event
Related	The association of the AE with the Investigational Product is unknown; however, the AE is not clearly due to another condition, or a reasonable temporal association exists between the AE and treatment administration and, based on the Investigator's clinical experience, the association of the AE with the Investigational Product seems likely

Abbreviation: AE = adverse event.

12.5.2 Severity

All AEs will be graded for severity using the CTCAE grading criteria (version 5.0). The Investigator will assign a grade to the AE using the CTCAE definitions for Grade 1, Grade 2, Grade 3, Grade 4, or Grade 5.

Table 12: Guidelines for Severity Assessment by the CTCAE Version 5.0

	Definition
Grade 1	Mild; asymptomatic or mild symptoms; clinical or diagnostic observations only; intervention not indicated.
Grade 2	Moderate; minimal, local or noninvasive intervention indicated; limiting age appropriate instrumental ADL.
Grade 3	Severe or medically significant but not immediately life-threatening; hospitalization or prolongation of hospitalization indicated; disabling; limiting self-care ADL.
Grade 4	Life-threatening consequences; urgent intervention indicated.
Grade 5	Death related to AE.

Abbreviations: ADL = activities of daily living; AE = adverse event; CTCAE = Common Terminology Criteria for Adverse Events.

Each CTCAE term may not always be listed as expected. For example, the grading scale for pneumonia is referenced under the CTCAE term "lung infection," for thrombocytopenia under the CTCAE term "platelet count decreased," and for neutropenia under the CTCAE term "neutrophil count decreased."

Each System Organ Class (SOC) category contains an "Other" option. For example, "Immune Systems Disorders Other, specify." When a CTCAE term is not listed under the SOC category, the grading scale for the "Other" option can be referenced to aid in severity assessment.

12.5.3 Serious Adverse Event

Any adverse experience occurring at any dose of study medication that occurs between the time of informed consent and the Follow-up visit (Days 52–59) that results in any of the outcomes listed in Section 12.4.4.

Hospitalization for a planned or elective procedure or surgery for a pre-existing condition that has not worsened is not considered an SAE.

12.6 Adverse Event Follow-up

All unresolved SAEs, AESIs, and study drug-related AEs ("ongoing" at discharge) will be followed by the study staff until resolution or deemed stable, regardless of severity.

12.7 Adverse Events of Special Interest

In addition to SAEs, the following are considered AESIs and should be promptly reported to the Sponsor within 24 hours of awareness by recording the information in the AE eCRF even if the nature of the AE is deemed non-serious according to the usual regulatory criteria. Entry of an AESI into the AE CRF page will trigger the notification to the Sponsor.

12.7.1 Intravenous Infusion Intolerability

Events that, in the opinion of the Investigator, may represent intolerance of the IV infusion of study drug. In general, these events would be temporally associated with the IV infusion of the Sponsor's study drug.

12.7.2 Phototoxicity

Due to results from a nonclinical phototoxicity study in rats and a Phase 1 clinical trial indicating mild photosensitivity with rezafungin use, subjects should be advised to avoid sun and other UV light exposure without adequate protection. Investigators should report any AE potentially related to phototoxicity to the Sponsor.

12.7.3 Ataxia, Neuropathy, and Tremors

In a rezafungin 3-month toxicity study in monkeys, there were observations of tremors, and histology with Schwann cell hypertrophy/hyperplasia (reversible) and phospholipidosis in the dorsal root ganglia first appearing at week 6 of dosing and at 11-fold the exposure for the dosing regimen of Rezafungin for Injection used in this study. Ataxia, axonal neuropathy, hypoesthesia, paresthesia, peripheral motor neuropathy, peripheral neuropathy, peripheral sensory neuropathies, peripheral sensorimotor neuropathy, polyneuropathy, toxic neuropathy, and tremors will be considered AESIs.

12.8 Adverse Event Management

The Investigator should employ best medical judgement in determining how to manage AEs, which may represent intolerability or toxicity to the study drug. Questions regarding AE management should be directed to the Medical Monitor.

12.9 Risks for Women of Childbearing Potential or During Pregnancy

The risks of Rezafungin for Injection in pregnant or lactating women are unknown. Pregnant and lactating female subjects are excluded from this study.

Subjects must be instructed to inform the Investigator immediately if they or their partner becomes pregnant during the study. In the case of a partner pregnancy, partner informed consent must be attained prior to collecting information related to the pregnancy and to allow the Investigator to follow them for the outcome of the pregnancy. In the event of a confirmed pregnancy, the following actions should be taken:

- Study drug should be discontinued immediately in female subjects; male subjects may continue in the case of a partner pregnancy.
- The pregnancy should be reported to the Sponsor within 24 hours of the subject notifying the Investigator using the applicable Pregnancy Reporting Form.

- The Investigator should counsel female subjects regarding the possible effects of Rezafungin for Injection exposure on the fetus prior to study drug discontinuation and the need to inform the study site of the outcome of the pregnancy.
- The subject or subject's partner, if consented, should be monitored until the postnatal day 28 or until termination of the pregnancy. The outcome should be reported using the Pregnancy Reporting Form.

Pregnancy is not an AE, in and of itself. However, any pregnancy complication or elective termination of a pregnancy for medical reasons will be recorded as an AE or SAE.
A spontaneous abortion is always considered an SAE and will be reported as described in the AE and SAE sections. Furthermore, any SAE occurring as an adverse pregnancy outcome post-study must be reported to the Medical Monitor.

12.10 Risks for Males of Childbearing Potential

Recent results from a nonclinical rat fertility study indicated adverse effects on sperm morphology and motility without interference with reproduction were seen at 5.4 times, but not at 2.5 times, the exposure of rezafungin in this study. The risks for rezafungin effects on spermatogenesis in non-human primates and in humans have yet to be defined. Partner pregnancies of male subjects should be followed according to Section 12.9, when possible.

13.0 STATISTICAL METHODS

A Statistical Analysis Plan (SAP) will be prepared and finalized prior to database lock. Any changes to the SAP after it has been signed and prior to the final database lock will be documented in an addendum to the SAP. Any deviations from the final SAP/addendum will be described and justified in the clinical study report. All statistical analyses will be performed using SAS®.

Descriptive statistics, including the numbers and percentages for categorical variables, and the numbers, means, standard deviations, medians, minimums, and maximums for continuous variables, will be provided. Listings of individual subject data will also be produced.

13.1 Analysis Populations

The analysis populations are defined for this study as follows:

- The intent-to-treat (ITT) population will include all randomized subjects, regardless of whether the subject receives study drug.
- The Safety population will include all subjects who received any amount of study drug.
- The mITT population will include all subjects who had documented *Candida* infection based on Central Laboratory evaluation of a culture from blood or another normally sterile site obtained ≤4 days (96 hours) before randomization and received ≥1 dose of study drug.
- The clinically evaluable (CE) population will include all subjects in the mITT population who also met inclusion criterion #4, did not meet exclusion criteria #1, #2, or #5, had an assessment of both mycological and clinical response at Day 14 in the window of Day 14 (±1 day) (subjects with invasive candidiasis documented by radiologic/imaging evidence also must have an assessment of radiologic response), and did not have any other factor that could confound the assessment of the global response at Day 14.
- PK Analysis Population will include all Rezafungin for Injection subjects with at least 1 plasma sample obtained for PK analysis.

13.2 Subject Characteristics

Demographics (including age, race, and sex), diagnosis at randomization (candidemia and/or invasive candidiasis), diagnosis methodology(blood culture/rapid IVD and culture specimen obtained from a normally sterile site), ANC at randomization (<500 cells/µL and ≥500 cells/µL), medical history including subjects diagnosed with COVID-19, *Candida* risk factors, baseline assessments (including height, weight, body mass index [BMI], modified APACHE II score, and Glasgow coma score), mycological data, and systemic signs and symptoms will be summarized in the ITT and mITT populations.

13.3 Study Drug Exposure

Descriptive statistics for the duration of study drug therapy (IV and oral, and separately for IV therapy and oral therapy) will be summarized by treatment group for the mITT and Safety populations. The number and percentage of subjects with 1–7, 8–14, 15–28, and >28 days of exposure will be presented for IV and IV and oral therapy combined. The number and percentage of subjects with 1–3, 4–7, 8–14, 15–28, and >28 days of exposure will be presented for oral therapy. The number and percentage of subjects switching to oral study drug therapy and a frequency distribution of the day of oral switch will be summarized by treatment group for the Safety and mITT populations.

13.4 Efficacy Analyses

For efficacy analyses, subject data will be analyzed in the treatment group to which the subject was randomized.

13.4.1 Primary Efficacy Analyses for the FDA

The primary efficacy outcome for the FDA is ACM at Day 30 (-2 days). The addendum to ICH E9 introduces the concept of an estimand which translates the trial objective into a precise definition of the treatment effect that is to be estimated. The description of the estimand includes four attributes: the population, the variable (or endpoint) to be obtained for each patient, the specification of how to account for intercurrent events (ICE), and the population-level summary for the variable. The estimand attributes for ACM at Day 30 are provided in detail in the SAP.

The number and percentage of subjects in each treatment group who are alive and deceased (or with missing data) at Day 30 will be determined in the mITT population.

The null and alternative hypotheses are the following:

$H0: P1 - P2 \geq \Delta$

$H1: P1 - P2 < \Delta$

Where:

P1 = the primary efficacy outcome in the Rezafungin for Injection group,

P2 = the primary efficacy outcome in the caspofungin group,

Δ = the non-inferiority (NI) margin of 20%.

The NI hypothesis test is a 1-sided hypothesis test performed at the 2.5% level of significance. This is based on the upper limit of the 2-sided 95% CI for the observed difference in the rate of ACM at Day 30 (Rezafungin for Injection group minus the caspofungin group). The primary analysis is based on a CI computed using the unadjusted methodology of Miettinen and Nurminen. If the upper limit of the 95% CI for the difference in rates of ACM at Day 30 in

the mITT population is <20%, the null hypothesis will be rejected and the NI of Rezafungin for Injection to caspofungin will be concluded.

An adjusted 95% CI (adjusted for the randomization stratification factors) will also be computed using the methodology of Miettinen and Nurminen. Cochran-Mantel-Haenszel weights will be used for the stratum weights in the calculation of the CI.

13.4.1.1 Additional Analyses of the FDA Primary Efficacy Outcome

To control the overall alpha level, superiority of rezafungin for ACM at Day 30 will be tested in the following order:

- If NI is declared for ACM at Day 30 in the mITT population and the rate of ACM at Day 30 in the rezafungin treatment group is lower than the caspofungin treatment group, the difference between the treatment groups will be tested for superiority. If the upper bound of the 95% CI is less than 0, superiority of rezafungin will be concluded.
- For the subgroup of subjects with invasive candidiasis, a two-sided 95% CI for the observed difference in the rate of ACM at Day 30 will be calculated using the unadjusted methodology of Miettinen and Nurminen. If superiority is declared in the mITT population for ACM at Day 30 and the rate of ACM at Day 30 in the rezafungin treatment group is lower than the caspofungin group for invasive candidiasis subjects, superiority of rezafungin will be tested. If the upper bound of the 95% CI is less than 0, superiority of rezafungin will be concluded.

All-cause mortality at Day 30 will be assessed separately across the randomization stratification factors of diagnosis (candidemia only; invasive candidiasis) and APACHE II score/ANC (APACHE II score ≥20 OR ANC <500 cells/µL; APACHE II score <20 AND ANC ≥500 cells/µL). For each subgroup, the number and percentage of subjects alive and deceased at Day 30 will be determined by treatment group in the mITT population. A 2-sided 95% CI for the observed difference in rates of ACM at Day 30 will be calculated in the mITT population using the unadjusted methodology of Miettinen and Nurminen. Other subgroup analyses, including analyses by the pre-, peri-, and post-COVID-19 period will be conducted for descriptive purposes. Depending on the number of subjects in the study diagnosed with COVID-19, ACM at Day 30 may be analyzed in patients with and without COVID-19.

A sensitivity analysis of the primary outcome using multiple imputation methods for missing data, as detailed in the SAP, for subjects whose survival status is not known at Day 30 will be conducted.

13.4.2 Primary Efficacy Analyses for the EMA

The primary efficacy outcome for the EMA is global cure at Day 14 (±1 day) based on the DRCs determination. Global response is a composite estimand and ICEs are incorporated into the

variable/endpoint. The estimand attributes for global response are provided in more detail in the SAP.

The number and percentage of subjects in each treatment group who have a global response of cure, failure and indeterminate at Day 14 (±1 day) will be determined in the mITT population.

The null and alternative hypotheses are the following:

H0: $P1 - P2 \leq -\Delta$

H1: $P1 - P2 > -\Delta$

Where:

P1 = the primary efficacy outcome in the Rezafungin for Injection group,

P2 = the primary efficacy outcome in the caspofungin group,

Δ = the NI margin of 20%.

The NI hypothesis test is a 1-sided hypothesis test performed at the 2.5% level of significance. This is based on the lower limit of the adjusted (for the randomization stratification factors) 2-sided 95% CI for the observed difference in global success rate (Rezafungin for Injection group minus the caspofungin group). The primary analysis is based on a CI computed using the method with stratification by Miettinen and Nurminen. Cochran-Mantel-Haenszel weights will be used for the stratum weights in the calculation of the CI. If the lower limit of the 95% CI for the difference in global success rates in the mITT population is >-20%, the null hypothesis will be rejected and the NI of Rezafungin for Injection to caspofungin will be concluded.

To control the overall alpha level, superiority of rezafungin for global response will be tested in the following order:

- If NI is declared and the percentage of subjects that have a global response of cure at Day 14 (±1 day) as confirmed by the DRC in the rezafungin treatment group is higher than the caspofungin treatment group, the difference between the treatment groups will be tested for superiority. If the lower bound of the 95% CI is greater than 0, superiority of rezafungin will be concluded.
- For the subgroup of subjects with invasive candidiasis, a two-sided 95% CI for the observed difference in global cure at Day 14 for the mITT population will be calculated using the unadjusted methodology of Miettinen and Nurminen. If superiority is declared for the EMA primary efficacy outcome of global response at Day 14 in the mITT population, and the global cure rate in the rezafungin treatment group is higher than the caspofungin group for invasive candidiasis subjects, superiority of rezafungin will be

tested. If the lower bound of the 95% CI is greater than 0, superiority of rezafungin will be concluded.

Global response will also be assessed separately across the randomization stratification factors of diagnosis (candidemia only; invasive candidiasis) and APACHE II score/ANC (APACHE II score ≥20 OR ANC <500 cells/μL; APACHE II score <20 AND ANC ≥500 cells/μL). For each subgroup, the number and percentage of subjects with a global response of cure, failure, and indeterminate at Day 14 (±1 day) will be determined by treatment group in the mITT population. A 2-sided 95% CI for the observed difference inglobal cure rates will be calculated in the mITT population using the unadjusted methodology of Miettinen and Nurminen. Other subgroup analyses, including analyses by the pre-, peri- and post-COVID-19 period, will be conducted for descriptive purposes. Depending on the number of subjects in the study diagnosed with COVID-19, global response may be analyzed in patients with and without COVID-19.

The reasons for having a global response of failure or indeterminate at Day 14 (±1 day) will be presented using the number and percentage of subjects displayed by treatment group in the mITT population. Subjects who miss the efficacy assessment due to COVID-19 will have an indeterminate response and the number and percentage of subjects with an indeterminate response due to COVID-19 will be summarized.

An unadjusted 95% CI will also be computed using the methodology of Miettinen and Nurminen. Additional analyses of the primary efficacy outcome for the EMA, including analyses in the ITT and CE populations, will be described in the SAP.

13.4.3 Secondary Efficacy Analyses

All-cause mortality at Day 30 (-2 days) is a secondary outcome for the EMA and global cure at Day 14 (±1 day) as confirmed by DRC is a secondary outcome for the FDA.

The secondary efficacy outcomes are global cure (as confirmed by the DRC), mycological response, radiological response (for subjects with invasive candidiasis documented by radiologic/imaging evidence at baseline), and clinical response. The number and percentage of subjects with a global response of cure, failure, or indeterminate will be presented by treatment group in the mITT population at Day 5, Day 30 (-2 days), EOT (≤2 days of last dose) and Follow-up (Days 52–59). The number and percentage of subjects with a mycological response of eradication, failure, or indeterminate will be presented by treatment group in the mITT population at Day 5, Day 14 (±1 day), Day 30 (-2 days), EOT (≤2 days of last dose), and Follow-up (Days 52–59). The number and percentage of subjects with invasive candidiasis documented by radiologic/imaging evidence with a radiological response of cure, failure, or indeterminate will be presented by treatment group in the mITT population at Day 5, Day 14 (±1 day), Day 30 (-2 days), EOT (≤2 days of last dose), and Follow-up (Days 52–59). The number and percentage of subjects with a clinical response of cure, failure, or indeterminate will be presented by treatment group in the mITT population at Day 5, Day 14 (±1 day), Day 30 (-2 days), EOT (≤2 days of last dose), and Follow-up (Days 52–59). 95% CIs for the treatment

differences in global cure, mycological eradication, radiological cure and clinical cure will be determined.

Analyses of global response at Day 14 (±1 day) will also be conducted in two subsets of subjects defined based on the timing of the culture used to document the *Candida* infection: 1) subjects with a culture from blood drawn within 12 hours prior to randomization or within 72 hours after randomization, OR a culture from another normally sterile site obtained within 48 hours prior to randomization or within 72 hours after randomization, and 2) subjects with a culture from blood drawn within 12 hours prior to randomization or within 72 hours after randomization, OR a culture from another normally sterile site obtained within 96 hours prior to randomization or within 72 hours after randomization. For each subset of subjects, the number and percentage of subjects with a global response of cure, failure, or indeterminate will be presented by treatment group. The 95% CIs for the treatment differences in global cure will be determined.

13.4.4 Additional Efficacy Analyses

The number and percentage of subjects with resolution of all systemic signs attributable at baseline to candidemia and/or invasive candidiasis at Day 5, Day 14 (±1 day), Day 30 (-2 days) and Follow-up (Days 52–59) will be presented by treatment group in the mITT population.

The number and percentage of subjects who died up through the Follow-up visit (ACM) will be presented by treatment group in the mITT population. In addition, Kaplan-Meier (KM) methods will be utilized to analyze survival time in the mITT and ITT populations. Subjects who remain alive or are lost to follow-up will be censored at the last date known to be alive. Kaplan-Meier survival curves will be provided. The 25th, 75th percentiles and the median survival time as well as the probability of being alive at Day 5, Day 14, Day 30, and Day 55 will be determined by treatment group.

The time to the first negative blood culture (for those subjects enrolled with a positive blood culture) without subsequent positive culture from a sample drawn following the first dose of study drug will be summarized using KM methods. Subjects will be censored if they receive an alternative antifungal (i.e., other than study drug) for the treatment of the candidemia, died or were lost to follow up prior to having the negative blood culture. Kaplan-Meier survival curves will be provided. The 25th, 75th percentiles and the median survival time will be determined by treatment group.

13.5 Safety Analyses

All safety analyses will be conducted in the Safety population and will be summarized by study drug actually received.

A TEAE is defined as an AE that occurs during or after the first dose of study drug up through the Follow-up visit (Days 52–59). An overall summary of AEs will be provided by treatment group. The incidence of TEAEs will be presented by SOC and Preferred Term (PT); SOC, PT

and relationship to the study drug administration; and SOC, PT, and CTCAE grade (version 5.0). Tables of any TEAE resulting in discontinuation of study drug and SAEs will also be provided. A table of TEAEs of coronavirus will be presented by PT and a listing of all TEAEs in subjects with COVID-19 (medical history or TEAE) will be provided. Depending upon the number of subjects diagnosed with COVID-19, a summary of TEAEs by SOC and PT may be provided.

Descriptive statistics for clinical laboratory test results (hematology and chemistry) will be presented for all study visits. The change from baseline to each post-baseline visit and to the overall worst post-baseline value will be summarized by treatment group. Laboratory values will be graded using CTCAE version 5.0 and shift tables will be provided. Liver enzymes will be classified as > ULN, >3×, >5× and >10×ULN and the number and percentage of subjects with any post-baseline value in one of the categories will be presented by study drug.

Descriptive statistics of vital sign parameters will be presented by treatment group and study visit, as well as the change from baseline at each study visit. Potentially clinically significant (PCS) values will be defined in the SAP and the number and percentage of subjects with a PCS value will be summarized by treatment group for the worst-post-baseline value.

Descriptive statistics of ECG parameters will be presented by treatment group and will include change from baseline.

13.6 Health Economics Outcome Research

Descriptive statistics of the total length of hospital stay and the total length of ICU stay will be provided by treatment group.

13.7 Pharmacokinetic Analyses

Samples from this study may be included in a population PK analysis, which will be reported separately. In addition, rezafungin plasma protein binding and albumin concentrations will be determined from pre-dose plasma samples from subjects enrolled prior to amendment 5, and reported separately. The PK parameters that will be assessed in the PK Analysis Population include: C_{max} and minimum plasma concentration (C_{min}).

13.8 Determination of Study Sample Size

For the EMA endpoint of global cure (confirmed by the DRC), using a 20% NI margin, one-sided alpha of 0.025, 80% power, 1:1 randomization, a global cure rate of 70% in both the Rezafungin for Injection and caspofungin groups, and the sample size methodology based on a continuity corrected Z-statistic, a total of 184 subjects (92 subjects in each treatment group) are required in the mITT population. Assuming 85% of subjects will be evaluable for the mITT population, a total of approximately 218 subjects will be randomized.

For the FDA endpoint of ACM at Day 30 (-2 days), using a 20% NI margin, one-sided alpha of 0.025, an ACM at Day 30 rate of 20% in both treatment groups, 1:1 randomization, and the

sample size methodology based on a continuity corrected Z-statistic, a total of 184 subjects in the mITT population provides an 89.7% power to show NI.

Thus, a total of 218 randomized subjects provides sufficient power for the primary efficacy analyses for both the FDA and EMA in the mITT population.

13.9 Data and Safety Monitoring Board

An independent external DSMB will be established to review safety data. The DSMB will review safety data when approximately 50% of subjects are randomized and as required for AEs of special interest as provided in the DSMB Charter. The DSMB will be comprised of clinicians and a statistician who are experienced in studies of fungal infections. A detailed charter will be developed, compliant with guidelines of the FDA and the ICH that describes the roles and responsibilities of the DSMB, the timing of the meetings, as well as the data to be provided for review.

13.10 Handling of Dropouts and Missing, Unused, and Spurious Data

Every effort will be made to collect all data at specified times. For primary outcome measure (FDA), if the subject's survival status is unknown at Day 30, the subject will be assumed deceased. A sensitivity analysis of the primary outcome (FDA) will be conducted where missing data are imputed using a multiple imputation analysis. For the primary outcome measure for global cure for the EMA, missing data are considered an indeterminate response and are included in the denominator and are thus, are analyzed in the same manner as global failures. Sensitivity analyses of the EMA primary endpoint to assess the impact of missing data will be conducted and outlined in the SAP. Handling of other missing data will be provided in the SAP.

13.11 Subject Disposition

Enrollment and discontinuations from the study and from study drug will be summarized by treatment group, including the reasons for discontinuation from the study and from study drug. Subjects who prematurely discontinue the study or study drug due to COVID-19 will be summarized.

13.12 Deviation Reporting

Protocol deviations will be summarized by treatment group. Protocol deviations are defined as any variation from the protocol, including enrollment of a subject who did not meet all inclusion and exclusion criteria and failure to perform the assessments and procedures within the required time frame.

13.13 Impact of the COVID-19 Pandemic on Analyses

The COVID-19 pandemic may impact study participants' ability to return to the clinic possibly resulting in premature discontinuation of study drug, premature discontinuation of the study,

or missed study visits. Additionally, it is possible the COVID-19 pandemic may impact efficacy and safety outcomes. All missed visits due to COVID-19 will be noted as protocol deviations and diagnoses of COVID-19 will be recorded as medical history or adverse events. Analyses to assess the possible impact of COVID-19 on the study results are described above and will be presented in the SAP.

14.0 INVESTIGATOR REQUIREMENTS

14.1 Protocol Adherence

The Investigator must adhere to the protocol as detailed in this document and agree that the Sponsor must approve any change to the protocol before seeking approval from the IRB/IEC. The Investigator will be responsible for enrolling only those subjects who have met the protocol inclusion and exclusion criteria.

14.2 Electronic Case Report Forms

The contract research organization will make the eCRF accessible to authorized personnel over the internet from an Electronic Data Capture (EDC) system used for the recording of study data as specified by this protocol. All eCRFs must be completed by trained study personnel. The Investigator is responsible for ensuring that the eCRF data are entered and completed in a timely manner.

Once all data queries and issues have been resolved for each subject, the Investigator will electronically sign each subject's eCRF to attest to the accuracy of the data.

14.3 Source Document Maintenance

Source documents are defined as documentation related to original observations and activities of a clinical investigation. Source documents may include, but are not limited to, study progress notes, study- or subject-specific e-mail correspondence, computer printouts, laboratory data, and recorded data from automated instruments. All source documents produced in this study will be maintained by the Investigator and made available for inspections by the Sponsor and by regulatory authorities. The original signed ICF for each participating subject shall be filed with records kept by the Investigator, and a copy shall be given to the subject.

14.4 Study Monitoring Requirements

An authorized Sponsor representative will conduct site visits to inspect study data, subjects' medical records, and eCRFs in accordance with ICH guidelines, GCPs, and the foreign regulations and guidelines, as applicable. A monitor will be utilized for monitoring ongoing drug accountability and adherence to protocol procedures.

The Investigator will allow representatives of the Sponsor and regulatory authorities to inspect facilities and records relevant to this study.

14.5 Study Completion

The Sponsor requires the following data and materials before a study can be considered complete or terminated:

- Laboratory findings, clinical data, and all special test results from Screening throughout the study until the Follow-up visit (Day 52-59)
- eCRFs (including data queries) properly completed by appropriate study personnel and signed and dated by the Investigator
- Copies of complete drug accountability records (drug inventory log and an inventory of returned or destroyed clinical material)
- Copies of protocol amendments and IRB/IEC approval and notification, if appropriate
- A summary of the study prepared by the Investigator (an IRB/IEC summary letter is acceptable)

End of Study Conduct, for the purpose of clinical trial registries, end of trial notifications to regulatory authorities and Institutional Review Boards/Ethics Committees, will be the latest date on which the last visit in time of any study participant occurs.

15.0 QUALITY CONTROL AND QUALITY ASSURANCE

Written Standard Operating Procedures (SOPs) will be followed to ensure that the study is conducted, and data are generated, documented (recorded), and reported in compliance with the protocol, GCP, and the applicable regulatory requirements. Quality control will be applied to each stage of data handling. Regular monitoring, as defined in ICH GCP E6(R2), Section 1.38, "The act of overseeing the progress of a clinical trial, and of ensuring that it is conducted, recorded, and reported in accordance with the protocol, SOPs, GCP, and the applicable regulatory requirement(s)", will be conducted throughout the conduct of the study.

As defined in ICH GCP E6(R2), Section 5.18, the purpose of monitoring is to verify that:

- Rights and well-being of the human subjects are protected
- The reported study data are accurate, complete, and verifiable from source documents
- The conduct of the study is in compliance with the currently approved protocol/amendment(s), with GCP, and with the applicable regulatory requirements
- Monitoring is an integral role in the quality control of a clinical trial and is designed to verify the quality of the study

To fulfill the Quality Assurance requirements of GCP, audits will be conducted to assess and assure the reliability and integrity of a study's quality control systems and recognized standards.

As defined in ICH GCP E6(R2), Section 5.19, the purposes of an audit are to:

- Ensure participant safety
- Assure compliance to study protocol procedures, regulatory requirements, and SOPs
- Assure data quality

16.0 PROTECTION OF HUMAN SUBJECTS

This study will be conducted in compliance with the ICH Technical Requirements for Registration of Pharmaceuticals for Human Use E6 GCP: Consolidated Guidelines, the ethical principles of the Declaration of Helsinki, FDA GCP guidelines, and any additional national or IRB/IEC-required procedures.

16.1 Informed Consent

This study will be conducted in compliance with ICH E6 GCP: Consolidated Guidelines pertaining to informed consent. Subjects will give written consent to participate in the study at the first visit, prior to initiation of any study-related procedures, after having been informed about the nature and purpose of the study, participation and termination conditions, risks, and benefits. If a subject is unable to provide written informed consent, the subject's legally acceptable representative (i.e., acceptable to ICH and local law, as applicable) may provide written consent, as approved according to institution-specific guidelines. The ICF must be signed and dated by the subject, or the subject's legally acceptable representative, prior to study participation. A copy of the ICF must be provided to the subject or the subject's legally acceptable representative. If applicable, it will be provided in certified translation for non-English-speaking subjects. Signed ICFs must remain in the subject's study file and be available for verification by Sponsor at any time.

16.2 IRB/IEC Approval

This protocol, the ICF, and all relevant supporting data must be submitted to the IRB/IEC for approval. The protocol, ICF, and any advertisement used to recruit study subjects must be approved by the IRB/IEC. Approval by the IRB/IEC of the protocol and ICF must be obtained before the study may be initiated.

The Investigator is responsible for informing the IRB/IEC of any changes made to the protocol, and to advise them, at least once a year, about the progress of the study. The Investigator is also responsible for notifying the IRB/IEC of any significant AEs that occur during the study.

17.0 DATA HANDLING AND RECORD KEEPING

Training sessions, regular monitoring of Investigators by Sponsor-designated personnel, instruction manuals, data verification, crosschecking, and data audits will be performed to ensure quality of all study data. Investigator meetings will be performed to prepare Investigators and other study personnel for appropriate collection of study data.

The Sponsor will review and validate study data as defined in the monitoring plan.

It will be the responsibility of the Investigator to ensure that the essential documents are available at the Investigator or institutional site. Any or all of these documents may be subject to, and should be available for, monitoring by the Sponsor or inspection by the regulatory authorities as defined in the monitoring plan.

17.1 Direct Access to Source Data/Documentation

The Investigator agrees by his/her participation that the results of this study may be used for submission to national or international registration. If required, these authorities will be provided with the name of the Investigator and his or her address, qualifications, and extent of involvement. It is understood that the Investigator is required to provide Sponsor with all study data, complete reports, and access to all study records.

Data generated by this study must be available for inspection by any regulatory authorities, by Sponsor and by the IRB/IEC as appropriate. At a subject’s request, medical information may be given to his or her personal physician or other appropriate medical personnel responsible for his or her welfare. Medical information obtained from subjects during the course of this study is confidential and disclosure to third parties other than those noted above is prohibited.

17.2 Study Drug Accountability

All supplies of Rezafungin for Injection, oral fluconazole, placebo for Rezafungin for Injection, placebo for caspofungin IV, and placebo for oral step-down therapy required for completion of this study will be provided by the Sponsor. It is the responsibility of the unblinded Pharmacy staff or unblinded study staff to ensure that a current record of drug inventory and drug accountability is maintained. Inventory and accountability records must be readily available for inspection by the unblinded monitor and are open to inspection at any time by applicable regulatory authorities.

17.3 Retention of Records

Essential documents should be retained until at least 2 years after the last approval of a marketing application in an ICH region and until there are no pending or contemplated marketing applications in an ICH region or at least 2 years have elapsed since the formal discontinuation of clinical development of the investigational product and shipment and delivery of the drug for investigational use is discontinued. These documents should be retained for

a longer period, however, if required by the applicable regulatory requirements of specific ICH- and non-ICH countries, or by an agreement with the Sponsor. The Sponsor will inform the Investigator/institution as to when these documents no longer need to be retained.

18.0 FINANCING AND INSURANCE

The financing and insurance for this study are outlined in the Clinical Trial Agreement.

19.0 PUBLICATION POLICY

The data generated in this clinical study are the exclusive property of the Sponsor and are confidential. Authorship on any publication of the results from this study will be based on contributions to study design, enrollment, data analysis, and interpretation of results.

20.0 REFERENCES

Alexander BD, Johnson MD, Pfeiffer CD, Jiménez-Ortigosa C, Catania J, Booker R, Castanheira M, Messer SA, Perlin DS, Pfaller MA. Increasing echinocandin resistance in *Candida glabrata*: clinical failure correlates with presence of *FKS* mutations and elevated minimum inhibitory concentrations. *Clin Infect Dis*. 2013 Jun;56(12):1724-32. Erratum in: *Clin Infect Dis*. 2014 Mar;58(5):754.

Alastruey-Izquierdo A, Melhem MS, Bonfietti LX, Rodriguez-Tudela JL. Susceptibility test for fungi: clinical and laboratorial correlations in medical mycology. *Rev Inst Med Trop Sao Paulo*. 2015 Sep;57 Suppl 19:57-64.

Andes DR, Safdar N, Baddley JW, Playford G, Reboli AC, Rex JH, Sobel JD, Pappas PG, Kullberg BJ; Mycoses Study Group. Impact of treatment strategy on outcomes in patients with candidemia and other forms of invasive candidiasis: a patient-level quantitative review of randomized trials. *Clin Infect Dis*. 2012 Apr;54(8):1110-22.

Arendrup MC, Dzajic E, Jensen RH, Johansen HK, Kjaeldgaard P, Knudsen JD, Kristensen L, Leitz C, Lemming LE, Nielsen L, Olesen B, Rosenvinge FS, Røder BL, Schønheyder HC. Epidemiological changes with potential implication for antifungal prescription recommendations for fungaemia: data from a nationwide fungaemia surveillance programme. *Clin Microbiol Infect*. 2013 Aug;19(8):E343-53. Erratum in: *Clin Microbiol Infect*. 2013 Aug;19(8):E376.

Arendrup MC, Sulim S, Holm A, Nielsen L, Nielsen SD, Knudsen JD, Drenck NE, Christensen JJ, Johansen HK. Diagnostic issues, clinical characteristics, and outcomes for patients with fungemia. *J Clin Microbiol*. 2011 Sep;49(9):3300-8.

Bader JC, Lakota EA, Bhavnani SM, Ambrose PG. Emerging *Candida glabrata* resistance and echinocandin dosing: A call to arms. IDWeek 2016, New Orleans LA, abstract #1973.

Balkovec JM, Hughes DL, Masurekar PS, Sable CA, Schwartz RE, Singh SB. Discovery and development of first in class antifungal caspofungin (CANCIDAS®)—a case study. *Nat Prod Rep.* 2014;31(1):15–34.

Berkow EL, Lockhart SR. Activity of CD101, a long-acting echinocandin, against clinical isolates of *Candida auris*. *Diagn Microbiol Infect Dis*. 2018;90(3):196–197.

Cancidas® (caspofungin acetate) [Prescribing Information]. Whitehouse Station, NJ: Merck & Co., Inc.; 2019 February. Available at: https://www.merck.com/product/usa/pi_circulars/c/cancidas/cancidas_pi.pdf. Accessed 19Sep2020.

Cancidas® (caspofungin acetate) powder for concentrate for solution for infusion [Summary of Product Characteristics]. Haarlem, Netherlands: Merck Sharp & Dohme B.V.; 2020 August. Avaialble at https://www.medicines.org.uk/emc/medicine/12843#gref. Accessed 13Oct2020.

Caspofungin acetate [Prescribing Information]. Hyderabad, India: Glanda Pharma Limited; 2019 March. Available at https://dailymed.nlm.nih.gov/dailymed/lookup.cfm?setid=29da357e-e7fc-46af-bf20-806a1e4b5391. Accessed 13Oct2020.

CDC 2013. Antibiotic resistance threats in the United States, 2013. http://www.cdc.gov/drugresistance/threat-report-2013/

CDC 2016. Clinical Alert to United States (US) Healthcare Facilities: Global emergence of invasive infections caused by the multidrug-resistant yeast *Candida auris* 2016. https://www.cdc.gov/fungal/diseases/candidiasis/candida-auris-alert.html. Accessed April 25, 2018.

Cornely OA, Bassetti M, Calandra T, Garbino J, Kullberg BJ, Lortholary O, Meersseman W, Akova M, Arendrup MC, Arikan-Akdagli S, Bille J, Castagnola E, Cuenca-Estrella M, Donnelly JP, Groll AH, Herbrecht R, Hope WW, Jensen HE, Lass-Flörl C, Petrikkos G, Richardson MD, Roilides E, Verweij PE, Viscoli C, Ullmann AJ; ESCMID Fungal Infection Study Group. ESCMID* guideline for the diagnosis and management of *Candida* diseases 2012: non-neutropenic adult patients. *Clin Microbiol Infect*. 2012 Dec;18 Suppl 7:19-37.

Cousin L, Berre ML, Launay-Vacher V, Izzedine H, Deray G. Dosing guidelines for fluconazole in patients with renal failure. Nephrol Dial Transplant. 2003 Nov;18(11):2227-31.

Diflucan® (fluconazole) tablets [Prescribing Information]. New York, NY; Pfizer Roerig; 2020 March. Available at http://labeling.pfizer.com/ShowLabeling.aspx?id=575. Accessed 09Aug2020.

Ecalta® (anidulafungin) 100 mg powder for concentrate for solution for infusion [Summary of Product Characteristics]. Brussels, Belgium; Pfizer Europe MA EEIG; 2020 June. Available at https://www.medicines.org.uk/emc/product/454/smpc#gref. Accessed 13Oct2020.

Eraxis™ (anidulafungin) [Prescribing Information]. New York, NY; Pfizer Roerig; 2020 May. Available at http://labeling.pfizer.com/ShowLabeling.aspx?format=PDF&id=566. Accessed 19Sep2020.

European Medicines Agency, Eudravigilance System Overview: Important Medical Event list. Available at https://www.ema.europa.eu/en/human-regulatory/research-development/pharmacovigilance/eudravigilance/eudravigilance-system-overview. Accessed 24Apr2019.

Executive Office of the President, President's Council of Advisors on Science and Technology. Report to the President on Combating Antibiotic Resistance. September 2014.

Gudlaugsson O, Gillespie S, Lee K et al. Attributable mortality of nosocomial candidemia, revisited. *Clin Infect Dis*. 2003; 37:1172-7.

Kett DH, Azoulay E, Echeverria PM, Vincent JL; Extended Prevalence of Infection in ICU Study (EPIC II) Group of Investigators. *Candida* bloodstream infections in intensive care units: analysis of the extended prevalence of infection in intensive care unit study. Crit Care Med. 2011 Apr;39(4):665-70.

Knaus WA, Draper EA, Wagner DP, Zimmerman JE. APACHE II: a severity of disease classification system. *Crit Care Med*. 1985 Oct;13(10):818-29.

Lakota EA, Voon O, Flanagan S, Rubino CM. Population Pharmacokinetic Analyses for Rezafungin (CD101) Efficacy Using Phase 1 Data: Rezafungin (CD101) Population Pharmacokinetic Model. *Antimicrob. Agents Chemother*. Posted Online 19 March 2018. http://aac.asm.org/content/early/2018/03/14/AAC.02603-17.long
Accessed April 26, 2018.

Magill SS, Edwards JR, Bamberg W, Beldavs ZG, Dumyati G, Kainer MA, Lynfield R, Maloney M, McAllister-Hollod L, Nadle J, Ray SM, Thompson DL, Wilson LE, Fridkin SK; Emerging Infections Program Healthcare-Associated Infections and Antimicrobial Use Prevalence Survey Team. Multistate point-prevalence survey of health care-associated infections. *N Engl J Med*. 2014 Mar 27;370(13):1198-208.

Mycamine® (micafungin sodium) [Prescribing Information]. Northbrook, IL: Astellas Pharma US, Inc.; 2020 July. Available at https://www.us.astellas.com/docs/mycamine.pdf. Accessed 19Sep2020.

Mycamine (micafungin sodium) powder for concentrate for solution for infusion [Summary of Product Characteristics]. Leiden, Netherlands: Astellas Pharma Europe B.V.; 2020 January. Available at https://www.medicines.org.uk/emc/product/6315/smpc#gref. Accessed 13Oct2020.

Ostrosky-Zeichner L. *Candida glabrata* and FKS mutations: witnessing the emergence of the true multidrug-resistant *Candida*. *Clin Infect Dis*. 2013; 56: 1733–4.

Pappas PG, Kauffman CA, Andes DR, Clancy CJ, Marr KA, Ostrosky-Zeichner L, Reboli AC, Schuster MG, Vazquez JA, Walsh TJ, Zaoutis TE, Sobel JD. Clinical Practice Guideline for the Management of Candidiasis: 2016 Update by the Infectious Diseases Society of America. *Clin Infect Dis*. 2016 Feb 15;62(4):409-17.

Pfaller MA. Antifungal drug resistance: mechanisms, epidemiology, and consequences for treatment. *Am J Med*. 2012; 125: S3–13.

Pfaller MA, Carvalhaes C, Messer SA, Rhomberg PR, Castanheira M. Activity of a long-acting echinocandin, rezafungin, and comparator antifungal agents tested against contemporary invasive fungal isolates (SENTRY Program, 2016 to 2018). *Antimicrob Agents Chemother*. 2020;64(4): e00099-20.

Subramony SH, May W, Lynch D, et al. Measuring Friedreich ataxia: Interrater reliability of a neurologic rating scale. *Neurology*. 2005; 64:1261–1262.

Ullmann AJ, Akova M, Herbrecht R, Viscoli C, Arendrup MC, Arikan-Akdagli S, Bassetti M, Bille J, Calandra T, Castagnola E, Cornely OA, Donnelly JP, Garbino J, Groll AH, Hope WW, Jensen HE, Kullberg BJ, Lass-Flörl C, Lortholary O, Meersseman W, Petrikkos G, Richardson MD, Roilides E, Verweij PE, Cuenca-Estrella M; ESCMID Fungal Infection Study Group. ESCMID guideline for the diagnosis and management of *Candida* diseases 2012: adults with haematological malignancies and after haematopoietic stem cell transplantation (HCT). *Clin Microbiol Infect*. 2012 Dec;18 Suppl 7:53-67.

Teasdale G, Jennett B. Assessment of coma and impaired consciousness. A practical scale. *Lancet*. 1974 Jul 13;2(7872):81-4.

The White House. National Strategy for Combating Antibiotic Resistant Bacteria. September 2014.

Wisplinghoff H, Bischoff T, Tallent SM, Seifert H, Wenzel RP, Edmond MB. Nosocomial bloodstream infections in US hospitals: analysis of 24,179 cases from a prospective nationwide surveillance study. *Clin Infect Dis*. 2004; 39:309–17.

Zhao Y, Perez WB, Jimenez-Ortigosa C, Hough G, Locke JB, Ong V, Bartizal K, Perlin DS. CD101: a novel long-acting echinocandin. Cell Microbiol. 2016. 18:1308-1316.

Zhao Y, Prideaux B, Nagasaki Y, Lee MH, Chen PY, Blanc L, Ho H, Clancy CJ, Nguyen MH, Dartois V, Perlin DS. Unraveling Drug Penetration of Echinocandin Antifungals at the Site of Infection in an Intra-abdominal Abscess Model. *Antimicrob Agents Chemother*. 2017 Sep 22;61(10). pii: e01009-17.

21.0 APPENDICES

APPENDIX 1: CLINICAL AND LABORATORY STANDARDS INSTITUTE (CLSI) AND EUROPEAN COMMITTEE ON ANTIMICROBIAL SUSCEPTIBILITY TESTING (EUCAST) BREAKPOINTS FOR *CANDIDA* SPP.

The following table provides susceptibility breakpoints for both Clinical and Laboratory Standards Institute (CLSI) and European Committee on Antimicrobial Susceptibility Testing (EUCAST) and should be used as guidance in determining susceptibility of different *Candida* spp. to potential empiric antifungal drugs. Not all spp. or antifungal drugs are referenced here, and local determination of susceptibility and resistance may be used when appropriate.

		MIC Breakpoints (mg/L)									
Antifungal Agent	Standard	*C. albicans*		*C. glabrata*		*C. krusei*		*C. parapsilosis*		*C. tropicalis*	
		S ≤	R >	S ≤	R >	S ≤	R >	S ≤	R >	S ≤	R >
Amphotericin B	EUCAST	1	1	1	1	1	1	1	1	1	1
	CLSI	-	-	-	-	-	-	-	-	-	-
Anidulafungin	EUCAST	0.03	0.03	0.06	0.06	0.06	0.06	0.002	4	0.06	0.06
	CLSI	0.25	0.5	0.12	0.25	0.25	0.5	2	4	0.25	0.5
Caspofungin	EUCAST										
	CLSI	0.25	0.5	0.125	0.25	0.25	0.5	2	4	0.25	0.5
Fluconazole	EUCAST	2	4	0.002	32	-	-	2	4	2	4
	CLSI	2	4	32 (SDD)	32	-	-	2	4	2	4
Itraconazole	EUCAST	0.06	0.06	IE	IE	IE	IE	0.12	0.12	0.12	0.12
	CLSI	0.125	0.5	0.125	0.5	0.125	0.5	0.125	0.5	0.125	0.5
Micafungin	EUCAST	0.016	0.016	0.03	0.03	IE	IE	0.002	2	IE	IE
	CLSI	0.25	0.5	0.06	0.12	0.25	0.5	2	4	0.25	0.5
Posaconazole	EUCAST	0.06	0.06	IE	IE	IE	IE	0.06	0.06	0.06	0.06
	CLSI	-	-	-	-	-	-	-	-	-	-

MIC Breakpoints (mg/L)											
Antifungal Agent	Standard	*C. albicans*		*C. glabrata*		*C. krusei*		*C. parapsilosis*		*C. tropicalis*	
		S ≤	R >	S ≤	R >	S ≤	R >	S ≤	R >	S ≤	R >
Voriconazole	EUCAST	0.125	0.125	IE	IE	IE	IE	0.125	0.125	0.125	0.125
	CLSI	0.125	0.5			0.5	1	0.125	0.5	0.125	0.5

Abbreviations: CLSI = Clinical and Laboratory Standards Institute; EUCAST = European Committee on Antimicrobial Susceptibility Testing; IE = insufficient evidence; R = resistant; S = susceptible; SDD = susceptible dose dependent.
Source: Alastruey-Izquierdo 2015.

APPENDIX 2: CREATININE CLEARANCE

In the event the local laboratory does not calculate creatinine clearance based on the most recent serum creatinine value, estimate the patient's creatinine clearance using the serum creatinine value provided by the local laboratory, actual body weight, and the appropriate Cockcroft-Gault formula. If necessary, convert serum creatinine values from µmol/L to mg/dL by dividing by 88.4. For example, 100 µmol/L divided by 88.4 equals 1.131 mg/dL:

Males:

$$\text{Creatinine clearance} = \frac{(140 - [\mathit{insert\ age\ in\ years}]) \times [\mathit{insert\ weight\ in\ kg}]}{72 \times [\mathit{insert\ serum\ creatinine\ in\ mg/dL}]}$$

Females:

$$\text{Creatinine clearance} = 0.85 \times \frac{(140 - [\mathit{insert\ age\ in\ years}]) \times [\mathit{insert\ weight\ in\ kg}]}{72 \times [\mathit{insert\ serum\ creatinine\ in\ mg/dL}]}$$

APPENDIX 3: LABORATORY TESTS

Hematology	Serum Chemistry
hemoglobin	blood urea nitrogen [c]
hematocrit	bilirubin (total and direct)
total and differential leukocyte count	alkaline phosphatase
red blood cell count	aspartate aminotransferase
platelet count	alanine aminotransferase
**Urinalysis**	albumin
pH	sodium
specific gravity	potassium
protein [a]	chloride
glucose	glucose
ketones	creatinine [b]
bilirubin	total protein
blood [a]	calcium (total)
nitrite [a]	bicarbonate [d]
urobilinogen	magnesium
leukocyte esterase [a]	
**Coagulation Tests**	**Additional Tests**
PT/INR	serum pregnancy (for females only)
	blood culture within 12 hours of randomization (required for candidemia subjects)

Abbreviations: PT/INR = Prothrombin Time or International Normalized Ratio

a. If urinalysis is positive for protein, blood, nitrite and/or leukocyte esterase, a microscopic examination (for red blood cells, white blood cells, bacteria, casts, and epithelial cells) will be performed.

b. At Screening, creatinine clearance will be calculated using the Cockcroft-Gault formula (Appendix 2).

c. Study sites that do not perform BUN testing may use a Urea test result, instead, for entry into EDC. If the Urea test result is reported in mg/dL, site staff will need to convert the Urea test result(s) for their subject(s) to a corresponding BUN value by using the following conversion factor:

- Urea [mg/dL] x 0.467 = BUN [mg/dL]

If the Urea test result is reported in mmol/L, site staff will not need to convert this, as Urea [mmol/L] = BUN [mmol/L]. In this circumstance, the Urea (mmol/L) value can be entered directly into the eCRF as BUN (mmol/L). File a copy of this memo in the On-Site Study File.

d. Total CO_2 may be used instead of bicarbonate as they are interchangeable and no conversion is required.

APPENDIX 4: CHILD-PUGH SCORE

Calculate the Child-Pugh score only if the subject has a history of chronic cirrhosis.

The Child-Pugh score employs 5 clinical measures of liver disease. Each measure is scored 1 to 3, with 1 indicating the least severe and 3 indicating the most severe score. The sum of the 5 measures is the total Child-Pugh point score.

**Parameter**	**Points**		
	**1**	**2**	**3**
Ascites	None	Medically controlled	Poorly controlled
Encephalopathy [a]	None	Medically controlled	Poorly controlled
Total bilirubin (mg/dL)	<2 mg/dL<br>(<34.2 µmol/L)	2–3 mg/dL<br>(34.2–51.3 µmol/L)	>3 mg/dL<br>(>51.3 µmol/L)
Albumin (g/dL)	>3.5 g/dL<br>(>35 g/L)	2.8–3.5 g/dL<br>(28–35 g/L)	<2.8 g/dL<br>(<28 g/L)
International Normalized Ratio<br>**OR**<br>PT prolongation in seconds	<1.7<br><br><4	1.7–2.3<br><br>4–6	>2.3<br><br>>6

Abbreviations: PT = prothrombin time.

a. If sedated, use last known encephalopathy status before sedation.

APPENDIX 5: MODIFIED ACUTE PHYSIOLOGY AND CHRONIC HEALTH EVALUATION (APACHE) II SCORE

**Point Value**	**4**	**3**	**2**	**1**	**0**	**1**	**2**	**3**	**4**	**Total**
Glasgow Coma Score	Score = 15 minus actual Glasgow Coma Score									
Temperature (ºC)	≥41	39–40.9		38.5–38.9	36–38.4	34–35.9	32–33.9	30–31.9	≤29.9	
Mean Arterial Pressure (mmHg) [a]	≥160	130–159	110–129		70–109		50–69		≤49	
Heart Rate (bpm)	≥180	140–179	110–139		70–109		55–69	40–54	≤39	
Respiration Rate	≥50	35–49		25–34	12–24	10–11	6–9		≤5	
Alveolar-Arterial Oxygen Gradient (if FiO_2 ≥50%)<br>OR<br>PaO_2 (mmHg)<br>OR<br>Oxygen Saturation, %	≥500	350 to 499	200 to 349		<200<br>>70<br>≥92	 61–70<br>88–91		 55–60<br>85–87	 <55<br><85	
Arterial pH<br>OR<br>Serum HCO_3 (mEq/L) – (venous- if ABG not performed)	≥7.7<br>≥52	7.6–7.69<br>41–51.9		7.5–7.59<br>32–40.9	7.33–7.49<br>22–31.9		7.25–7.32<br>18–21.9	7.15–7.24<br>15–17.9	<7.15<br><15	
Serum Na^+ (mMol/L)	≥180	160–179	155–159	150–154	130–149		120–129	111–119	≤110	
Serum K^+ (mMol/L)	≥7.0	6.0–6.9		5.5–5.9	3.5–5.4	3.0–3.4	2.5–2.9		<2.5	
Serum creatinine (mg/dL)	≥3.5	2.0–3.4	1.5–1.9		0.6–1.4		<0.6			
Acute renal failure [b]	×2 creatinine point score if patient has acute renal failure									
Hematocrit, %	≥60		50–59.9	46–49.9	30–45.9		20–29.9		<20	
WBC, (10^9 cells/L or mm^3)	≥40		20–39.9	15–19.9	3–14.9		1–2.9		<1	

**Point Value**	**4**	**3**	**2**	**1**	**0**	**1**	**2**	**3**	**4**	**Total**
Severe organ failure (liver, heart, or lung) or immunocompromised [c]	Add 5 points if patient is medical (no surgery) or postoperative for emergency surgery<br>Add 2 points if patient is postelective surgery									
Age (years) points	≤44 = 0; 45–54 = 2; 55–64 = 3; 65–74 = 5; ≥75 = 6									
	**Sum of all rows**									

Source: Modified from Knaus 1985.

a. Mean arterial pressure = (systolic blood pressure + [2 × diastolic blood pressure])/3

b. Acute Renal Failure = a 0.5 mg/dL increase in serum creatinine if the baseline serum creatinine was ≤1.9 mg/dL, a 1.0 mg/dL increase in serum creatinine if the baseline serum creatinine was 2.0 to 4.9 mg/dL, and a 1.5 mg/dL increase in serum creatinine if the baseline serum creatinine was ≥5.0 mg/dL.

c. Organ insufficiency or immunocompromised state must have been evident **prior** to this hospital admission and conform to the following criteria:

**Liver**	Biopsy proven cirrhosis and documented portal hypertension; episodes of past upper gastrointestinal bleeding attributed to portal hypertension; or prior episodes of hepatic failure/encephalopathy/coma.
**Cardiovascular**	New York Heart Association Class IV.
**Respiratory**	Chronic restrictive, obstructive, or vascular disease resulting in severe exercise restriction (i.e., unable to climb stairs or perform household duties; or documented chronic hypoxia, hypercapnia, secondary polycythemia, severe pulmonary hypertension (>40 mmHg), or respirator dependency.
**Renal**	Receiving chronic dialysis.
**Immunocompromised**	The patient has received therapy that suppresses resistance to infection (e.g., immunosuppression, chemotherapy, radiation, long term or recent high dose steroids, or has a disease that is sufficiently advanced to suppress resistance to infection, e.g., leukemia, lymphoma, AIDS).

APPENDIX 6: GLASGOW COMA SCORE

Sedating and paralytic agent use may confound the assessment of the Glasgow Coma Score. If sedation is reduced and patients awakened daily to perform neurologic assessments, assess the score during the period when the patient is not sedated. If daily awakenings are not performed, please record the score as if the patient did not have sedating or paralytic agents preventing response.

**Neurologic Response**		**Score**
Best Eye Response (E)	Spontaneous-open with blinking at baseline	4
	Opens to verbal command, speech or shout	3
	Opens to pain (not applied to face)	2
	None	1
Best Verbal Response (V)	Oriented	5
	Confused conversation, but can answer questions	4
	Inappropriate responses, words discernible	3
	Incomprehensible speech	2
	None	1
Best Motor Response (M)	Obeys commands for movement	6
	Purposeful movement to painful stimulus	5
	Withdraws from pain	4
	Abnormal (spastic) flexion, decorticate posture	3
	Extensor (rigid) response, decerebrate posture	2
	None	1
**Total Score**		

Source: Teasdale 1974.

APPENDIX 7: POTENTIAL DRUG INTERACTIONS, WARNINGS, AND PRECAUTIONS

Appendix 7A: Caspofungin Potential Drug Interactions, Warnings, and Precautions

Caspofungin is contraindicated in subjects with hypersensitivity to the active substance or any of the excipients. Hypersensitivity includes but is not limited to hereditary sugar disorders (e.g., fructose intolerance or sucrose-isomaltase deficiency), anaphylaxis and exfoliative skin disorders (e.g., Stevens-Johnson syndrome or toxic epidermal necrolysis), which may be severe.

Caspofungin is not a substrate for P-glycoprotein and is a poor substrate for cytochrome P450 enzymes. However, caspofungin has been shown to interact with other medicinal products in pharmacological and clinical studies.

Concomitant administration of the following medicinal products with caspofungin requires precaution and dose adjustments may be required:

Cyclosporine	May lead to an increase in caspofungin AUC and may increase the risk of hepatic toxicity
Carbamazepine	Limited data from population PK studies indicate that concomitant use of carbamazepine may result in a decrease of caspofungin AUC. When co-administering inducers of metabolic enzymes, an increase in the daily dose of caspofungin to 70 mg, following the 70 mg loading dose, should be considered in adult patients.
Dexamethasone	Limited data from population PK studies indicate that concomitant use of dexamethasone may result in a decrease of caspofungin AUC. When co-administering inducers of metabolic enzymes, an increase in the daily dose of caspofungin to 70 mg, following the 70 mg loading dose, should be considered in adult patients.
Efavirenz	Limited data from population PK studies indicate that concomitant use of efavirenz may result in a decrease of caspofungin AUC. When co-administering inducers of metabolic enzymes, an increase in the daily dose of caspofungin to 70 mg, following the 70 mg loading dose, should be considered in adult patients.
Nevirapine	Limited data from population PK studies indicate that concomitant use of nevirapine may result in a decrease of caspofungin AUC. When co-administering inducers of metabolic enzymes, an increase in the daily dose of caspofungin to 70 mg, following the 70 mg loading dose, should be considered in adult patients.
Phenytoin	Limited data from population PK studies indicate that concomitant use of phenytoin may result in a decrease of caspofungin AUC. When co-administering inducers of metabolic enzymes, an increase in the daily dose of caspofungin to 70 mg, following the 70 mg loading dose, should be considered in adult patients.

Rifampin (Rifampicin)	Rifampicin caused a 60 % increase in AUC and 170 % increase in trough concentration of caspofungin on the first day of co-administration when both medicinal products were initiated together in healthy adult volunteers. Caspofungin trough levels gradually decreased upon repeated administration. After two weeks' administration rifampicin had limited effect on AUC, but trough levels were 30% lower than in adult subjects who received caspofungin alone. The mechanism of interaction could possibly be due to an initial inhibition and subsequent induction of transport proteins.
Tacrolimus	Caspofungin reduced the trough concentration of tacrolimus by 26% in healthy adult volunteers. For patients receiving both therapies, standard monitoring of tacrolimus blood concentrations and appropriate tacrolimus dosage adjustments are mandatory.

Appendix 7B: Fluconazole Potential Drug Interactions, Warnings, and Precautions

**List of drugs that can cause prolonged QT interval when co-administered with fluconazole:**
terfenadine<br>cisapride<br>astemizole<br>erythromycin<br>pimozide<br>quinidine<br>halofantrine

Concomitant use of the following supplements, such as St. John's Wort, grapefruit, or Seville oranges should be avoided. Concomitant administration of the following medicinal products with fluconazole requires precaution and dose adjustments may be required:

Alfentanil	A reduction in clearance and distribution volume as well as prolongation of half-life of alfentanil following concomitant treatment with fluconazole was observed. Dosage adjustment of alfentanil may be necessary.
Amitriptyline, nortriptyline	Fluconazole increases the effect of amitriptyline and nortriptyline. 5-nortriptyline and/or S-amitriptyline may be measured at initiation of the combination therapy and after one week. Dose of amitriptyline/ nortriptyline should be adjusted, if necessary.
Azithromycin	There was no significant pharmacokinetic interaction between fluconazole and azithromycin in a study of 18 healthy volunteers.
Calcium channel blockers: nifedipine, isradipine, amlodipine, verapamil, and felodipine	Certain calcium channel antagonists are metabolized by CYP3A4. Fluconazole has the potential to increase the systemic exposure of the calcium channel antagonists. Frequent monitoring for adverse events is recommended.

Carbamazepine	Fluconazole inhibits the metabolism of carbamazepine and an increase in serum carbamazepine of 30% has been observed. There is a risk of developing carbamazepine toxicity. Dose adjustment of carbamazepine may be necessary depending on concentration measurements/effect.
Celecoxib	During concomitant treatment with fluconazole and celecoxib, the celecoxib C_{max} and AUC increased by 68% and 134%, respectively. Half of the celecoxib dose may be necessary when combined with fluconazole.
Coumarin-type anticoagulants (e.g., warfarin)	Prothrombin time may be increased in patients receiving concomitant fluconazole and coumarin-type anticoagulants. Bleeding events (bruising, epistaxis, gastrointestinal bleeding, hematuria, and melena) have been reported in association with increases in prothrombin time in patients receiving fluconazole concurrently with warfarin. Careful monitoring of prothrombin time in patients receiving fluconazole and coumarin-type anticoagulants is recommended. Dose adjustment of the anticoagulant may be necessary.
Cyclophosphamide	Combination therapy with cyclophosphamide and fluconazole results in an increase in serum bilirubin and serum creatinine.
Fentanyl	One fatal case of fentanyl intoxication due to possible fentanyl fluconazole interaction was reported in the literature. Furthermore, it was shown in healthy volunteers that fluconazole delayed the elimination of fentanyl significantly. Elevated fentanyl concentration may lead to respiratory depression. Patients should be monitored closely for the potential risk of respiratory depression. Dosage adjustment of fentanyl may be necessary.
HMG-CoA reductase inhibitors: simvastatin, atorvastatin, fluvastatin	The risk of myopathy and rhabdomyolysis increases when fluconazole is co-administered with HMG-CoA reductase inhibitors metabolized through CYP3A4, such as atorvastatin and simvastatin, or through CYP2C9, such as fluvastatin.<br>If concomitant therapy is necessary, the patient should be observed for symptoms of myopathy and rhabdomyolysis and creatine kinase should be monitored. HMG-CoA reductase inhibitors should be discontinued if a marked increase in creatine kinase is observed or myopathy/rhabdomyolysis is diagnosed or suspected.
Ibrutinib	Moderate inhibitors of CYP3A4 such as fluconazole increase plasma ibrutinib concentrations and may increase risk of toxicity. If the combination cannot be avoided, reduce the dose of ibrutinib as instructed in ibrutinib prescribing information and provide close clinical monitoring.
Immunosuppresors: cyclosporine, everolimus, sirolimus, tacrolimus	Fluconazole significantly increases the concentration and AUC of cyclosporine. This combination may be used by reducing the dose of cyclosporine depending on cyclosporine concentration.<br>Although not studied in vivo or in vitro, fluconazole may increase serum concentrations of everolimus through inhibition of CYP3A4.<br>Fluconazole increases plasma concentrations of sirolimus presumably by inhibiting the metabolism of sirolimus via CYP3A4 and P-glycoprotein. This combination may be used with

	a dose adjustment of sirolimus depending on the effect/concentration measurements.<br>Fluconazole may increase the serum concentrations of orally administered tacrolimus up to 5 times due to inhibition of tacrolimus metabolism through CYP3A4 in the intestines. No significant pharmacokinetic changes have been observed when tacrolimus is given intravenously. Increased tacrolimus levels have been associated with nephrotoxicity. Dose of orally administered tacrolimus should be decreased depending on tacrolimus concentration.
Losartan	Fluconazole inhibits the metabolism of losartan to its active metabolite (E-31 74) which is responsible for most of the angiotensin Il-receptor antagonism which occurs during treatment with losartan. Patients should have their blood pressure monitored continuously.
Methadone	Fluconazole may enhance the serum concentration of methadone. Dose adjustment of methadone may be necessary.
NSAIDs	The C_{max} and AUC of flurbiprofen was increased by 23% and 81%, respectively, when co-administered with fluconazole compared to administration of flurbiprofen alone. Similarly, the C_{max} and AUC of the pharmacologically active isomer [S-(+)-ibuprofen] was increased by 15% and 82%, respectively, when fluconazole was co-administered with racemic ibuprofen (400 mg) compared to administration of racemic ibuprofen alone. Although not specifically studied, fluconazole has the potential to increase the systemic exposure of other NSAIDs that are metabolized by CYP2C9 (e.g., naproxen, lornoxicam, meloxicam, diclofenac). Frequent monitoring for adverse events and toxicity related to NSAIDs is recommended. Adjustment of dose of NSAIDs may be needed.
Olaparib	Moderate inhibitors of CYP3A4 such as fluconazole increase olaparib plasma concentrations; concomitant use is not recommended. If the combination cannot be avoided, reduce the dose of olaparib.
Oral contraceptives	Two pharmacokinetic studies with a combined oral contraceptive have been performed using multiple doses of fluconazole. There were no relevant effects on hormone level in the 50 mg fluconazole study, while at 200 mg daily, the AUCs of ethinyl estradiol and levonorgestrel were increased 40% and 24%, respectively. Thus, multiple dose use of fluconazole at these doses is unlikely to have an effect on the efficacy of the combined oral contraceptive.
Oral hypoglycemics: tolbutamide, glyburide, and glipizide (or other sulfonylureas)	Fluconazole has been shown to prolong the serum half-life of concomitantly administered oral sulfonylureas (e.g., chlorpropamide, glibenclamide, glipizide, tolbutamide) in healthy volunteers. Frequent monitoring of blood glucose and appropriate reduction of sulfonylurea dose is recommended during co-administration.

Phenytoin	Fluconazole inhibits the hepatic metabolism of phenytoin. Concomitant repeated administration of 200 mg fluconazole and 250 mg phenytoin intravenously, caused an increase of the phenytoin AUC_{24} by 75% and C_{min} by 128%. With co-administration, serum phenytoin concentration levels should be monitored in order to avoid phenytoin toxicity.
Prednisone	A case report in the literature reported that a liver-transplanted patient treated with prednisone developed acute adrenal cortex insufficiency when a three month therapy with fluconazole was discontinued. The discontinuation of fluconazole presumably caused an enhanced CYP3A4 activity which led to increased metabolism of prednisone. Patients on long-term treatment with fluconazole and prednisone should be carefully monitored for adrenal cortex insufficiency when fluconazole is discontinued.
Rifabutin	There have been reports that an interaction exists when fluconazole is administered concomitantly with rifabutin, leading to increased serum levels of rifabutin up to 80%. There have been reports of uveitis in patients to whom fluconazole and rifabutin were co-administered. Patients receiving rifabutin and fluconazole concomitantly should be monitored.
Rifampin/rifampicin	Concomitant administration of fluconazole and rifampicin resulted in a 25% decrease in the AUC and a 20% shorter half-life of fluconazole. Concomitant use should be avoided.
Short acting benzodiazepines (e.g., midazolam, triazolam)	Following oral administration of midazolam, fluconazole resulted in substantial increases in midazolam concentrations and psychomotor effects. Concomitant intake of fluconazole 200 mg and midazolam 7.5 mg orally increased the midazolam AUC and half-life 3.7-fold and 2.2-fold, respectively. Fluconazole 200 mg daily given concurrently with triazolam 0.25 mg orally increased the triazolam AUC and half-life 4.4-fold and 2.3-fold, respectively. Potentiated and prolonged effects of triazolam have been observed at concomitant treatment with fluconazole. If concomitant benzodiazepine therapy is necessary in patients being treated with fluconazole, consideration should be given to decreasing the benzodiazepine dose, and the patients should be appropriately monitored.
Theophylline	In a placebo-controlled interaction study, the administration of fluconazole 200 mg for 14 days resulted in an 18% decrease in the mean plasma clearance rate of theophylline. Patients who are receiving high dose theophylline or who are otherwise at increased risk for theophylline toxicity should be observed for signs of theophylline toxicity while receiving fluconazole. Therapy should be modified if signs of toxicity develop.
Tofacitinib	Systemic exposure to tofacitinib is increased when tofacitinib is co-administered with fluconazole. Reduce the dose of tofacitinib when given concomitantly with fluconazole.

Tolvaptan	Exposure to tolvaptan is significantly increased (200% in AUC; 80% in C_{max}) when tolvaptan, a CYP3A4 substrate, is co-administered with fluconazole, a moderate CYP3A4 inhibitor, with risk of significant increase in adverse effects particularly significant diuresis, dehydration, and acute renal failure. In case of concomitant use, the tolvaptan dose should be reduced and the patient managed cautiously.
Vitamin A	Based on a literature case report in one patient receiving combination therapy with all-trans-retinoid acid (an acid form of vitamin A) and fluconazole, CNS related undesirable effects have developed in the form of pseudotumour cerebri, which disappeared after discontinuation of fluconazole treatment. This combination may be used but the incidence of CNS-related undesirable effects should be borne in mind.
Voriconazole	Monitoring for adverse events and toxicity related to voriconazole is recommended, especially if voriconazole is started within 24 hours after the last dose of fluconazole.

APPENDIX 8: PHARMACOKINETIC SAMPLE PROCESSING AND SHIPMENT

Plasma PK sample collection and processing procedure

1. Draw blood into a 4 mL lavender top potassium ethylenediaminetetraacetic acid (K_2EDTA) Vacutainer tube. Label the tube.
2. Mix the blood with the anticoagulant by gently inverting the tube 8 to 10 times, and then immediately place the tube on wet ice. It is preferable to leave the sample on wet ice for 5 to 10 minutes before centrifuging.
3. Centrifuge the blood samples for approximately 10 minutes at approximately 1006g in a centrifuge within approximately 60 minutes after blood sample collection.
4. Immediately following centrifugation, gently remove the plasma from the packed cells and aliquot into 2 transfer vials. Into the first, aliquot ≥0.5 mL of plasma, and, into the second, aliquot the rest.
5. Replace the caps on the transfer vials and freeze the samples immediately at approximately -20°C or colder.
6. Note: No more than 60 minutes should elapse between blood collection and freezing the plasma samples.
7. Keep the samples frozen at approximately -20°C or colder until shipment.
8. Ship the primary sample on dry ice; retain the frozen backup sample until receipt of instructions regarding its shipment.

NOTE: Wet ice is defined as a mixture of ice and water. Specific details for processing and shipment of PK samples are noted in the Laboratory Manual.

APPENDIX 9: LIST OF PROHIBITED NEUROTOXIC MEDICATIONS

LIST OF PROHIBITED NEUROTOXIC CONCOMITANT MEDICATIONS [a]	
**Definite High Risk**	
vinca alkaloids (Vincristine)	
taxols (paclitaxel, docetaxel, cabazitaxel)	
**Moderate Risk**	
amiodarone (Cordarone)	leflunomide (Arava)
arsenic trioxide (Trisenox)	lenalidomide (Revlimid)
bortezomib (Velcade)	metronidazole/misonidazole (extended use)
brentuximab vedotin (Adcetris)	nitrofurantoin (Macrodantin, Furadantin, Macrobid)
cetuximab (Erbitux)	nitrous oxide
cisplatin & oxaliplatin	nivolumab (Opdivo)
colchicine (extended use)	pembrolizumab (Keytruda)
dapsone [b]	perhexiline (not used in U.S.)
didanosine (ddI, Videx)	pomalidomide (Pomalyst)
dichloroacetate	stavudine (d4T, Zerit)
disulfiram (Antabuse)	suramin
eribulin (Halaven)	thalidomide
gold salts	zalcitabine (ddC, Hivid)
ipilimumab (Yervoy)	
ixabepilone (Ixempra)	

[a] Adapted from the Charcot Marie Tooth Association list of neurotoxic medications.

APPENDIX 10: INVESTIGATOR SIGNATURE

I have read and understand this protocol. I agree to the following:

1) To conduct the trial in compliance with GCP, with applicable regulatory requirement(s), with the protocol agreed to by the Sponsor and given approval/favorable opinion by the IRB/IEC
2) To comply with procedures for data recording and reporting
3) To permit monitoring, auditing, and inspection by the Sponsor, its designated representatives, and regulatory authorities
4) To retain the essential documents in the Investigator/institution files until the Sponsor informs the Investigator or institution that these documents are no longer needed

INVESTIGATOR SIGNATURE

______________________________________ ________________

Investigator Signature Date

____

Investigator Printed Name

Site Information

Site Number:	
Site Name:	
Site Address:	
